# Synthesis and Antimicrobial Activity of 3-Alkylidene-2-Indolone Derivatives

**DOI:** 10.3390/molecules29225384

**Published:** 2024-11-15

**Authors:** He Huang, Yating Zhang, Qiu Du, Changji Zheng, Chenghua Jin, Siqi Li

**Affiliations:** 1 College of Pharmacy, Yanbian University, Yanji 133002, China; huangher2@163.com (H.H.); 2022051080@ybu.edu.cn (Y.Z.); 2022010883@ybu.edu.cn (Q.D.); jinchenghua@ybu.edu.cn (C.J.); 2 Key Laboratory of Natural Medicines of the Changbai Mountain, Ministry of Education, Yanbian University, Yanji 133002, China

**Keywords:** 3-alkylidene-2-indolone, synthesis, antibiosis, structure activity relationship

## Abstract

The escalating threat of antibiotic-resistant bacteria and fungi underscores an urgent need for new antimicrobial agents. This study aimed to synthesize and evaluate the antimicrobial activities of two series of 3-alkylidene-2-indolone derivatives. We synthesized 32 target compounds, among which 25 exhibited moderate to high antibacterial or antifungal activities. Notably, compounds **10f**, **10g**, and **10h** demonstrated the highest antibacterial activity with a minimum inhibitory concentration (MIC) of 0.5 μg/mL, matching the activity of the positive control gatifloxacin against three Gram-positive bacterial strains: *Staphylococcus aureus* ATCC 6538, 4220, and Methicillin-resistant *Staphylococcus aureus* ATCC 43300. Moreover, the three most active compounds **10f**, **10g**, and **10h** were evaluated for their in vitro cytotoxicity in the HepG2 cancer cell line and L-02; only compound **10h** was found to exert some level of cytotoxicity. These findings suggest that the synthesized 3-alkylidene-2-indolone derivatives hold potential for further development as antibacterial agents.

## 1. Introduction

Microbial infections have historically presented a serious risk to public health, resulting in high rates of mortality and widespread sickness globally [1]. In recent decades, the extensive and improper use of antibiotics has led to a rapid increase in the number and diversity of antibiotic-resistant bacterial strains, presenting a serious challenge to modern medicine. In response to this global trend, the scientific community has focused on designing novel molecular structures with antimicrobial properties, aiming to develop more effective drug formulations to combat this growing threat [2].

**Figure 1:**
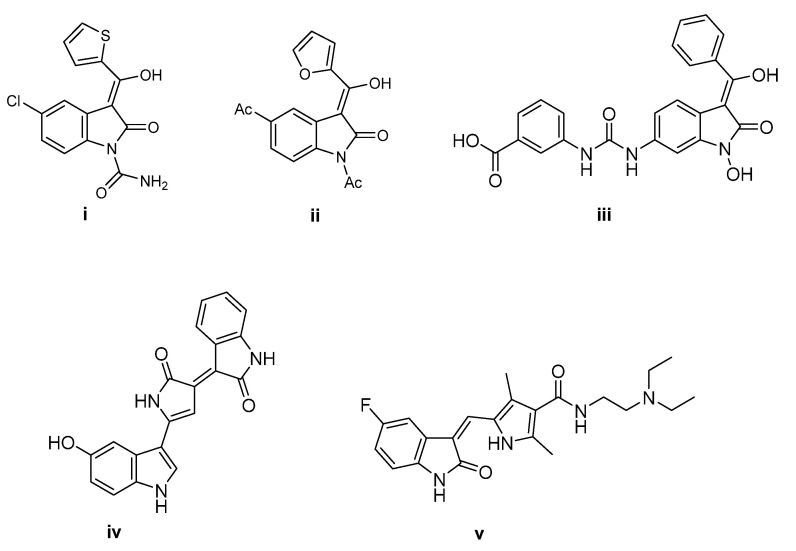
3-Alkylidene-2-indolone-based small molecules with biological activity [4,6,9,20,21].

2-Indolinones are endogenous heteroaromatic organic compounds, consisting of a six-membered benzene ring fused to a five-membered pyrrole ring with a carbonyl group at the C-2 position. These compounds occur naturally in mammalian bodily fluids and tissues, as well as in various plant species. The attachment of an alkyl side chain at the C-3 position forms 3-alkyl-2-indolinone, which can be modified further through synthetic strategies to yield various derivatives. These structures serve as crucial intermediates in organic synthesis [3] and are common structural motifs in numerous drugs and natural products [4,5,6,7]. Compounds in this class are commonly employed as starting materials or pharmacophores in the development of anti-inflammatory [7], anti-cancer [8,9,10,11], antiviral [6], and antibacterial [12,13,14,15,16,17,18,19] drugs (Figure 1).

**Figure 2:**
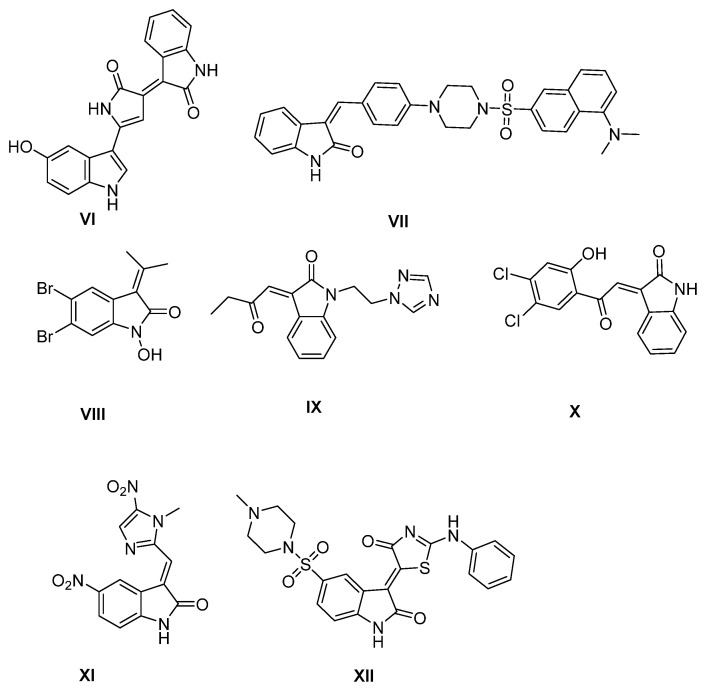
The chemical structures of the reported compounds with antimicrobial activities.

Compound **VI** (violacein, Figure 2) showed antibacterial activity with minimum inhibitory concentration (MIC) values of 6.25 μM against *Staphylococcus aureus* [4]. Rindhe et al. [12] synthesized a series of 3-benzylidene-indolin-2-ones derivatives and found that most of them exhibited significant antimicrobial activity against *Staphylococcus aureus*, *Streptococcus pyogenes*, *Escherichia coli*, *Pseudomonas aeruginosa*, *Aspergillus s niger* and *Aspergillus clavatus*. Li et al. [15] reported the isolation and evaluation of the antibacterial activity of four new brominated indole-related alkaloids from *L. similis*. Among them, compound **VII** exhibited relatively weak inhibitory activity against most of the tested Gram-positive and Gram-negative bacterial strains, with MIC values ranging from 12.5 to 50 μg/mL. Vijai et al. [18] developed a series of isatin-derived azoles as new potential antimicrobial agents, among which compound IX exhibited excellent inhibitory activity against *E. coli* ATCC 25,922 with an MIC value of 1 μg/mL. Compound **X** [19] was able to inhibit the growth of four bacteria and three fungi with MIC values from 12 to 18 μg/mL. Zhou et al. [16] developed a chemical class of hybrids of indolin-2-one, and nitroimidazole possessed a high potency of killing several bacteria strains, including drug-resistant bacteria, in which the compound **XI** exhibited remarkable antibacterial activities with MIC values from 0.0625–4 μg/mL against three Gram-positive bacteria strains and two Gram-negative bacteria strains. Compound **XII**, as an indolin-2-one derivative, exhibited promising antibacterial activity against both Gram-positive and Gram-negative bacteria, as well as moderate antifungal activity. Additionally, **XII** displayed the selectivity to DHFR inhibitory activity closely to the methotrexate and could effectively bind to dihydrofolate reductase (DHFR, PDB: 1DLS) [22]. These instances imply 3-alkylidene-2-indolone as a promising antibacterial scaffold and the possible mechanisms of action.

Although a large number of molecules with a 3-alkylidene-2-indolone skeleton have been shown to possess antibacterial activities, most of these compounds only display moderate antimicrobial activities. Moreover, previous studies on indolinone derivatives have not conducted systematic investigations of simple substituent modifications. This situation restricts the analysis and conclusions regarding their structure–activity relationships and makes them unsuitable for drug development.

In this report, two series of 3-alkyl-2-indolinone derivatives were synthesized from the appropriate α-diazo-β-ketoanilides with water as the solvent and Cu(NO_3_)_2_·3H_2_O as the catalyst [23]. The structure of the obtained molecules was confirmed by nuclear magnetic resonance (NMR) spectra and high-resolution mass spectrometry (HRMS). The antimicrobial activity of the newly synthesized compounds was determined in vitro by using the broth microdilution method against reference strains of Gram-negative, Gram-positive bacteria and a fungus.

## 2. Results and Discussion

### 2.1. Chemistry

**Scheme 1:**
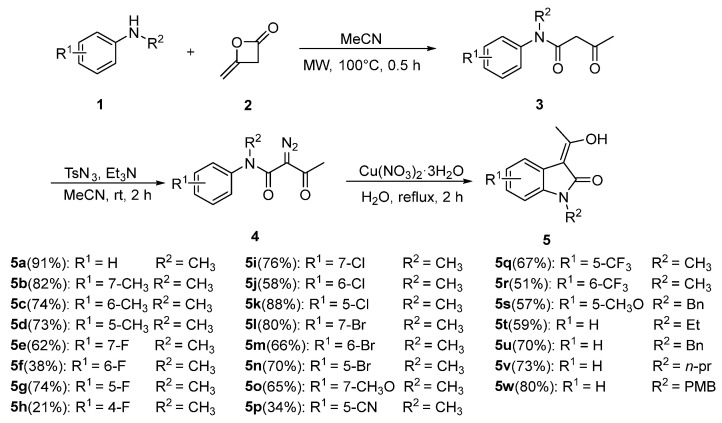
Synthesis of compounds **5a**–**w**.

**Scheme 2:**
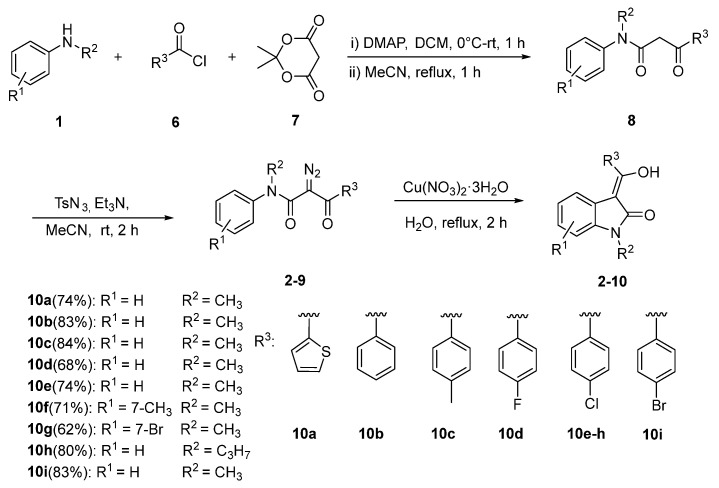
Synthesis of compounds **10a**–**i**.

The general synthetic routes for the preparation of two series of 3-alkyl-2-indolinone derivatives (compounds **5a**–**w** and **10a**–**i**), as depicted in Scheme 1 and Scheme 2, are in line with the method reported by Xu and coworkers, employing a convenient and environmentally friendly procedure for the synthesis of 3-alkylideneoxindoles from α-diazo-β-ketoanilides, utilizing water as the solvent and Cu(NO_3_)_2_·3H_2_O as the catalyst [23]. The acetylacetone was attacked by substituted anilines to produce dicarbonyl compounds **3a**–**w** under microwave conditions. The significant intermediates α-diazo-β-ketoanilides **4a**–**w** were prepared from the corresponding dicarbonyl compounds **3a**–**w** with TsN_3_ in good to excellent yields. The target compounds **5a**–**w** were carried out by **4a**–**w** with Cu(NO_3_)_2_·3H_2_O as the catalyst in water, with yields ranging from 34% to 91% (Scheme 1). The application of microwave technology makes reactions more convenient and rapid compared to traditional methods [23], reducing the reaction time from overnight to 0.5 h, thereby allowing the entire reaction sequence to be completed within one day.

The synthesis route for the other series 3-alkyl-2-indolinone derivatives **10a**–**i** (62–84% yields) was similar to that of the **5a**–**w**, with the difference being that the corresponding dicarbonyl compounds **8** for the diazo intermediates **9** were obtained from the reaction of substituted anilines **1**, acyl chlorides **6**, and Meldrum’s acid **7** (Scheme 2).

Among the thirty-two cyclopropane amide derivatives synthesized in this study, sixteen compounds were new compounds, and sixteen compounds were known compounds, of which the ^1^H NMR and ^13^C NMR spectra were consistent with those reported in the literature [23,24,25,26,27,28,29]. In ^1^H NMR spectra, all the compounds showed one broad singlet signal in the ranges of *δ*_H_ 13.36–14.44 ppm due to H-O. It indicates the presence of an intramolecular hydrogen bond between the hydroxyl group and the carbonyl group of the indolinone ring. Based on this observation, we can affirm that the products’ structure must be the Z configuration, as it is the only configuration capable of forming such an intramolecular hydrogen bond.

### 2.2. Evaluation of the Antimicrobial Activity

In this study, all target compounds (**5a**–**w** and **10a**–**i**) were selected and evaluated for their antimicrobial activity against seven common pathogenic strains (three Gram-positive bacteria strains: *Staphylococcus aureus* ATCC 6538 and 4220, and Methicillin-resistant *Staphylococcus aureus* ATCC 43300; three Gram-negative bacteria strains: *Pseudomonas aeruginosa* CMCC 10211, *Escherichia coli* ATCC 25922 and 1924; and one fungus: *Candida albicans* CMCC 98001). Gatifloxacin and fluconazole were used as positive control drugs in this experiment.

The in vitro antimicrobial activity of the target compounds (**5a**–**w** and **10a**–**i**)) is shown in Table 1. Among the 32 synthesized target compounds, 25 compounds exhibited moderate or high antimicrobial activity against different strains. Generally, the antimicrobial activity of compounds **10a**–**i** was superior to that of compounds **5a**–**w**. The introduction of an aromatic heterocycle at the R^3^ position significantly enhanced the antimicrobial activity. For the Gram-positive bacteria 4220, compounds **5d**, **5e**, **5k**, **5u** and **10c**–**10i** (MIC = 0.5–16 μg/mL) exhibited moderate to strong antibacterial activity. In the **10** series of compounds, **10g** showed high antibacterial activity (MIC = 0.5 μg/mL). For the Gram-positive bacteria ATCC 6538, compounds **5j**, **5q**, **5r**, **5u** and**10b**–**10i** (MIC = 0.5–16 μg/mL) showed moderate to strong antibacterial activity. Compounds **5r**, **5v**, **10v** and **10e**–**10i** showed moderate to strong antibacterial activity against the Methicillin-resistant ATCC 43300 at concentrations of 0.5–16 μg/mL, among which compounds **10g** and **10h** showed similarly high antibacterial activity to the comparable drug gatifloxacin (MIC = 0.5 μg/mL). For the Gram-negative bacteria CMCC 10211 and ATCC 25922, except for **10h** (MIC = 16 μg/mL) which showed moderate activity, all compounds showed weak or no activity. Compounds **5c**–**5e**, **5i**–**5o**, **5q**–**5s**, **5u**–**5w** and **10b**–**10i** showed moderate to high antibacterial activity against Gram-negative bacteria 1924. For the fungus CMCC 98001, **5d, 5h, 5r** and **10d** (MIC = 8–16 μg/mL) showed moderate antifungal activity. Among the two series of compounds, the **10** series exhibited stronger antimicrobial activities than the **5** series.

Moreover, minimum bactericidal concentration (MBC (μg/mL)) or minimum fungicidal concentration (MFC (μg/mL)) assays were performed for the promising compounds **10d**, **10f**, **10g** and **10h** against *Staphylococcus aureus* ATCC 6538, *Staphylococcus aureus* 4220, *Staphylococcus aureus* ATCC 43300 and *Candida albicans* CMCC 98001, and shown in Table 2. The MBC/MIC values of compounds **10f**, **10g** and **10h** were greater than 4, indicating that the antibacterial properties of the compounds were reversible and the bacteria were susceptible to resistance and their bacteriostatic impact against *Staphylococcus aureus* ATCC 6538, *Staphylococcus aureus* 4220 and *Staphylococcus aureus* ATCC 43300, respectively. The MFC/MIC value of compound **10d** was 2 and revealed its fungicidal activity against *Candida albicans* CMCC 98001.

Based on the above activity results, the following structure activity relationships (SAR) could be summarized: (1) The R^1^ position of the compounds generally exhibits greater activity with electron-withdrawing substituents compared to electron-donating substituents. Modifications at this position can enhance selectivity against *Escherichia coli* 1924 and *Candida albicans* CMCC 98001. (2) Introducing a phenyl ring or extending the straight-chain alkane at the R^2^ position can improve antimicrobial activity. (3) The introduction of a phenyl ring or halogen-substituted phenyl ring at the R^3^ position can significantly enhance antimicrobial activity, with chlorination providing the most effective results.

### 2.3. Cytotoxic Activity

The cytotoxicity of the most bioactive compounds **10f**, **10g** and **10h** were evaluated in vitro against the HepG2 cell line (human hepatocellular carcinoma cell line) and the L-02 cell line (normal liver cell line) by 3-(4,5-dimethylthiazol-2-yl)-2,5-diphenyltetrazolium bromide (MTT) assay. The obtained results were presented by the inhibitory concentration that reduces the viability of the cell population to 50% of the maximal viability (IC_50_). As shown in Table 3, compounds **10f** (IC_50_ > 100 µM) and **10g** (IC_50_ > 100µM) showed no obvious cytotoxicity against HepG2 and L-02 cells. Compound **10h** exerted moderate cytotoxicity with IC_50_ = 41.6 µM against HepG2 cells and IC_50_ = 53.5 µM in L-02 cells. These results suggested the value for further study of these compounds as antimicrobial agents.

### 2.4. Docking

**Figure 3:**
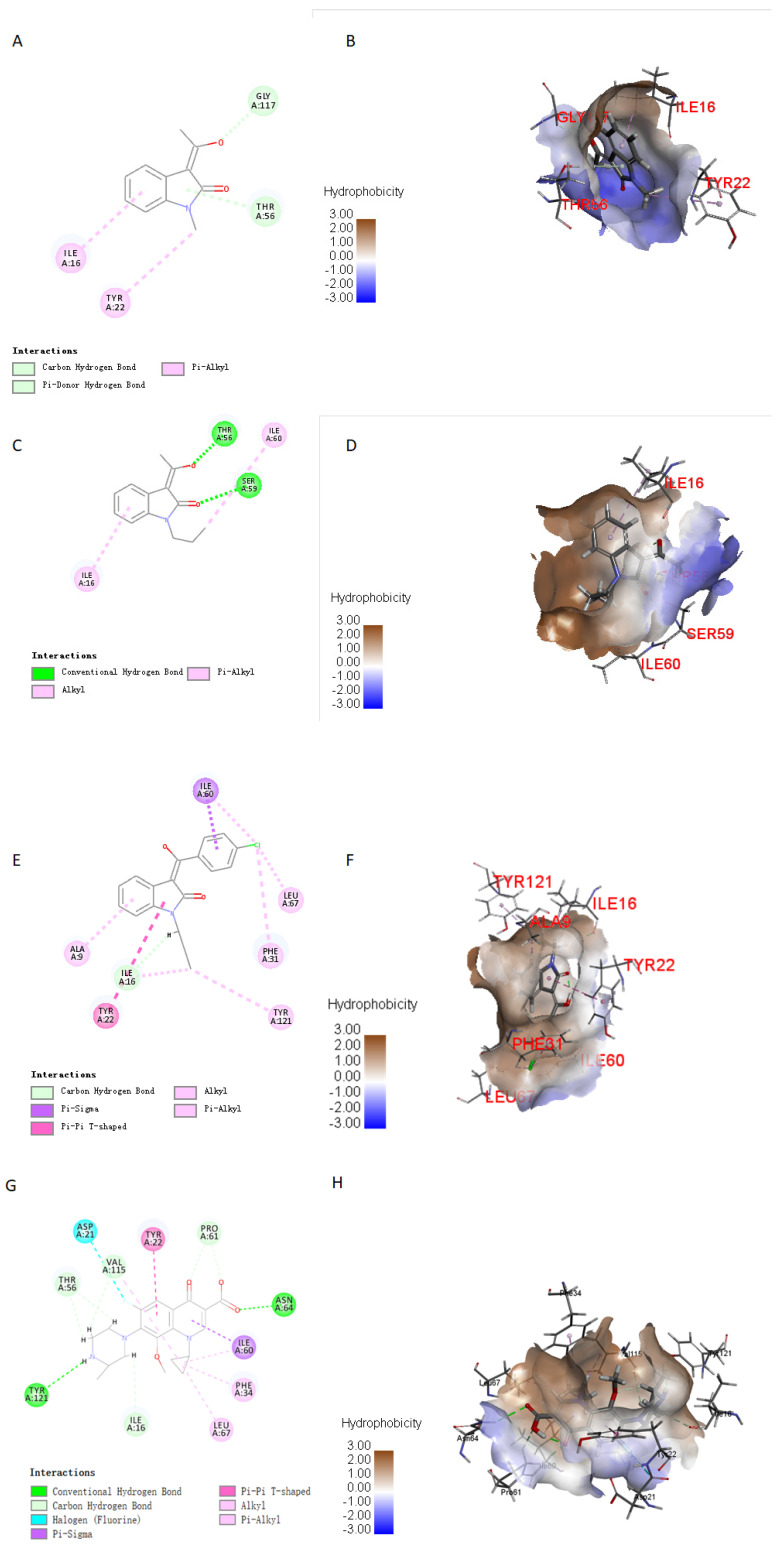
Docking pose of compounds **5a**, **5v, 10h** and gatifloxacin in the active site of DHFR (PDB ID: 1DLS). (**A**) 2D binding mode of **5a**. (**B**) 3D binding mode of **5a** in the binding pocket of DHFR. (**C**) 2D binding mode of **5v**. (**D**) 3D binding mode of **5v** in the binding pocket of DHFR. (**E**) 2D binding mode of **10h**. (**F**) 3D binding mode of **10h** in the binding pocket of DHFR. (**G**) 2D binding mode of gatifloxacin. (**H**) 3D binding mode of gatifloxacin in the binding pocket of DHFR.

Dihydrofolate reductase (DHFR) has long been recognized as a key therapeutic target for combating bacterial infections, given its crucial role in the synthesis of nucleic acids and amino acids. The inhibition of DHFR leads to a reduction in the tetrahydrofolate necessary for the production of pyrimidines and purines, which are essential for DNA and RNA synthesis. As a result, the inhibition halts RNA and DNA synthesis, thereby disrupting bacterial structure or impairing their capacity to divide and reproduce [30]. Alzahrani and colleagues have reported on the antimicrobial activities of 3-alkylidene-2-indolone derivatives, highlighting their potential as DHFR inhibitors, and have further supported their findings with a molecular modeling study by docking with 1DLS [22]. To investigate the possible mechanism of the antimicrobial action of compounds, CDocker was used to determine the binding patterns of the three representative compounds **5a**, **5v**, **10h,** positive control drug gatifloxacin and 1DLS [22]. As shown in Figure 3A,C, **5a** and **5v** interacted with four amino acid residues (Thr56, Ile60, Ser59 and Ile16) of the receptor protein in three different ways. However, the propyl group at the N-1 position of compound **5v** forms a relatively strong classical hydrogen bond with Thr56. Although both compounds have the same number of binding sites, their binding affinities differ slightly, which was consistent with the results of the antimicrobial activity results. The pyrrole and phenyl rings of the indolinone core in compound **10h** interacted with Tyr22 and Ala9 through π-π T-shaped interactions and π-alkyl interactions (Figure 3E). The propyl group at the N-1 position formed auxiliary alkyl van der Waals interactions with Tyr121 and Ile16, while the phenyl ring at C3 and the para-chlorine formed π-σ hyperconjugation with Ile60. The para-chlorine also interacted with Phe31 and Leu67 through alkyl van der Waals forces. Thus, compound **10h** interacted with seven amino acid residues surrounding the substrate protein through five different types of force field binding. Compared to compounds **5a** and **5v** in the **5** series, the number of binding sites and binding affinities significantly improved. The antimicrobial activity results suggested that modifications at the alkyl sites are crucial for enhancing the pharmacological activity of 3-(1-hydroxyalkyl)indolinone derivatives.

## 3. Materials and Methods

### 3.1. General Chemistry Section

All commercially available reagents and solvents were purchased from Aladdin (Shanghai, China), Bidepharm (Shanghai, China) and Macklin (Shanghai, China), and used without further purification. The melting point is measured using the fully automatic melting point tester MP120 from Haineng Company (Jinan, China) in an open glass capillary tube. All microwave reactions were conducted in a CEM Discover 2.0 microwave system equipped with an infrared temperature detector (CEM Corporation, Matthews, NC, USA). All reactions were monitored for completion by TLC using Merck silica gel 60 F_254_ glass plates (Haiyang, Qingdao, China). ^1^H-NMR and ^13^C NMR spectra were recorded on Bruker NMR spectrometers (Bruker, Rheinstetten, Germany) at 300 MHz or 500 MHz; tetramethyl silane (TMS) was used as an internal standard. Chemical shifts were signified in ppm (*δ*) relative to internal TMS in CDCl_3_ or DMSO-*d*_6_, and coupling constants (*J*) are in hertz (Hz). High- resolution mass spectra (HRMS-ESI, HRMS-APCI) were obtained on a Thermo Scientific LTQ Orbitrap XL (Theromo Fisher Scientific, Waltham, MA, USA).

### 3.2. Experimental Section

#### 3.2.1. General Procedure of 3-Alkylidene-2-Indolones **5a**–**w**

Aniline **1** (10 mmol) was added to a solution of diketene **2** (10 mmol) in acetonitrile, and the resulting solution was heated by microwave at 100 °C for 0.5 h to obtain the acetoacetylated intermediate **3**. Then, *p*-toluenesulfonyl azide (12 mmol) and triethylamine (12 mmol) were added and mixed, and stirred for 2 h at room temperature. The solution was concentrated under reduced pressure, and the resulting residue was purified by silica gel column chromatography to give pure α-diazo-β-ketoanilides **4**. To a solution of Cu(NO_3_)_2_·3H_2_O (0.05 mmol) in water (10 mL), diazo compound **4** (1 mmol) was added. The suspension was heated to reflux, and the progress of the reaction was monitored by TLC until complete consumption of the starting materials was observed. Ethyl acetate (10 mL×2) was added to extract the organic phase, and the organic phase was dried with anhydrous Na_2_SO_4_, filtered, and concentrated in vacuo. The residue was purified by flash column chromatography on silica gel (PE/EtOAc) to obtain pure 3-alkylidene-2-indolones **5**.

**(*Z*)-3-(1-hydroxyethylidene)-1-methylindolin-2-one** (**5a**) [27]. (DCM:MeOH = 150:1, R*_f_* = 0.38); Yield: 91%; Purple crystals; mp: 95–97 °C; Lit. [24] mp: 90–92 °C. ^1^H NMR (300 MHz, Chloroform-*d*) *δ* 13.43 (br. s, 1H), 7.40–7.32 (m, 1H), 7.25–7.17 (m, 1H), 7.15–7.06 (m, 1H), 6.94 (d, *J* = 7.8 Hz, 1H), 3.34 (s, 3H), 2.45 (s, 3H). ^13^C NMR (75 MHz, Chloroform-d) *δ* 173.0, 171.1, 139.0, 125.3, 122.3, 122.2, 119.8, 108.5, 101.9, 76.7, 25.8, 20.4.
**(*Z*)-3-(1-hydroxyethylidene)-1,7-dimethylindolin-2-one** (**5b**) [25]. (DCM:MeOH = 150:1, R*_f_* = 0.37); Yield: 82%; Purple crystals; mp: 101.4–106.4 °C; Lit. [25] mp: 94 °C. ^1^H NMR (300 MHz, Chloroform-*d*) *δ* 13.91 (br. s, 1H), 7.2 (dd, *J* = 7.1, 1.7 Hz, 1H), 7.0–6.9 (m, 1H), 6.9–6.9 (m, 1H), 3.6 (s, 3H), 2.6 (s, 3H), 2.4 (s, 3H). ^13^C NMR (75 MHz, Chloroform-*d*) *δ* 173.1, 171.6, 136.9, 129.0, 123.0, 122.1, 120.3, 117.9, 101.7, 29.1, 20.5, 19.4.
**(*Z*)-3-(1-hydroxyethylidene)-1,6-dimethylindolin-2-one** (**5c**) [25]. (DCM:MeOH = 150:1, R*_f_* = 0.36);Yield: 74%; Purple crystals; mp: 149–152 °C; ^1^H NMR (300 MHz, Chloroform-*d*) *δ* 13.39 (br. s, 1H), 7.23 (d, *J* = 7.8 Hz, 1H), 6.91 (d, *J* = 7.7 Hz, 1H), 6.76 (s, 1H), 3.32 (s, 3H), 2.44–2.39 (m, 6H). ^13^C NMR (75 MHz, Chloroform-*d*) *δ* 171.8, 171.4, 139.3, 135.5, 122.9, 119.7, 119.6, 109.4, 102.0, 25.8, 21.9, 20.3.
**(*Z*)-3-(1-hydroxyethylidene)-1,5-dimethylindolin-2-one** (**5d**) [25]. (DCM:MeOH = 150:1, R*_f_* = 0.37); Yield: 73%; Purple crystals; mp: 128.9–130.9 °C; Lit. [25] mp: 90 °C. ^1^H NMR (300 MHz, Chloroform-*d*) *δ* 13.46 (br. s, 1H), 7.17 (s, 1H), 7.02 (d, *J* = 7.8 Hz, 1H), 6.83 (d, *J* = 7.9 Hz, 1H), 3.31 (s, 3H), 2.44 (s, 3H), 2.39 (s, 3H). ^13^C NMR (75 MHz, Chloroform-*d*) *δ* 171.7, 170.2, 136.0, 130.6, 124.8, 121.4, 119.6, 107.2, 100.9, 24.8, 20.6, 19.4.
**(*Z*)-7-fluoro-3-(1-hydroxyethylidene)-1-methylindolin-2-one** (**2**–**5e**). (DCM:MeOH = 150:1, R*_f_* = 0.37); Yield: 62%; Purple crystals; mp: 122.1–124.1 °C; ^1^H NMR (300 MHz, Chloroform-*d*) *δ* 13.79 (br. s, 1H), 7.17–7.08 (m, 1H), 7.05–6.86 (m, 2H), 3.55 (d, *J* = 2.7 Hz, 3H), 2.45 (s, 3H). ^13^C NMR (75 MHz, Chloroform-*d*) *δ* 174.5, 170.9, 148.7 (d, *J* = 242.4 Hz), 125.5 (d, *J* = 4.8 Hz), 125.4, 122.6 (d, *J* = 6.8 Hz), 115.6 (d, *J* = 3.2 Hz), 112.8 (d, *J* = 19.1 Hz), 101.8 (d, *J* = 2.8 Hz), 28.4 (d, *J* = 5.8 Hz), 20.6; HRMS (ESI) *m/z* calcd for C_11_H_10_FNO_2_Na^+^230.0588 [M+Na]^+^, found 230.0590[M+Na]^+^.
**(*Z*)-6-fluoro-3-(1-hydroxyethylidene)-1-methylindolin-2-one** (**5f**). (DCM:MeOH = 150:1, R*_f_* = 0.39); Yield: 38%; Purple crystals; mp: 109.2–113.7 °C; ^1^H NMR (300 MHz, Chloroform-*d*) *δ* 14.44 (br. s, 1H), 7.21–7.11 (m, 1H), 6.85–6.71 (m, 2H), 3.35 (s, 3H), 2.57 (d, *J* = 2.4 Hz, 3H). ^13^C NMR (75 MHz, Chloroform-*d*) *δ* 176.5 (d, *J* = 2.2 Hz), 171.1, 155.2 (d, *J* = 245.5 Hz), 140.6 (d, *J* = 10.1 Hz), 126.4 (d, *J* = 9.0 Hz), 109.9 (d, *J* = 23.6 Hz), 109.2 (d, *J* = 19.0 Hz), 104.6 (d, *J* = 3.0 Hz), 99.9 (d, *J* = 2.3 Hz), 26.3, 21.8 (d, *J* = 15.1 Hz); HRMS (ESI) *m/z* calcd for C_11_H_10_FNO_2_Na^+^ 230.0588[M+Na]^+^, found 230.0588[M+Na]^+^.
**(*Z*)-5-fluoro-3-(1-hydroxyethylidene)-1-methylindolin-2-one** (**5g**) [25]. (DCM:MeOH = 150:1, R*_f_* = 0.38); Yield: 74%; Purple crystals; mp: 169.5–171.5 °C; Lit. [25] mp: 137 °C. ^1^H NMR (300 MHz, Chloroform-*d*) *δ* 13.60 (br. s, 1H), 7.08 (dd, J = 9.1, 2.3 Hz, 1H), 6.96–6.81 (m, 2H), 3.33 (s, 3H), 2.43 (s, 3H). ^13^C NMR (75 MHz, Chloroform-*d*) *δ* 174.3, 171.1, 159.3 (d, *J* = 237.8 Hz), 135.0, 123.4 (d, *J* = 9.6 Hz), 111.5 (d, *J* = 24.1 Hz), 108.8 (d, *J* = 9.0 Hz), 107.4 (d, *J* = 26.2 Hz), 101.9 (d, *J* = 2.8 Hz), 25.9, 20.4.
**(*Z*)-4-fluoro-3-(1-hydroxyethylidene)-1-methylindolin-2-one** (**5h**). (DCM:MeOH = 150:1, R*_f_* = 0.37); Yield: 21%; Purple crystals; mp: 107.7–111.1 °C; ^1^H NMR (300 MHz, Chloroform-*d*) *δ* 13.36 ((br. s, 1H), 7.24 (d, *J* = 5.1 Hz, 1H), 6.84–6.75 (m, 1H), 6.67 (dd, *J* = 8.9, 2.3 Hz, 1H), 3.31 (s, 3H), 2.42 (s, 3H). ^13^C NMR (75 MHz, Chloroform-*d*) *δ* 172.4 (d, *J* = 1.9 Hz), 171.6, 161.5 (d, *J* = 242.8 Hz), 140.1 (d, *J* = 11.3 Hz), 120.4 (d, *J* = 9.3 Hz), 118.2 (d, *J* = 2.5 Hz), 108.6 (d, *J* = 22.6 Hz), 101.4, 97.1 (d, *J* = 27.4 Hz), 25.9, 20.3; HRMS (ESI) *m/z* calcd for C_11_H_10_FNO_2_Na^+^ 230.0588[M+Na]^+^, found 230.0588[M+Na]^+^.
**(*Z*)-7-chloro-3-(1-hydroxyethylidene)-1-methylindolin-2-one** (**5i**) [26]. (DCM:MeOH = 150:1, R*_f_* = 0.39); Yield: 76%; purple crystals; mp: 140.9–142.9 °C; ^1^H NMR (300 MHz, Chloroform-*d*) *δ* 13.94 (br. s, 1H), 7.24 (s, 1H), 7.16–7.09 (m, 1H), 7.04–6.93 (m, 1H), 3.71 (s, 3H), 2.46 (s, 3H). ^13^C NMR (75 MHz, Chloroform-*d*) *δ* 174.6, 171.4, 134.5, 127.3, 125.2, 122.8, 118.2, 116.4, 101.2, 29.2, 20.7.
**(*Z*)-6-chloro-3-(1-hydroxyethylidene)-1-methylindolin-2-one** (**5j**). (DCM:MeOH = 150:1, R*_f_* = 0.38); Yield: 58%; purple crystals; mp: 109.6–111.6 °C; ^1^H NMR (300 MHz, Chloroform-*d*) *δ* 13.44 (br. s, 1H), 7.26–7.21 (m, 1H), 7.09–7.03 (m, 1H), 6.92 (d, *J* = 1.8 Hz, 1H), 3.31 (s, 3H), 2.42 (s, 3H). ^13^C NMR (75 MHz, Chloroform-*d*) *δ* 173.5, 171.3, 139.9, 131.0, 122.1, 120.8, 120.4, 109.1, 101.3, 25.9, 20.5; HRMS (ESI) *m/z* calcd for C_11_H_10_ClNO_2_Na^+^ 246.0292[M+Na]^+^, found 246.0292[M+Na]^+^.
**(*Z*)-5-chloro-3-(1-hydroxyethylidene)-1-methylindolin-2-one** (**5k**) [25]. (DCM:MeOH = 150:1, R*_f_* = 0.37); Yield: 88%; purple crystals; mp: 89.7–91.1 °C; Lit. [25] mp: 90–92 °C.^1^H NMR (300 MHz, Chloroform-*d*) *δ* 13.36 (br. s, 1H), 7.30 (d, *J* = 2.0 Hz, 1H), 7.20–7.15 (m, 1H), 6.84 (d, *J* = 8.4 Hz, 1H), 3.32 (s, 3H), 2.44 (s, 3H). ^13^C NMR (75 MHz, Chloroform-*d*) *δ* 174.4, 171.0, 137.3, 127.7, 125.0, 123.7, 119.8, 109.2, 101.3, 25.9, 20.5.
**(*Z*)-7-bromo-3-(1-hydroxyethylidene)-1-methylindolin-2-one** (**5l**) [26]. (DCM:MeOH = 150:1, R*_f_* = 0.37); Yield: 80%; purple crystals; mp: 102–104 °C; Lit. [26] mp: 122–124 °C. ^1^H NMR (300 MHz, Chloroform-*d*) *δ* 13.80 (br. s, 1H), 7.35–7.26 (m, 2H), 6.99–6.87 (m, 1H), 3.73 (s, 3H), 2.46 (s, 3H). ^13^C NMR (75 MHz, Chloroform-*d*) *δ* 174.6, 171.6, 135.9, 130.6, 125.5, 123.2, 118.7, 103.3, 101.2, 29.4, 20.8.
**(*Z*)-6-bromo-3-(1-hydroxyethylidene)-1-methylindolin-2-one** (**5m**). (DCM:MeOH = 150:1, R*_f_* = 0.35); Yield: 66%; purple crystals; mp: 118–123 °C; ^1^H NMR (300 MHz, Chloroform-*d*) *δ* 13.43 (br. s, 1H), 7.24–7.15 (m, 2H), 7.07 (d, *J* = 1.3 Hz, 1H), 3.31 (s, 3H), 2.42 (s, 3H). ^13^C NMR (75 MHz, Chloroform-*d*) *δ* 173.7, 171.1, 140.0, 125.0, 121.3, 120.7, 118.5, 111.9, 101.4, 25.9, 20.6; HRMS (ESI) *m/z* calcd for C_11_H_10_BrNO_2_Na^+^ 289.9787[M+Na]^+^, found 289.9788[M+Na]^+^.
**(*Z*)-5-bromo-3-(1-hydroxyethylidene)-1-methylindolin-2-one** (**5n**). (DCM:MeOH = 150:1, R*_f_* = 0.38); Yield: 70%; purple crystals; mp: 135.7–138.7 °C; ^1^H NMR (300 MHz, Chloroform-*d*) *δ* 13.43 (br. s, 1H), 7.44 (d, *J* = 1.9 Hz, 1H), 7.32 (dd, *J* = 8.3, 1.9 Hz, 1H), 6.80 (d, *J* = 8.3 Hz, 1H), 3.31 (s, 3H), 2.44 (s, 3H). ^13^C NMR (75 MHz, Chloroform-*d*) *δ* 174.5, 170.8, 137.8, 127.8, 124.2, 122.6, 115.1, 109.8, 101.2, 25.9, 20.6; HRMS (ESI) *m/z* calcd for C_11_H_10_BrNO_2_Na^+^ 289.9787[M+Na]^+^, found 289.9788[M+Na]^+^.
**(*Z*)-3-(1-hydroxyethylidene)-7-methoxy-1-methylindolin-2-one** (**5o**) [26]. (DCM:MeOH = 150:1, R*_f_* = 0.37); Yield: 65%; purple crystals; mp: 143.8–146 °C; Lit. [24] mp: 80–90 °C.^1^H NMR (300 MHz, Chloroform-*d*) *δ* 13.96 (br. s, 1H), 7.06–6.97 (m, 2H), 6.81–6.73 (m, 1H), 3.87 (s, 3H), 3.62 (s, 3H), 2.44 (s, 3H). ^13^C NMR (75 MHz, Chloroform-*d*) *δ* 173.8, 171.0, 146.1, 126.8, 124.0, 122.6, 113.0, 108.9, 102.1, 56.0, 29.2, 20.5.
**(*Z*)-3-(1-hydroxyethylidene)-1-methyl-2-oxoindoline-5-carbonitrile** (**5p**). (DCM:MeOH = 150:1, R*_f_* = 0.39); Yield: 34%; purple crystals; mp: 145.1–147.1 °C; ^1^H NMR (500 MHz, Chloroform-*d*) *δ* 13.39 (br. s, 1H), 7.60 (d, *J* = 1.5 Hz, 1H), 7.55–7.50 (m, 1H), 7.01 (d, *J* = 8.1 Hz, 1H), 3.38 (s, 3H), 2.49 (s, 3H). ^13^C NMR (75 MHz, Chloroform-*d*) *δ* 175.8, 171.3, 141.8, 129.9, 123.1, 122.9, 119.8, 108.9, 105.4, 100.6, 26.1, 20.8; HRMS (ESI) *m/z* calcd for C_12_H_10_N_2_O_2_Na^+^ 237.0634[M+Na]^+^, found 237.0635[M+Na]^+^.
**(*Z*)-3-(1-hydroxyethylidene)-1-methyl-5-(trifluoromethyl)indolin-2-one** (**5q**) [24]. (DCM:MeOH = 150:1, R*_f_* = 0.39); Yield: 67%; purple crystals; mp: 144.3–147.1 °C; Lit. [24] mp: 102–104 °C.^1^H NMR (300 MHz, Chloroform-*d*) *δ* 13.60 (br. s, 1H), 7.55 (s, 1H), 7.52–7.46 (m, 1H), 7.01 (d, *J* = 8.2 Hz, 1H), 3.38 (s, 3H), 2.50 (s, 3H). ^13^C NMR (75 MHz, Chloroform-*d*) *δ* 174.9, 171.4, 141.2, 124.8 (q, *J* = 270 Hz),124.7, 124.3, 122.8–122.5 (m), 116.6–116.4 (m), 108.2, 101.2, 26.0, 20.7.
**(*Z*)-3-(1-hydroxyethylidene)-1-methyl-6-(trifluoromethyl)indolin-2-one** (**5r**). (DCM:MeOH = 150:1, R*_f_* = 0.38); Yield: 51%; purple crystals; mp: 109.4–111.4 °C; ^1^H NMR (300 MHz, Chloroform-*d*) *δ* 13.84 (s, 1H), 7.47–7.34 (m, 2H), 7.16 (s, 1H), 3.39 (s, 3H), 2.50 (s, 3H). ^13^C NMR (75 MHz, Chloroform-*d*) *δ* 175.7, 171.2, 138.8, 127.3 (q, *J* = 32.6 Hz), 127.0 (q, *J* = 270 Hz),125.7, 119.5, 119.3 (d, *J* = 4.0 Hz), 105.3 (d, *J* = 3.8 Hz), 101.3, 26.0, 20.7; HRMS (APCI) *m/z* calcd for C_12_H_11_F_3_NO_2_^+^258.0736[M+H]^+^, found 258.0733[M+H]^+^.
**(*Z*)-1-benzyl-3-(1-hydroxyethylidene)-5-methoxyindolin-2-one** (**5s**). (DCM:MeOH = 150:1, R*_f_* = 0.37); Yield: 57%; white crystals; mp: 109.1–111.1 °C; ^1^H NMR (300 MHz, Chloroform-*d*) *δ* 13.74 (br. s, 1H), 7.35–7.22 (m, 6H), 6.97 (d, *J* = 2.4 Hz, 1H), 6.75–6.70 (m, 1H), 6.68–6.63 (m, 1H), 5.02 (s, 2H), 3.80 (s, 3H), 2.46 (s, 3H). ^13^C NMR (75 MHz, Chloroform-*d*) *δ* 173.6, 171.2, 155.8, 136.2, 132.3, 128.9 (2C), 127.7, 127.3 (2C), 123.6, 109.7, 109.6, 107.5, 102.0, 56.0, 43.5, 20.5; HRMS (ESI) *m/z* calcd for C_18_H_17_NO_3_Na^+^ 318.1101[M+Na]^+^, found 318.1103[M+Na]^+^.
**(*Z*)-1-ethyl-3-(1-hydroxyethylidene) indolin-2-one** (**5t**). (DCM:MeOH = 150:1, R*_f_* = 0.36); Yield: 59%; purple crystals; mp: 149.8–151.8 °C; ^1^H NMR (300 MHz, Chloroform-*d*) *δ* 13.67 (br. s, 1H), 7.38 (d, *J* = 7.5 Hz, 1H), 7.25–7.18 (m, 1H), 7.14–7.06 (m, 1H), 6.98 (d, *J* = 8.3 Hz, 1H), 3.91 (q, *J* = 7.2 Hz, 2H), 2.46 (s, 3H), 1.32 (t, *J* = 7.2 Hz, 3H). ^13^C NMR (75 MHz, Chloroform-*d*) *δ* 173.1, 170.8, 138.0, 125.2, 122.6, 122.0, 120.0, 108.7, 101.9, 34.4, 20.5, 13.4; HRMS (ESI) *m/z* calcd for C_12_H_13_NO_2_Na^+^ 226.0838[M+Na]^+^, found 226.0843[M+Na]^+^.
**(*Z*)-1-benzyl-3-(1-hydroxyethylidene)indolin-2-one** (**5u**) [25]. (DCM:MeOH = 150:1, R*_f_* = 0.37); Yield: 70%; purple crystals; mp: 127.9–130.9 °C; Lit. [23] mp: 127–129 °C.^1^H NMR (300 MHz, Chloroform-*d*) *δ* 13.59 (br. s, 1H), 7.39 (d, *J* = 6.7 Hz, 1H), 7.33–7.26 (m, 5H), 7.16–7.04 (m, 2H), 6.86 (d, *J* = 7.2 Hz, 1H), 5.06 (s, 2H), 2.49 (s, 3H). ^13^C NMR (75 MHz, Chloroform-*d*) *δ* 173.4, 171.2, 138.2, 136.2, 128.9 (2C), 127.7, 127.3 (2C), 125.3, 122.5, 122.3, 119.9, 109.5, 101.8, 43.4, 20.5.
**(*Z*)-3-(1-hydroxyethylidene)-1-propylindolin-2-one** (**5v**). (DCM:MeOH = 150:1, R*_f_* = 0.37); Yield: 73%; purple crystals; mp: 258.9–260.9 °C; ^1^H NMR (300 MHz, Chloroform-*d*) *δ* 13.65 (br. s, 1H), 7.38 (d, *J* = 6.5 Hz, 1H), 7.24–7.17 (m, 1H), 7.13–7.06 (m, 1H), 6.97 (d, *J* = 7.4 Hz, 1H), 3.81 (dd, *J* = 7.9, 6.6 Hz, 2H), 2.46 (s, 3H), 1.76 (h, *J* = 7.4 Hz, 2H), 0.97 (t, *J* = 7.4 Hz, 3H). ^13^C NMR (75 MHz, Chloroform-*d*) *δ* 173.2, 171.2, 138.4, 125.2, 122.5, 122.0, 119.9, 108.8, 101.8, 41.3, 21.5, 20.5, 11.5; HRMS (ESI) *m/z* calcd for C_13_H_15_NO_2_Na^+^ 240.0995[M+Na]^+^, found 240.0995[M+Na]^+^.
**(*Z*)-3-(1-hydroxyethylidene)-1-(4-methoxybenzyl)indolin-2-one** (**5w**) [25]. (DCM:MeOH = 150:1, R*_f_* = 0.39); purple crystals; Yield: 80%; mp: 119.7–121.7 °C; Lit. [25] mp: 136 °C. ^1^H NMR (300 MHz, Chloroform-*d*) *δ* 13.62 (br. s, 1H), 7.40–7.35 (m, 1H), 7.25–7.20 (m, 2H), 7.16–7.03 (m, 2H), 6.91–6.81 (m, 3H), 4.99 (s, 2H), 3.76 (s, 3H), 2.48 (s, 3H). ^13^C NMR (75 MHz, Chloroform-*d*) *δ* 173.3, 171.2, 159.2, 138.2, 128.8 (2C), 128.3, 125.3, 122.5, 122.2, 119.9, 114.3 (2C), 109.5, 101.8, 55.4, 42.9, 20.5.

#### 3.2.2. General Procedure of 3-Alkylidene-2-Indolones **10a**–**i**

Meldrum’s acid **7** (10 mmol) and DMAP (20 mmol) were added to CH_2_Cl_2_ (20 mL) and aryl chloride **6** (12 mmol) was added dropwise over 10 min at 0 °C. The mixture was then warmed to room temperature and stirred for 1 h. The reaction mixture was diluted with CH_2_Cl_2_ (20 mL) and washed sequentially with 10% HCl (20 mL) and then brine (30 mL). The combined organic layers were dried with Na_2_SO_4_ and concentrated. The residue was dissolved in acetonitrile (15 mL), aniline **1** (10 mmol) was added, and the mixture was heated to reflux for 1 h. Upon complete consumption of the starting materials, the solvent was removed under vacuum. The residue was purified by flash column chromatography to afford the corresponding intermediates **8**. The procedure for the subsequent diazo transfer reaction and preparation of target compounds **10** was similar to that used for the preparation of compounds **4** and **5.**

**(*Z*)-3-(hydroxy(thiophen-2-yl)methylene)-1-methylindolin-2-one** (**10a**) [28]. (DCM:MeOH = 150:1, R*_f_* = 0.38); Yield: 74%; purple crystals; mp: 141.0–143.0 °C; Lit. [24] mp: 99–101 °C.^1^H NMR (300 MHz, Chloroform-*d*) *δ* 14.34 (br. s, 1H), 7.90–7.87 (m, 1H), 7.73–7.64 (m, 2H), 7.25–7.20 (m, 2H), 7.04–6.95 (m, 2H), 3.40 (s, 3H). ^13^C NMR (75 MHz, Chloroform-*d*) *δ* 172.2, 164.2, 139.1, 136.6, 130.9, 130.9, 127.6, 126.2, 122.1, 121.5, 119.8, 108.6, 101.1, 26.1.
**(*Z*)-3-(hydroxy(phenyl)methylene)-1-methylindolin-2-one** (**10b**) [25]. (DCM:MeOH = 150:1, R*_f_* = 0.38); Yield: 83%; purple crystals; mp: 141.0–143.0 °C; Lit. [3] mp: 147–149 °C. ^1^H NMR (300 MHz, Chloroform-*d*) *δ* 14.18 (br. s, 1H), 7.84–7.75 (m, 2H), 7.62–7.50 (m, 3H), 7.24–7.15 (m, 2H), 7.00–6.86 (m, 2H), 3.41 (s, 3H). ^13^C NMR (75 MHz, Chloroform-*d*) *δ* 172.1, 171.1, 139.2, 134.3, 131.5, 128.8 (2C), 128.5 (2C), 126.0, 122.0, 121.7, 119.8, 108.5, 101.6, 26.0.
**(*Z*)-3-(hydroxy(p-tolyl)methylene)-1-methylindolin-2-one** (**10c**) [28]. (DCM:MeOH = 150:1, R*_f_* = 0.38); Yield: 84%; purple crystals; mp: 141.0–143.0 °C; Lit. [3] mp: 140–141 °C. ^1^H NMR (300 MHz, Chloroform-*d*) *δ* 13.93 (br. s, 1H), 7.70 (d, *J* = 8.2 Hz, 2H), 7.34 (d, *J* = 7.8 Hz, 2H), 7.28–7.16 (m, 2H), 6.99–6.87 (m, 2H), 3.40 (s, 3H), 2.47 (s, 3H). ^13^C NMR (75 MHz, Chloroform-*d*) *δ* 172.1, 171.5, 142.0, 139.1, 131.4, 129.4 (2C), 128.5 (2C), 125.8, 121.9, 121.9, 119.8, 108.4, 101.3, 26.0, 21.8.
**(*Z*)-3-((4-fluorophenyl)(hydroxy)methylene)-1-methylindolin-2-one** (**10d**) [28]. (DCM:MeOH = 150:1, R*_f_* = 0.38); Yield: 68%; purple crystals; mp: 141.0–143.0 °C; Lit. [3] mp: 96–97 °C ^1^H NMR (300 MHz, Chloroform-*d*) *δ* 14.12 (br. s, 1H), 7.91–7.83 (m, 2H), 7.33–7.26 (m, 3H), 7.26–7.21 (m, 1H), 7.04–6.95 (m, 2H), 3.46 (s, 3H). ^13^C NMR (75 MHz, Chloroform-*d*) *δ* 172.1, 169.9, 164.6 (d, *J* = 252.3 Hz), 139.3, 130.9 (d, *J* = 8.7 Hz, 2C), 130.4 (d, *J* = 3.5 Hz), 126.2, 122.1, 121.5, 119.6, 116.0 (d, *J* = 22.0 Hz, 2C), 108.6, 101.6, 26.0.
**(*Z*)-3-((4-chlorophenyl)(hydroxy)methylene)-1-methylindolin-2-one** (**10e**) [29]. (DCM:MeOH = 150:1, R*_f_* = 0.38); Yield: 74%; purple crystals; mp: 141.0–143.0 °C; ^1^H NMR (300 MHz, Chloroform-*d*) *δ* 14.03 (br. s, 1H), 7.74 (d, *J* = 8.4 Hz, 2H), 7.52 (d, *J* = 8.5 Hz, 2H), 7.25–7.15 (m, 2H), 6.98–6.89 (m, 2H), 3.40 (s, 3H). ^13^C NMR (75 MHz, Chloroform-*d*) *δ* 172.0, 169.6, 139.3, 137.6, 132.7, 130.0 (2C), 129.2 (2C), 126.3, 122.1, 121.4, 119.8, 108.7, 101.8, 26.0.
**(*Z*)-3-((4-chlorophenyl)(hydroxy)methylene)-1,7-dimethylindolin-2-one** (**10f**). (DCM:MeOH = 150:1, R*_f_* = 0.38); Yield: 71%; purple crystals; mp: 141.0–143.0 °C; ^1^H NMR (300 MHz, Chloroform-*d*) *δ* 14.33 (br. s, 1H), 7.73–7.67 (m, 2H), 7.53–7.47 (m, 2H), 7.02–6.96 (m, 1H), 6.95–6.90 (m, 1H), 6.81–6.74 (m, 1H), 3.67 (s, 3H), 2.63 (s, 3H). ^13^C NMR (75 MHz, Chloroform-*d*) *δ* 172.4, 169.6, 137.4, 137.3, 132.8, 130.0 (2C), 129.9, 129.1 (2C), 122.0, 121.9, 120.4, 117.8, 101.7, 29.4, 19.5; HRMS (APCI) *m/z* calcd for C_17_H_15_ClNO_2_^+^ 300.0786[M+H]^+^, found 300.0782[M+H]^+^.
**(*Z*)-7-bromo-3-((4-chlorophenyl)(hydroxy)methylene)-1-methylindolin-2-one** (**10g**). (DCM:MeOH = 150:1, R*_f_* = 0.38); Yield: 62%; purple crystals; mp: 141.0–143.0 °C; ^1^H NMR (300 MHz, Chloroform-*d*) *δ* 14.27 (br. s, 1H), 7.72–7.66 (m, 2H), 7.55–7.49 (m, 2H), 7.32–7.27 (m, 1H), 7.09–7.03 (m, 1H), 6.77–6.69 (m, 1H), 3.79 (s, 3H). ^13^C NMR (75 MHz, Chloroform-*d*) *δ* 172.4, 171.1, 137.9, 136.2, 132.3, 131.5, 130.0 (2C), 129.3 (2C), 124.5, 123.1, 118.6, 103.3, 101.0, 29.7; HRMS (APCI) *m/z* calcd for C_16_H_12_BrClNO_2_^+^ 363.9734[M+H]^+^, found 363.9730[M+H]^+^.
**(*Z*)-3-((4-chlorophenyl)(hydroxy)methylene)-1-propylindolin-2-one** (**10h**). (DCM:MeOH = 150:1, R*_f_* = 0.38); Yield: 80%; purple crystals; mp: 141.0–143.0 °C; ^1^H NMR (300 MHz, Chloroform-*d*) *δ* 14.08 (br. s, 1H), 7.79–7.70 (m, 2H), 7.56–7.49 (m, 2H), 7.23–7.15 (m, 2H), 7.01–6.87 (m, 2H), 3.93–3.81 (m, 2H), 1.80 (h, *J* = 7.4 Hz, 2H), 1.01 (t, *J* = 7.4 Hz, 3H). ^13^C NMR (75 MHz, Chloroform-*d*) *δ* 172.0, 169.8, 138.7, 137.5, 132.8, 130.0 (2C), 129.2 (2C), 126.2, 121.9, 121.6, 119.9, 109.0, 101.8, 41.6, 21.4, 11.6; HRMS (APCI) *m/z* calcd for C_18_H_17_ClNO_2_^+^ 314.0942[M+H]^+^, found 314.0940[M+H]^+^.
**(*Z*)-3-((4-bromophenyl)(hydroxy)methylene)-1-methylindolin-2-one** (**10i**). (DCM:MeOH = 150:1, R*_f_* = 0.38); Yield: 83%; purple crystals; mp: 141.0–143.0 °C; ^1^H NMR (300 MHz, Chloroform-*d*) *δ* 13.99 (br. s, 1H), 7.68 (s, 4H), 7.25–7.15 (m, 2H), 6.98–6.89 (m, 2H), 3.40 (s, 3H). ^13^C NMR (75 MHz, Chloroform-*d*) *δ* 172.0, 169.6, 139.4, 133.2, 132.1 (2C), 130.2 (2C), 126.3, 126.0, 122.1, 121.4, 119.8, 108.7, 101.8, 26.0; HRMS (APCI) *m/z* calcd for C_16_H_13_BrNO_2_^+^ 330.0124[M+H]^+^, found 330.0121[M+H]^+^.

### 3.3. In Vitro Biological Assays

#### 3.3.1. Antimicrobial Activity

This experiment used the double dilution method for determination. Test bacteria were grown to mid-log phase in tryptone soybean broth (TSB) and diluted 1000-fold in the same medium. Test compounds were prepared in DMSO, the final concentration of which did not exceed 0.05%. A two-fold serial dilution technique was used to obtain final concentrations of 64–0.5 μg/mL. The absorbance at 650 nm was read using a microplate reader before and after 24 h of culture, and the MIC values were calculated. All experiments were carried out three times. As positive controls, gatifloxacin and fluconazole were used. The seven strains used in this experiment are as follows: three Gram-positive bacteria, including *Staphylococcus aureus* (*S. aureus* 4220 and *S. aureus* ATCC 6538) and Methicillin-resistant *Staphylococcus aureus* (*MRSA* ATCC 43300); three Gram-negative bacteria, including *Escherichia coli* (*E. coli* 1924 and *E. coli* ATCC 25922), and *Pseudomonas aeruginosa* (*P. aeruginosa* CMCC (B) 10211); and one fungus, *Candida albicans* (*C. Albicans* CMCC(F) 98001).

The minimum bactericidal concentration (MBC) or minimum fungicidal concentration (MFC) was determined as described [31], following the MIC assay, by subculturing 5 μL of the compounds from wells showing no visible growth onto fresh agar plates. The plates were incubated at 37 °C for 24 h. The MBC or MFC was defined as the lowest concentration of the compound that resulted in a 99.9% reduction in the initial bacterial or fungal inoculum.

#### 3.3.2. Evaluation of Cytotoxicity

HepG2 cells and L-02 cells (ATCC, Manassas, VA, USA) were plated in 96-well plates at a density of 30–40% to ensure exponential growth throughout the experimental period, and then allowed to adhere for 1 day. Next, the cells were treated with eight concentrations (100, 50, 25, 10, 5, 1, 0.1, and 0.01 μmol/L) of each compound. After 48 h of incubation, MTT solution was added to each well at a final concentration of 2 mg/mL and incubated for an additional 4 h. Next, the MTT solution was removed and 150 mL of dimethyl sulphoxide (DMSO)/well was added (Sigma-Aldrich, St. Louis, MO, USA). The plates were shaken vigorously at RT to ensure complete solubilization, and absorption at 492 nm was determined by a microplate reader (BioTek Instruments, Inc., Winooski, VT, USA). At least three independent experiments were conducted.

#### 3.3.3. Molecular Docking

The molecular computation study was carried out using Discovery Studio 2017 (Accelry, San Diego, CA, USA). The 3D structure of DHFR (PDB ID: 1DLS) was obtained from a protein data bank. The water molecule and complexed ligand in the protein were removed and the proteins were prepared by adding hydrogen and correcting incomplete residues using a clean protein tool of DS. The proteins were then refined with CHARMm. The structures of **5a**, **5v**, **10h** and gatifloxacin were sketched in 2D and converted into 3D using the DS molecule editor. Automated docking studies were carried out to investigate the binding mode of compounds **5a**, **5v**, and **10h** in the crystal structure utilizing DS CDocker protocol.

## 4. Conclusions

In this report, two series of 3-alkylidene-2-indolone derivatives (**5a**–**w** and **10a**–**i**) were synthesized and their antimicrobial activities were evaluated in vitro. Among the 32 synthesized target compounds, 25 compounds demonstrated moderate to high inhibitory effects on at least one of the Gram-positive bacteria, Gram-negative bacteria, or fungus. The compounds **10f**, **10g** and **10h** exhibited the strongest antibacterial activity, with a MIC of 0.5 μg/mL, matching the activity of the positive control compound gatifloxacin in inhibiting three strains of Gram-positive bacteria: *Staphylococcus aureus* ATCC 6538 and 4220, and Methicillin-resistant *Staphylococcus aureus* ATCC 43300 separately. Four compounds, **5d**, **5h**, **5r** and **10d** showed moderate antifungal activity against the fungus CMCC 98001. Compounds **10f** and **10g** did not exhibit toxicity against HepG2 cells and L-02 cells while compound **10h** showed moderate cytotoxicity. Overall, the compounds **10f**, **10g** and **10h** are promising candidates for the development of drugs to treat bacterial infections.

## Figures and Tables

**Table 1:** Inhibitory activity of compounds **5a**–**w** and **10a**–**i** against various bacteria and fungus (MIC, μg/mL) ^a^.

	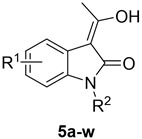			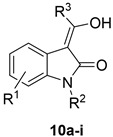			
Comp.	Gram-Positive Bacteria			Gram-Negative Bacteria			Fungus
	4220 ^b^	6538 ^c^	43300 ^d^	10211 ^e^	25922 ^f^	1924 ^g^	98001 ^h^
**5a**	64	>64	>64	64	>64	64	32
**5b**	>64	64	>64	64	64	32	32
**5c**	>64	64	>64	64	64	16	16
**5d**	16	64	>64	64	>64	2	32
**5e**	4	64	64	>64	>64	16	32
**5f**	>64	>64	>64	>64	>64	32	64
**5g**	>64	64	>64	>64	>64	64	32
**5h**	64	>64	>64	>64	>64	32	16
**5i**	32	32	64	64	>64	8	64
**5j**	32	16	>64	>64	>64	8	64
**5k**	4	16	>64	>64	>64	1	32
**5l**	32	64	64	>64	>64	8	64
**5m**	>64	32	64	>64	>64	16	64
**5n**	32	32	>64	>64	>64	8	64
**5o**	>64	>64	64	64	>64	16	32
**5p**	64	64	>64	>64	>64	>64	>64
**5q**	64	16	>64	>64	>64	16	>64
**5r**	64	8	8	>64	>64	>64	16
**5s**	32	64	64	>64	>64	16	>64
**5t**	>64	64	>64	>64	>64	32	32
**5u**	8	16	64	>64	64	2	32
**5v**	64	64	16	>64	>64	16	32
**5w**	>64	64	64	>64	>64	8	32
**10a**	>64	64	64	64	>64	32	64
**10b**	32	8	32	>64	64	8	>64
**10c**	8	2	16	>64	64	8	64
**10d**	16	4	32	>64	64	8	8
**10e**	8	2	16	>64	64	8	64
**10f**	4	0.5	2	>64	>64	8	>64
**10g**	0.5	2	0.5	>64	64	1	>64
**10h**	1	0.5	0.5	16	16	1	64
**10i**	16	2	16	>64	>64	8	>64
Gati ^i^	0.5	0.5	0.5	0.5	0.5	0.5	>64
Flu ^j^	>64	>64	>64	>64	>64	>64	2

^a^ Not active (64 μg/mL < MIC); weak activity (32 μg/mL ≤ MIC ≤ 64 μg/mL); moderate activity (8 μg/mL ≤ MIC ≤ 16 μg/mL); high activity (MIC ≤ 4 μg/mL); ^b^ *S. aureus* 4220; ^c^ *S. aureus* ATCC 6538; ^d^ *MRSA* ATCC 43300; ^e^ *P. aeruginosa* CMCC 10211; ^f^ *E. coli* ATCC 25922; ^g^ *E. coli* 1924; ^h^ *C. albicans* CMCC 98001; ^i^ Gatifloxacin; ^j^ Fluconazole.

**Table 2:** MBC or MFC results for the most potent compounds **10d**, **10f**, **10g** and **10h** against microbial strains.

Comp.	Microbial Strains	MIC [μg/mL]	MBC [μg/mL]	MFC [μg/mL]	MBC/MIC	MFC/MIC
**10f**	*Staphylococcus aureus* ATCC 6538	0.5	32	-	64	-
**10g**	*Staphylococcus aureus* 4220	0.5	32	-	64	-
**10h**	*Staphylococcus aureus* ATCC 43300	0.5	32	-	64	-
**10d**	*Candida albicans* CMCC 98001	8	-	16	-	2

**Table 3:** Cytotoxicity levels of selected compounds in HepG2 cell line and L-02 cell line.

Comp.	HepG2	L-02
	IC_50_/µM	
**10f**	>100	>100
**10g**	>100	>100
**10h**	41.6	53.5

## Data Availability

Data are contained within the article and Supplementary Materials.

## Supplementary Materials

The following supporting information can be downloaded at: https://www.mdpi.com/article/10.3390/molecules29225384/s1, NMR, HRMS spectra.
