# Supplementary material for: Synthesis and Antimicrobial Activity of 3-Alkylidene-2-Indolone Derivatives
Source: Molecules. 2024 Nov 15;29(22):5384. doi: 10.3390/molecules29225384 (PMC11596972; doi:10.3390/molecules29225384)
Supplement: Supplementary file 1 [file molecules-29-05384-s001.zip › molecules-3261505-supplementary.pdf]

Supporting Information for

Synthesis and Antimicrobial Activity of 3-Alkylidene-2-Indolone Derivatives

List of Contents

^{1}H, ^{13}C NMR and Mass-Spectra

Compounds 5a-w and 10a-i

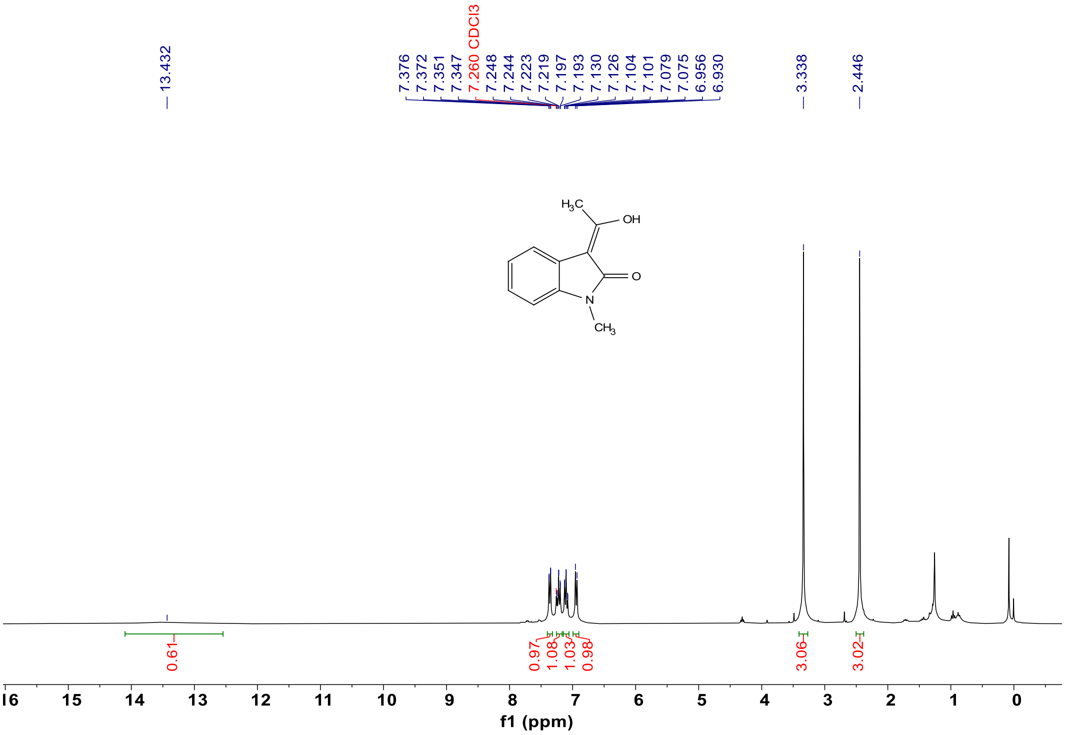

^{1}H NMR spectrum of compound **5a** (300 MHz, $CDCl_3$)

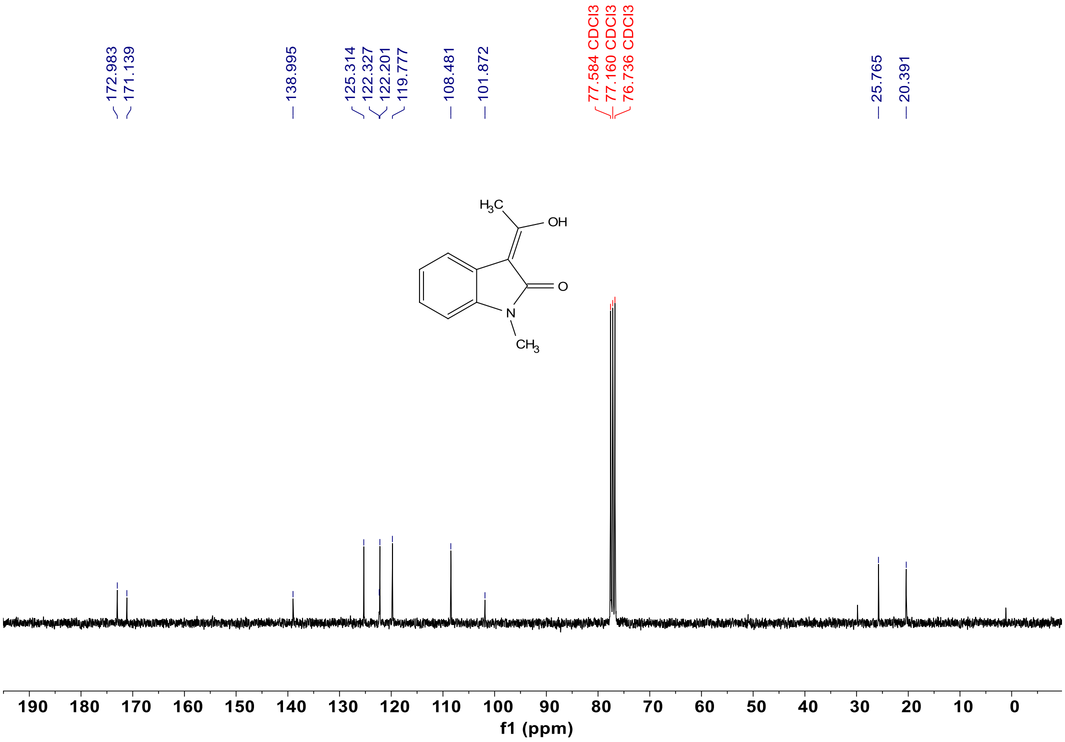

^{13}C NMR spectrum of compound **5a** (75 MHz, $CDCl_3$)

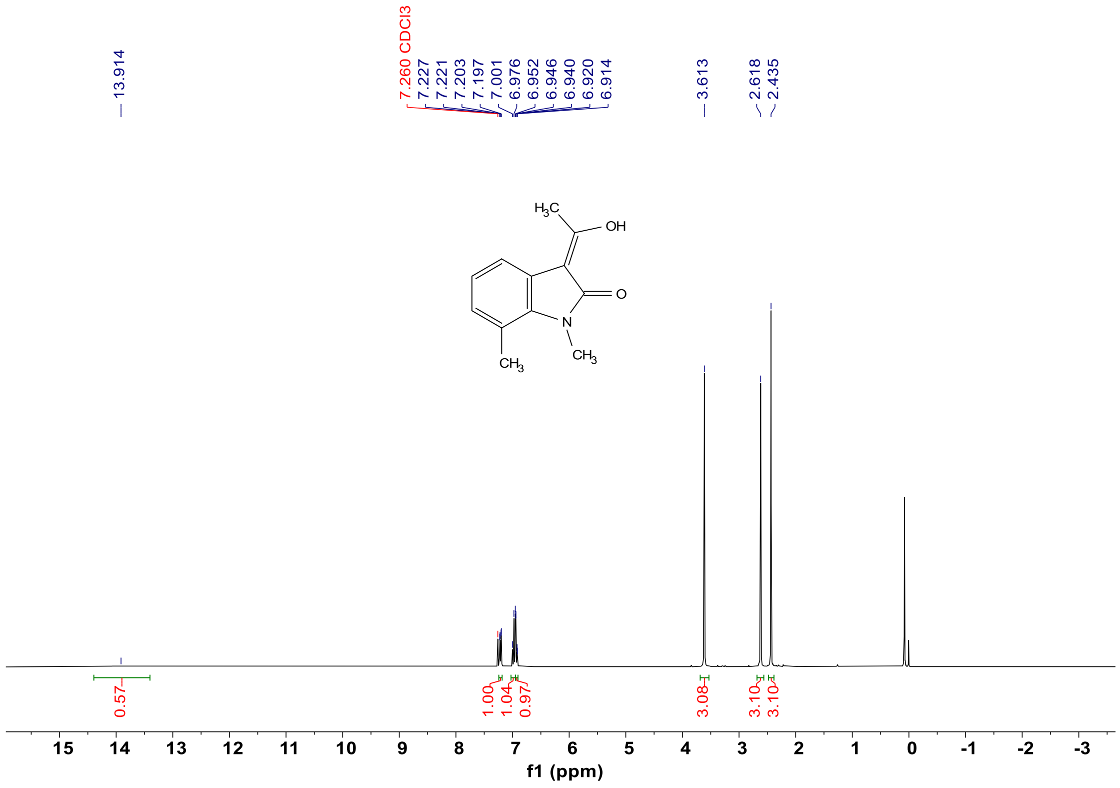

^{1}H NMR spectrum of compound **5b** (300 MHz, $CDCl_3$)

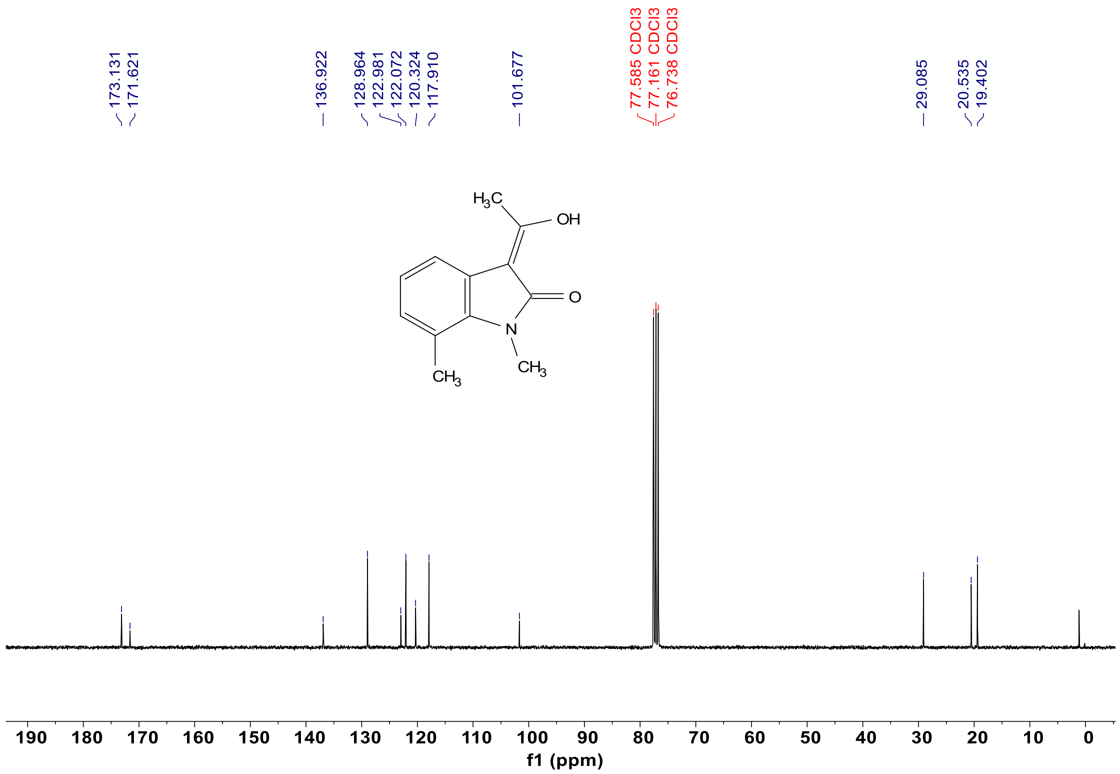

^{13}C NMR spectrum of compound **5b** (75 MHz, $CDCl_3$)

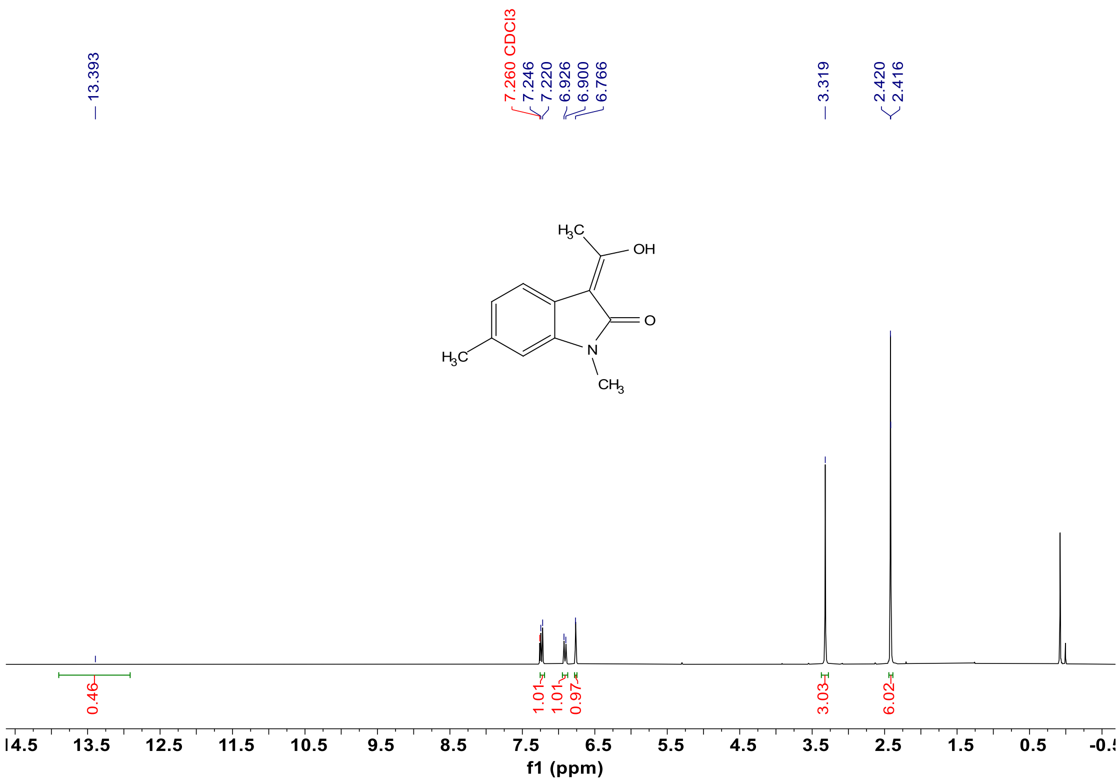

^{1}H NMR spectrum of compound **5c** (300 MHz, $CDCl_3$)

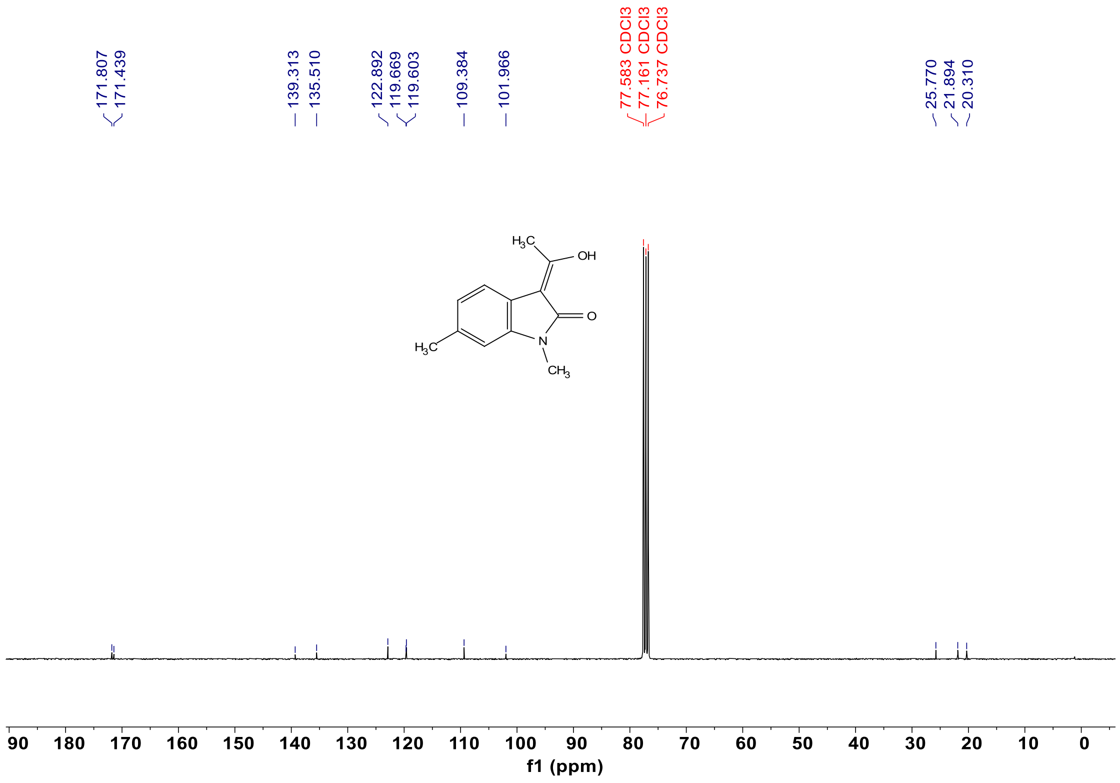

^{13}C NMR spectrum of compound **5c** (75 MHz, $CDCl_3$)

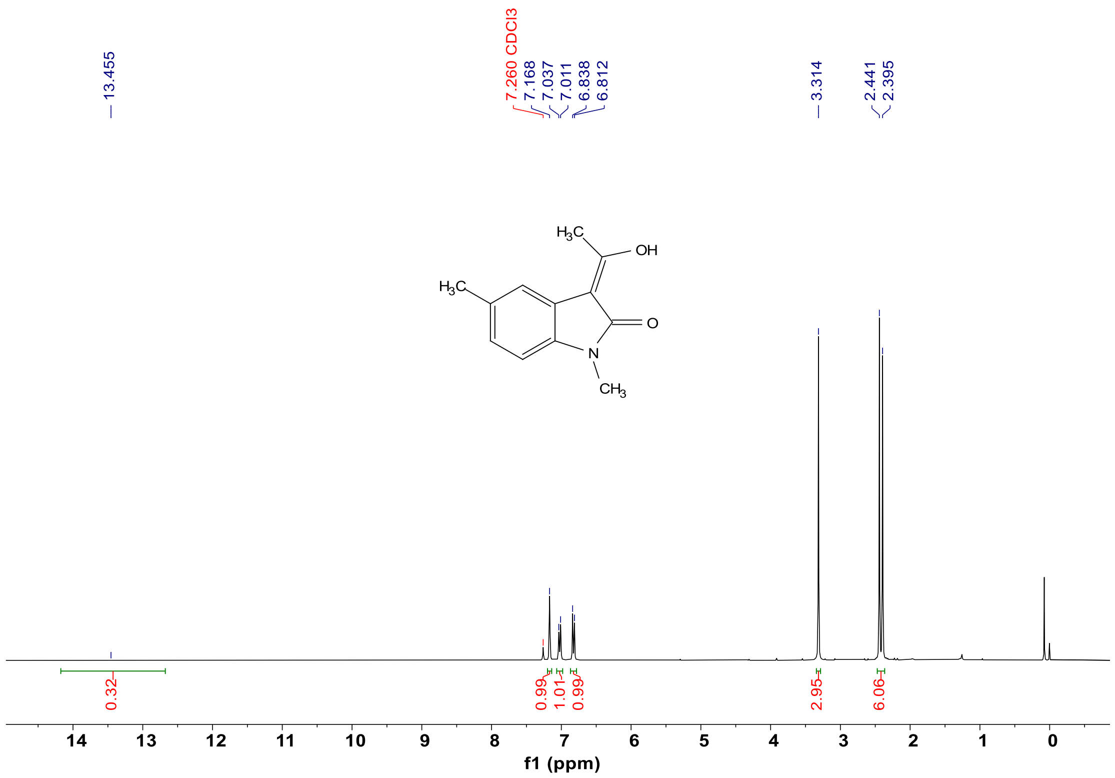

^{1}H NMR spectrum of compound **5d** (300 MHz, $CDCl_3$)

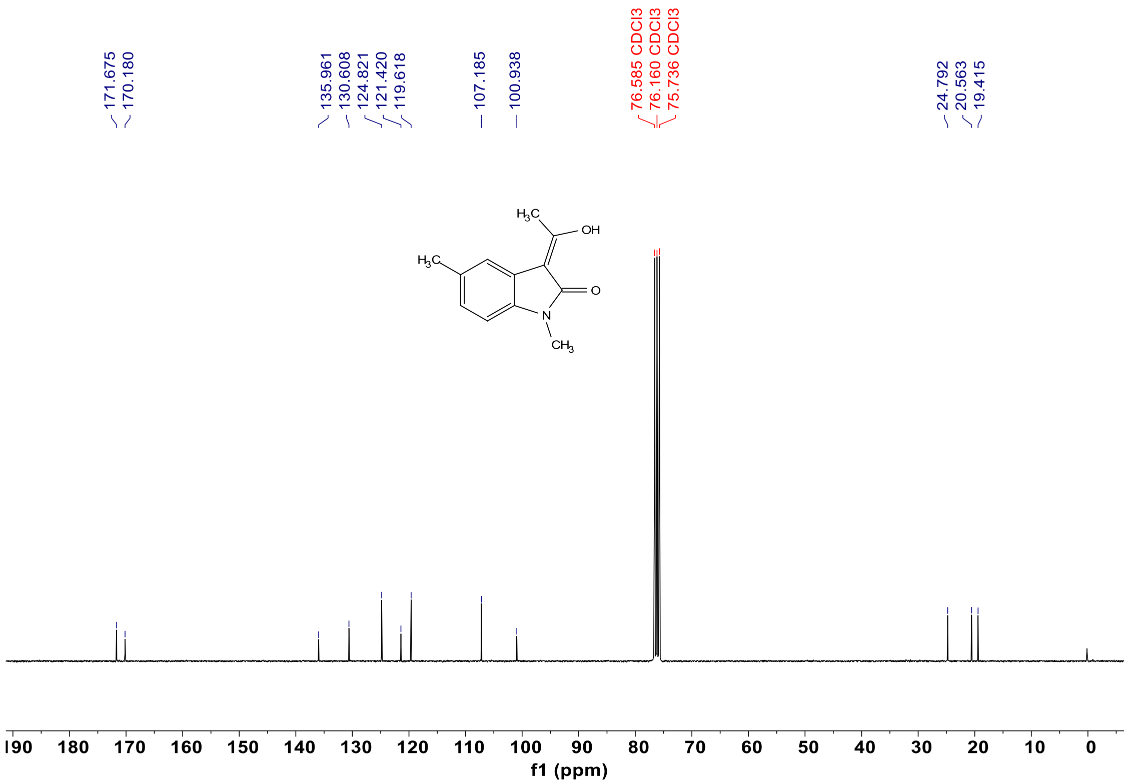

^{13}C NMR spectrum of compound **5d** (75 MHz, $CDCl_3$)

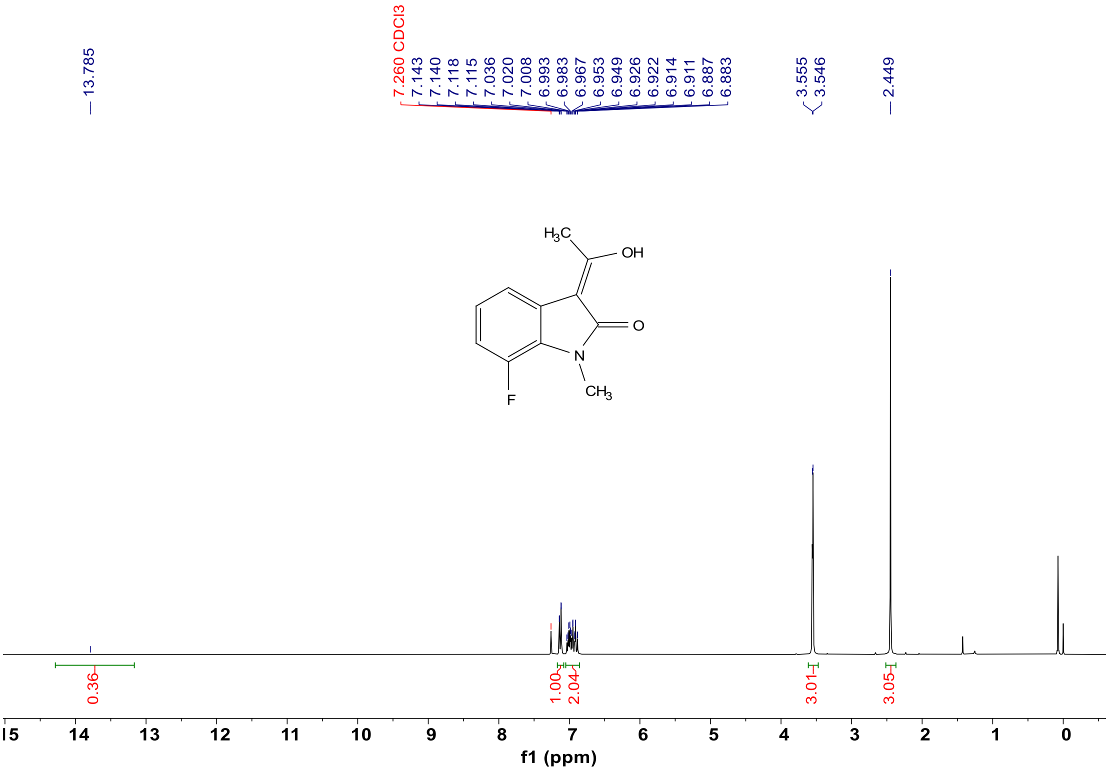

^{1}H NMR spectrum of compound **5e** (300 MHz, $CDCl_3$)

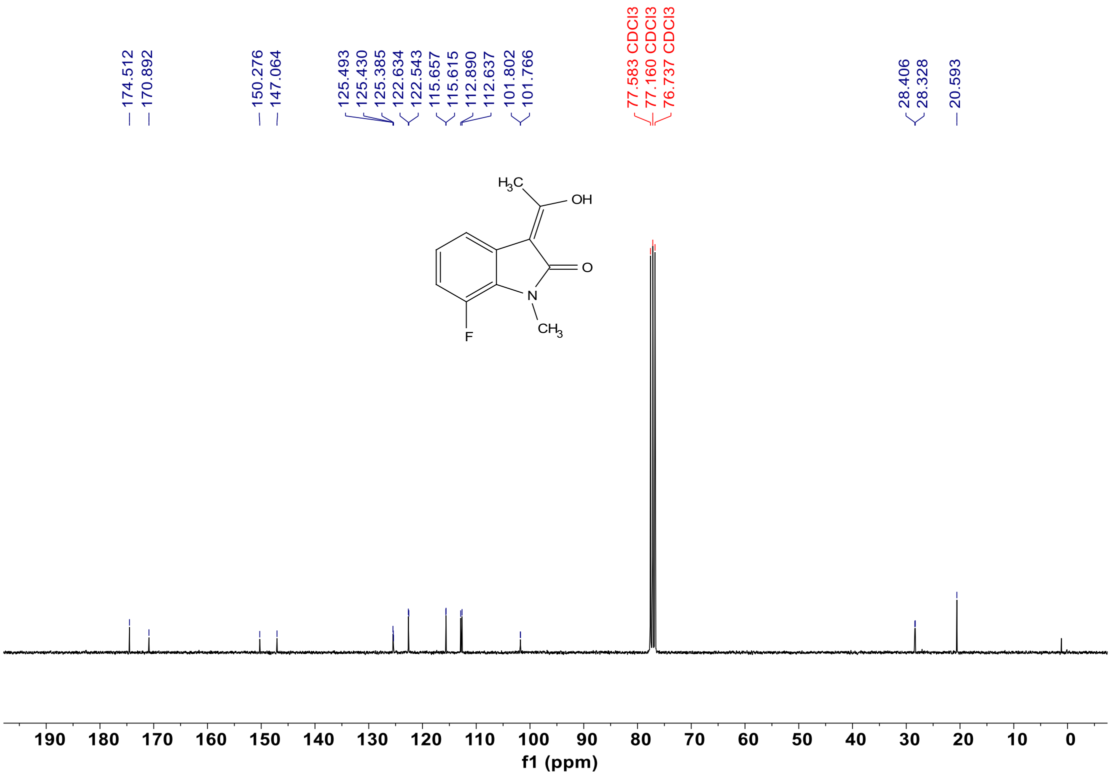

^{13}C NMR spectrum of compound **2-5e** (75 MHz, $CDCl_3$)

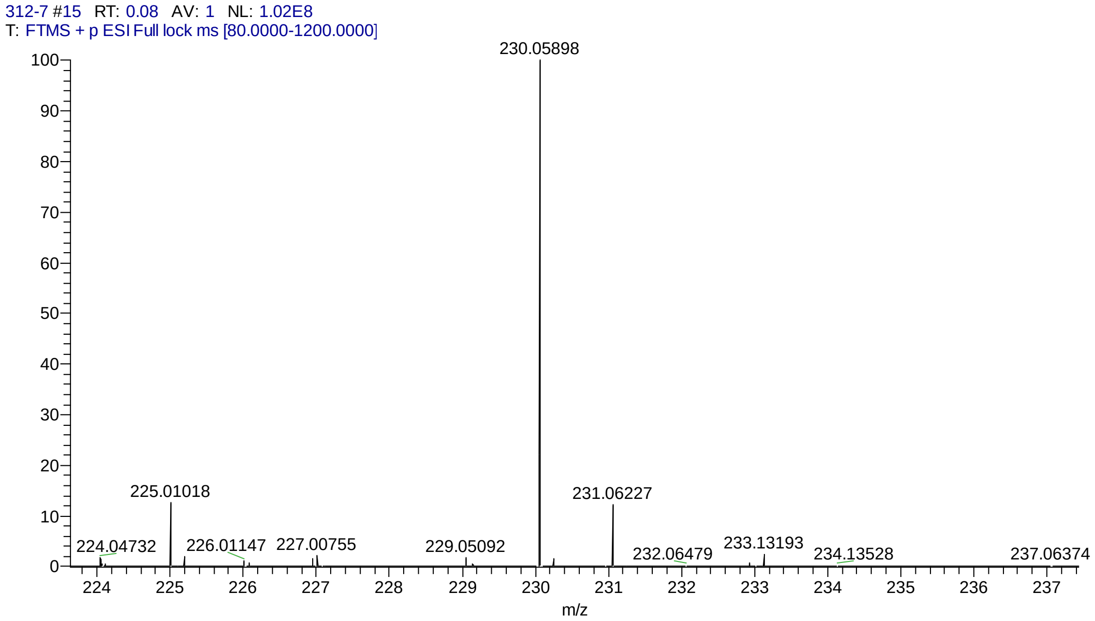

HRMS(ESI) of compound **5e**

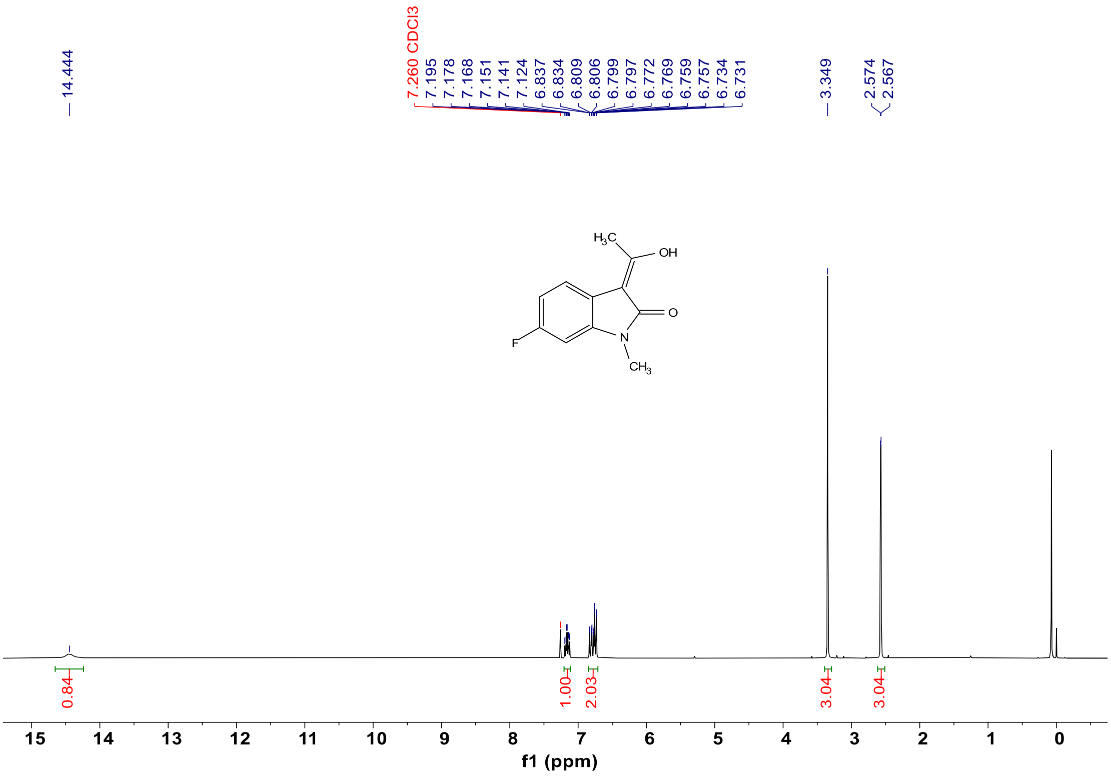

^{1}H NMR spectrum of compound **5f** (300 MHz, $CDCl_3$)

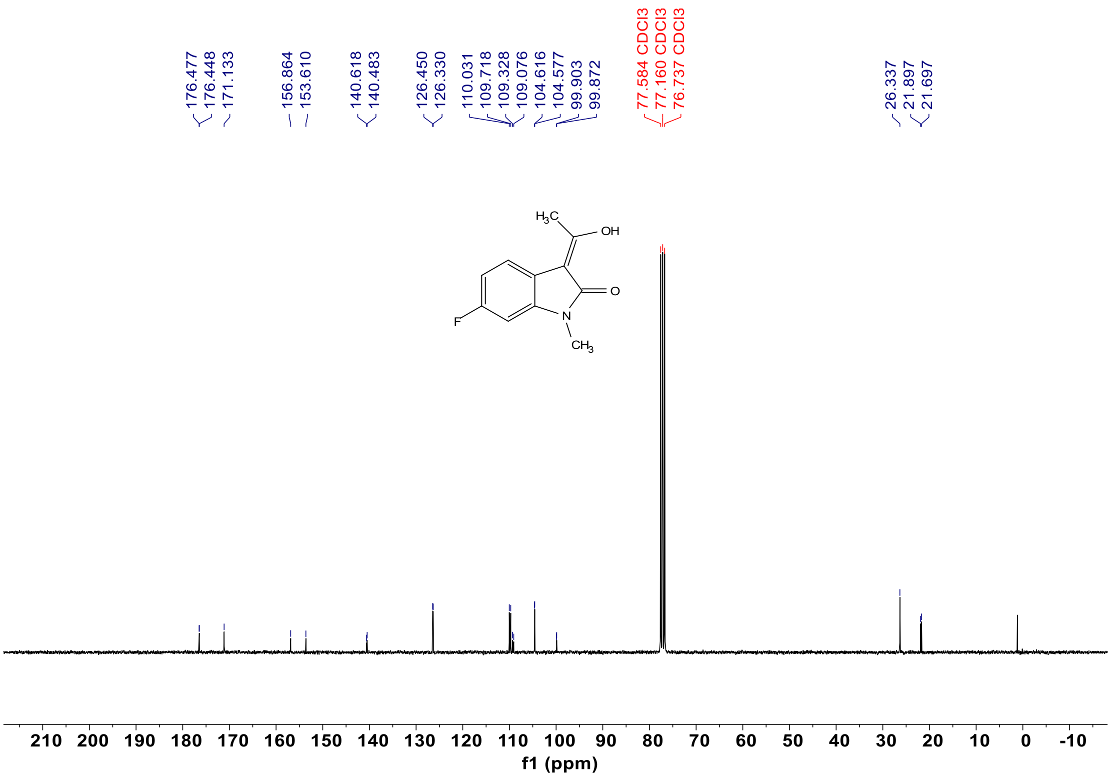

^{13}C NMR spectrum of compound **5f** (75 MHz, $CDCl_3$)

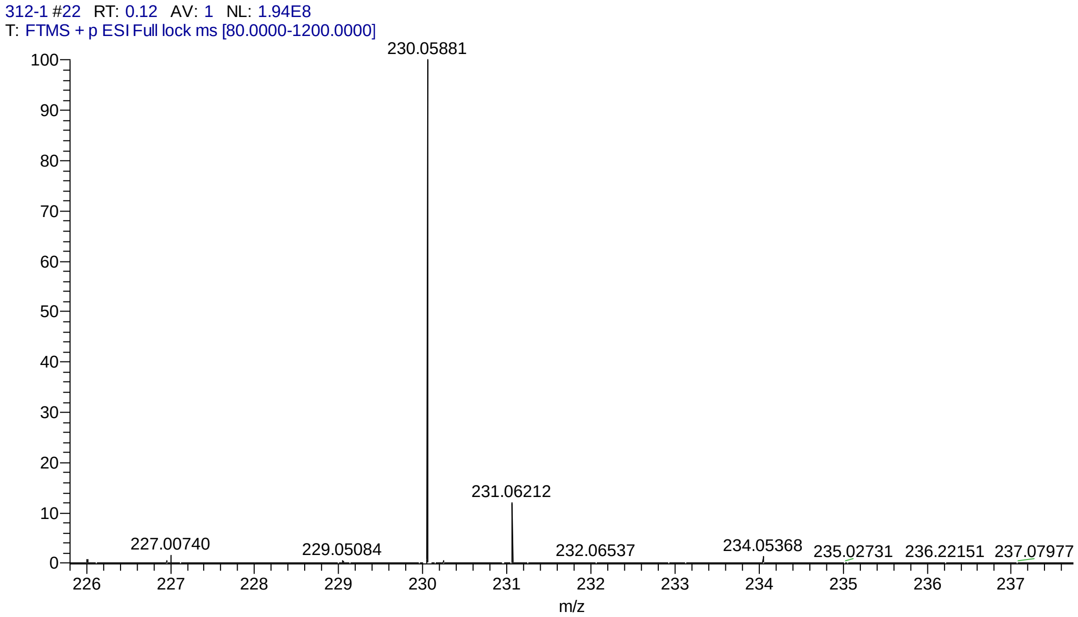

HRMS(ESI) of compound **5f**

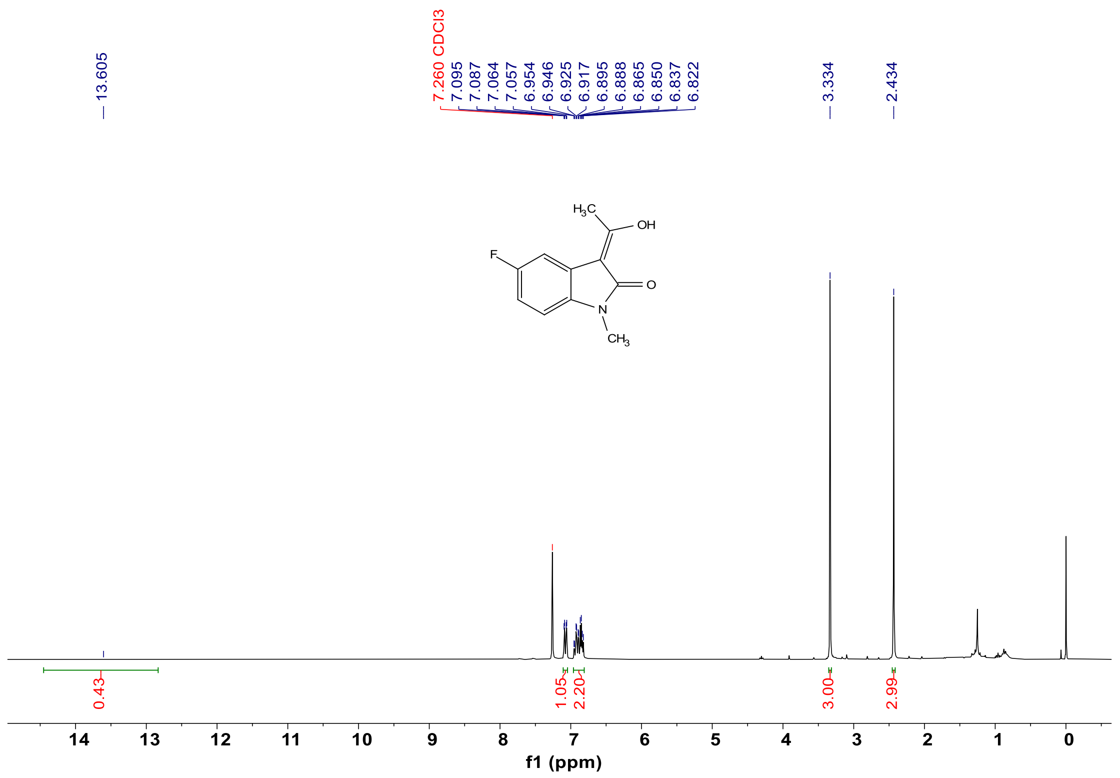

^{1}H NMR spectrum of compound **5g** (300 MHz, $CDCl_3$)

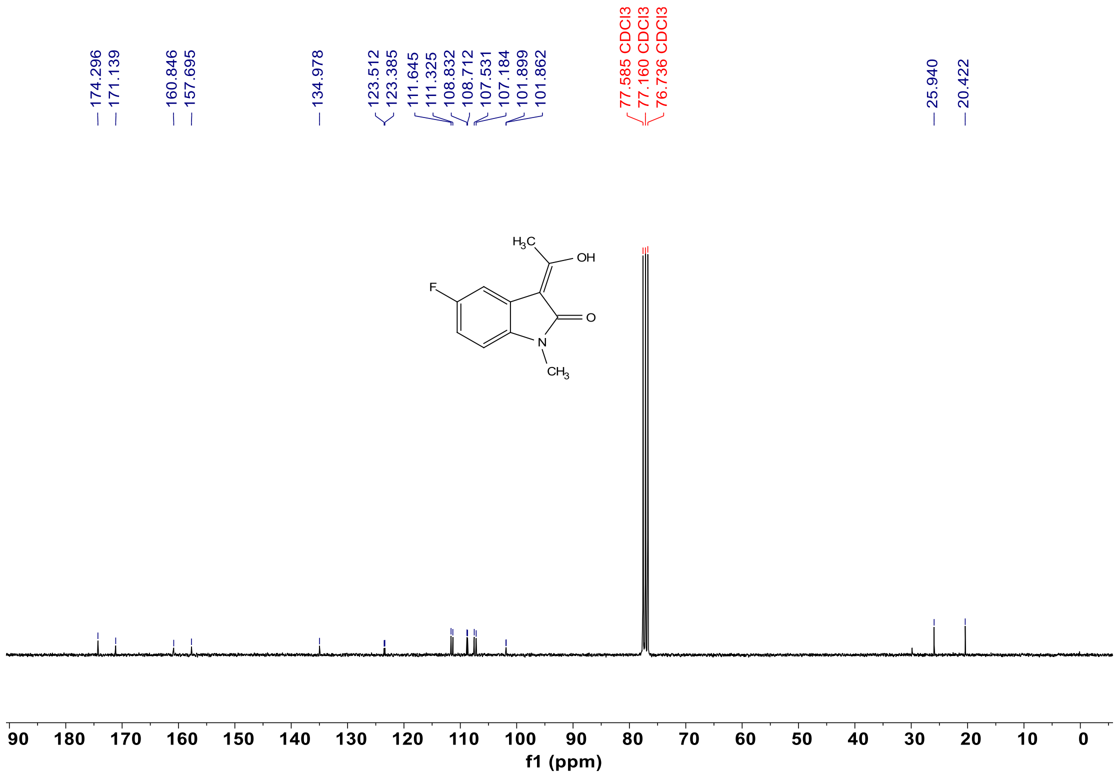

^{13}C NMR spectrum of compound **5g** (75 MHz, $CDCl_3$)

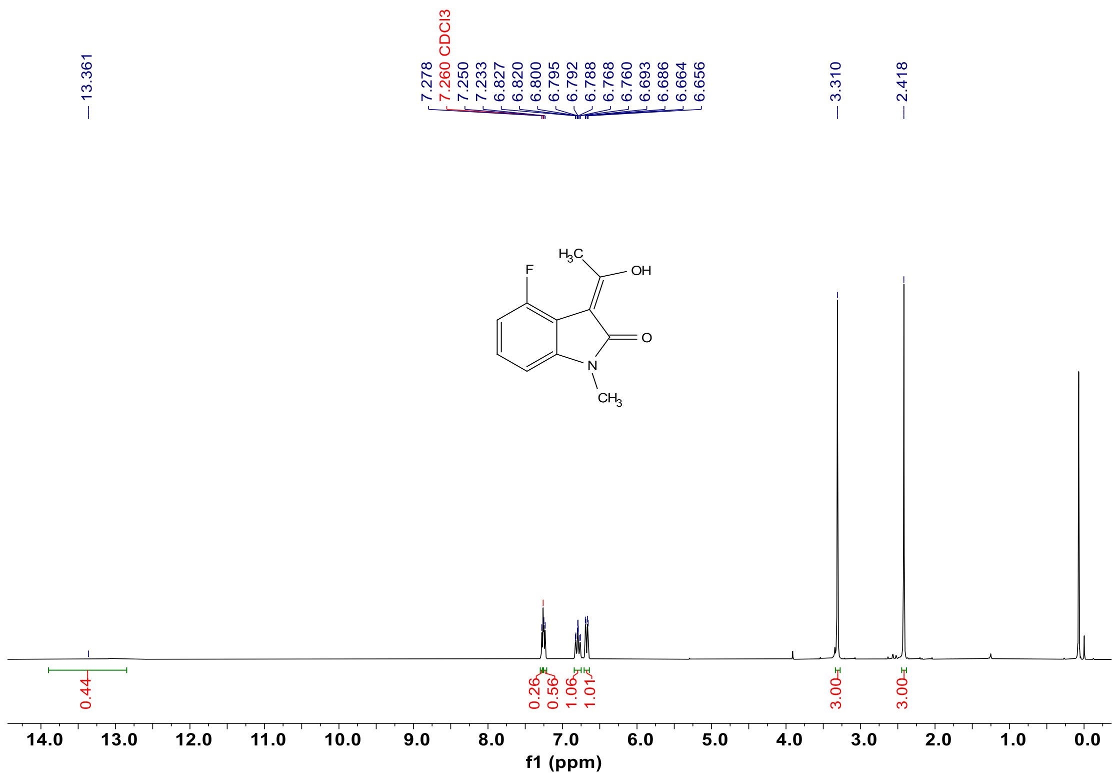

^{1}H NMR spectrum of compound **5h** (300 MHz, $CDCl_3$)

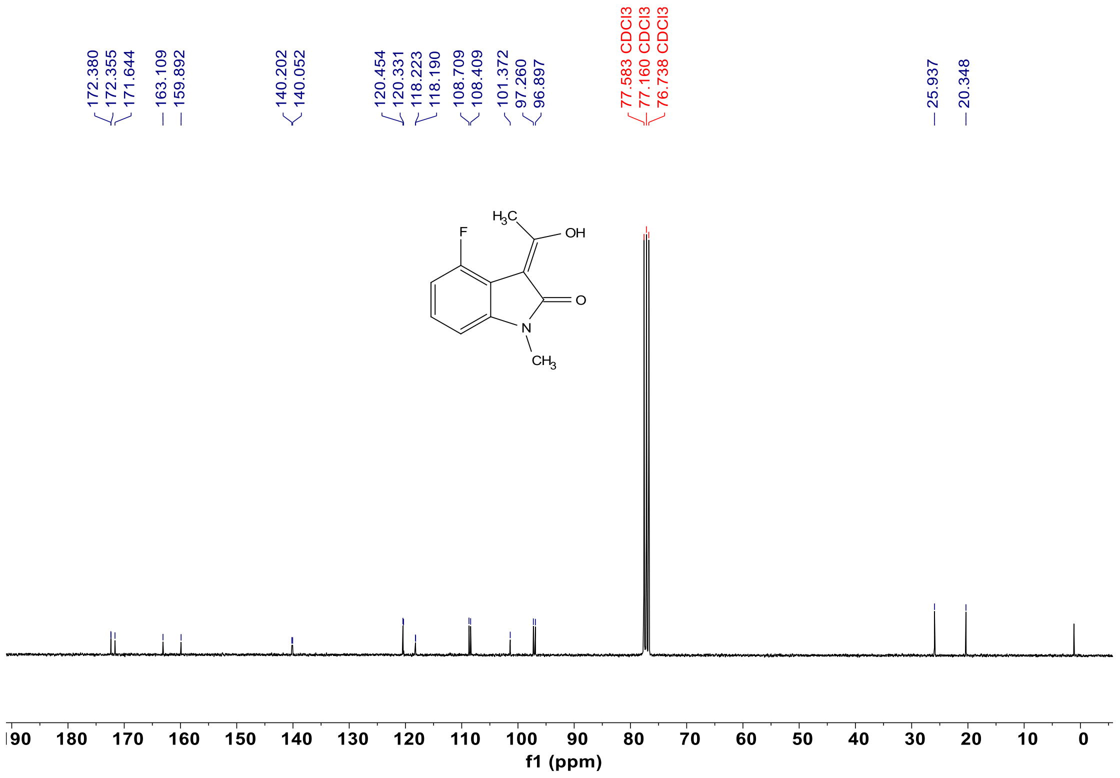

^{13}C NMR spectrum of compound **5h** (75 MHz, $CDCl_3$)

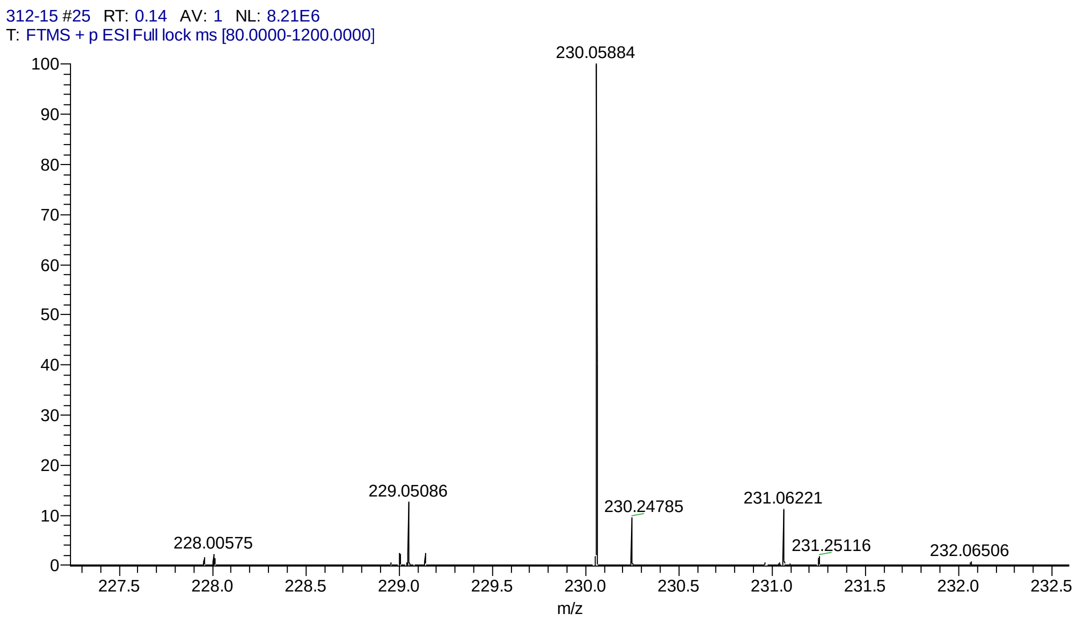

HRMS(ESI) of compound **5h**

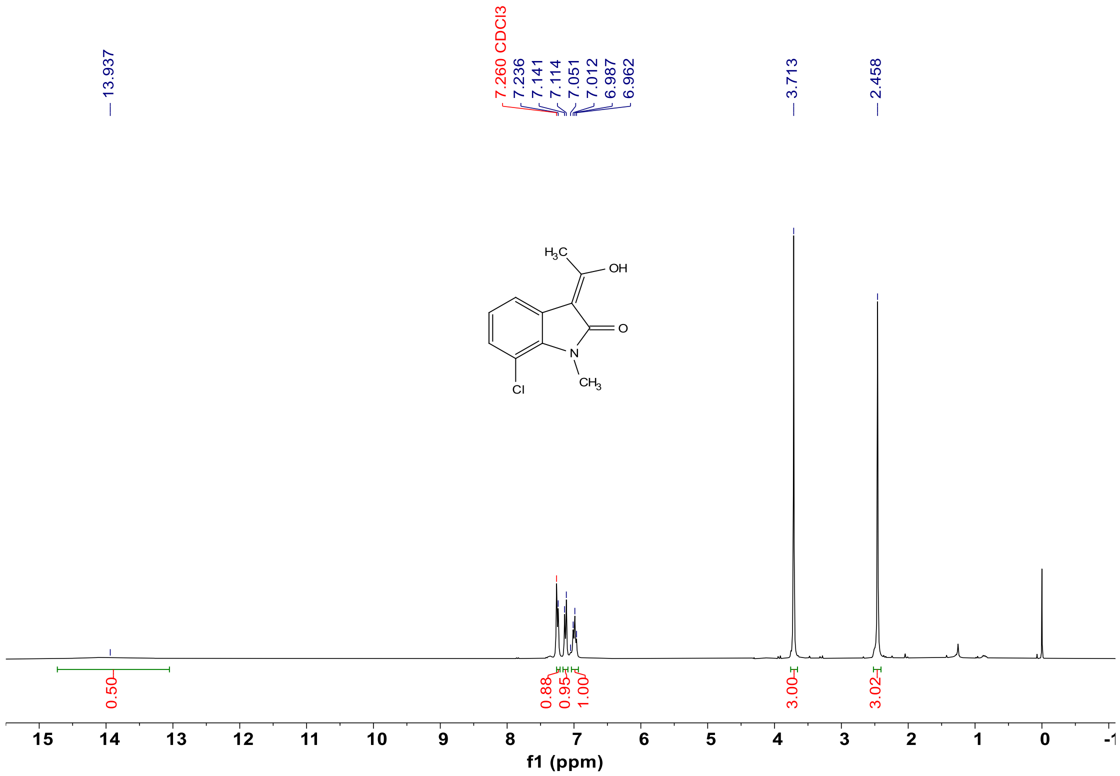

^{1}H NMR spectrum of compound **5i** (300 MHz, $CDCl_3$)

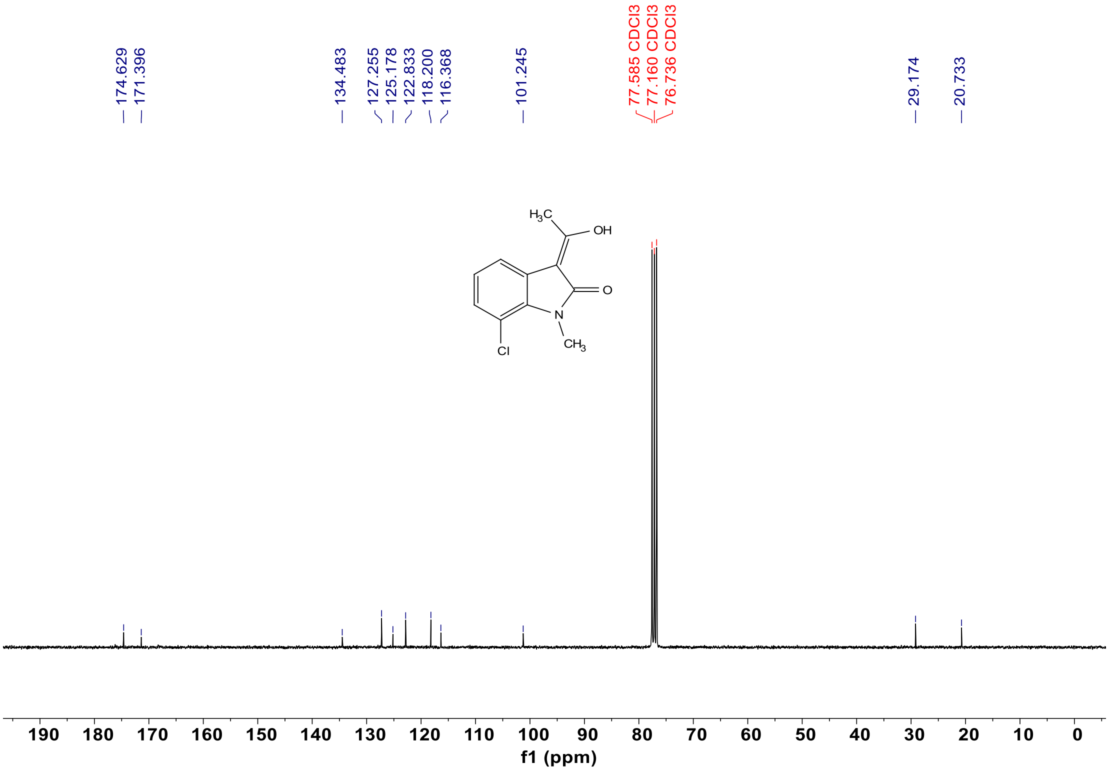

^{13}C NMR spectrum of compound **5i** (75 MHz, $CDCl_3$)

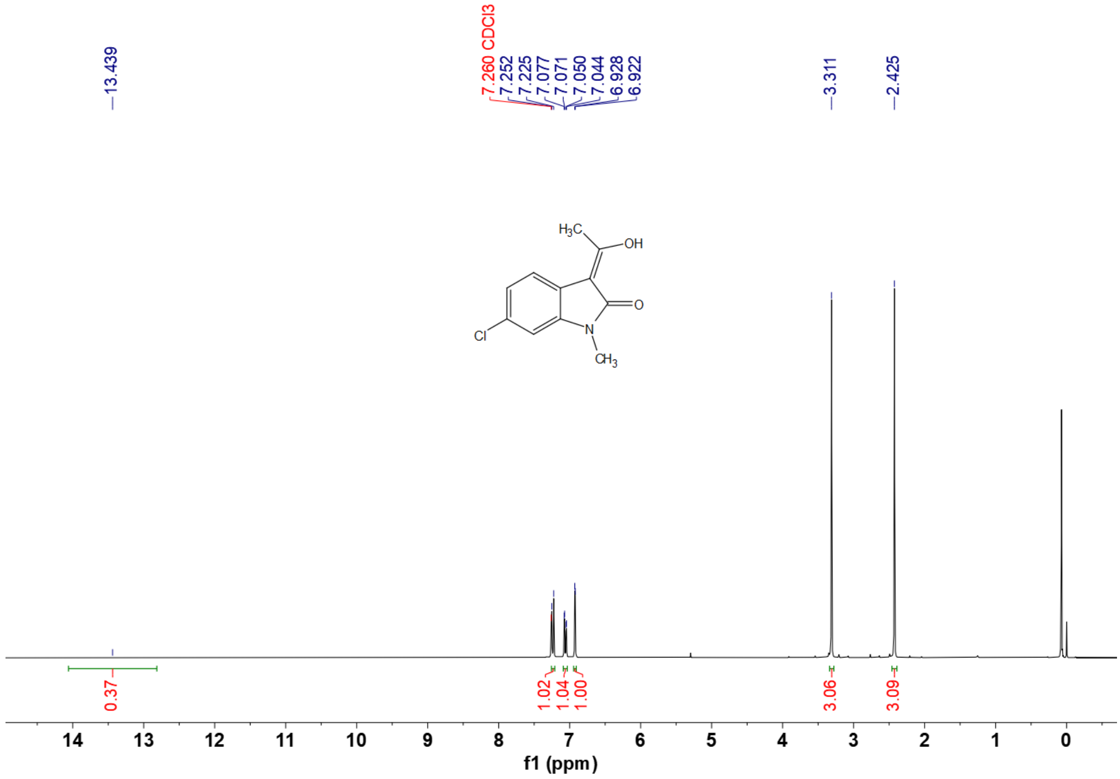

^{1}H NMR spectrum of compound **5j** (300 MHz, $CDCl_3$)

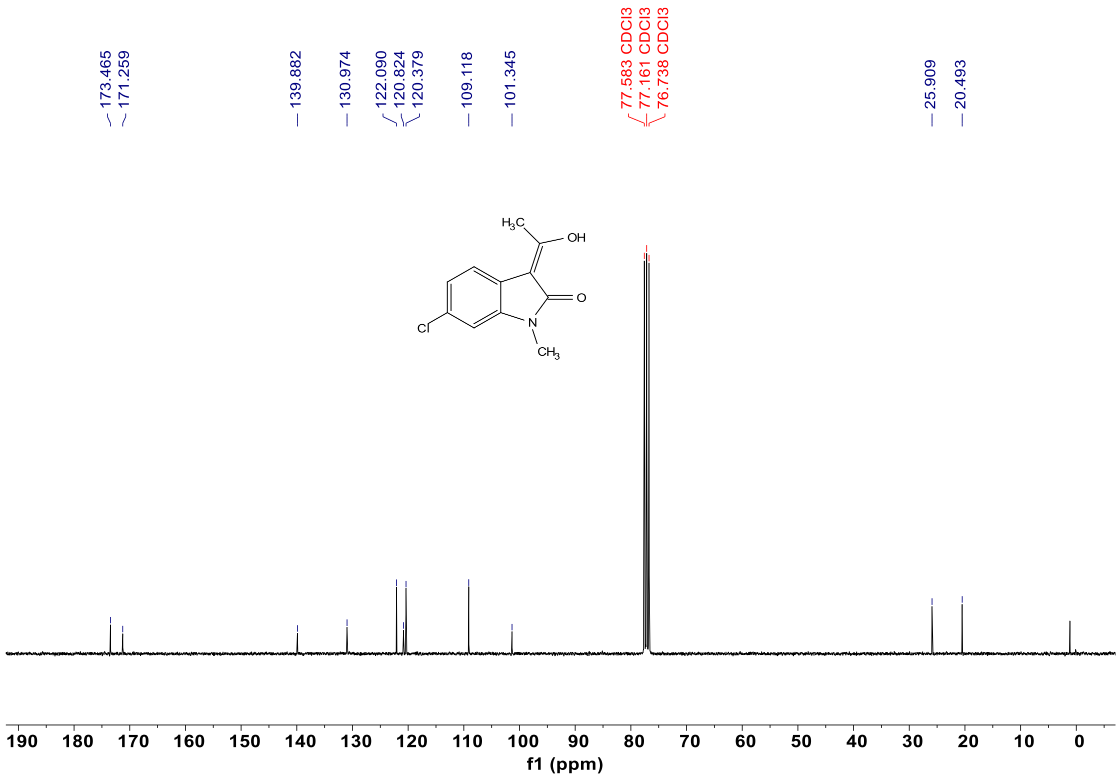

^{13}C NMR spectrum of compound **5j** (75 MHz, $CDCl_3$)

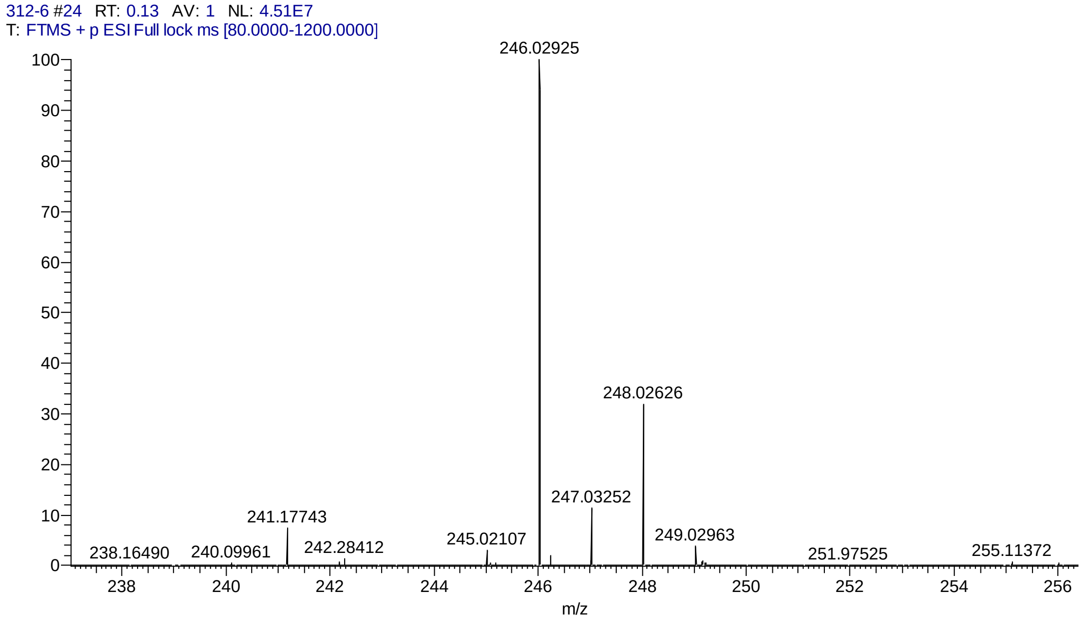

HRMS(ESI) of compound **5j**

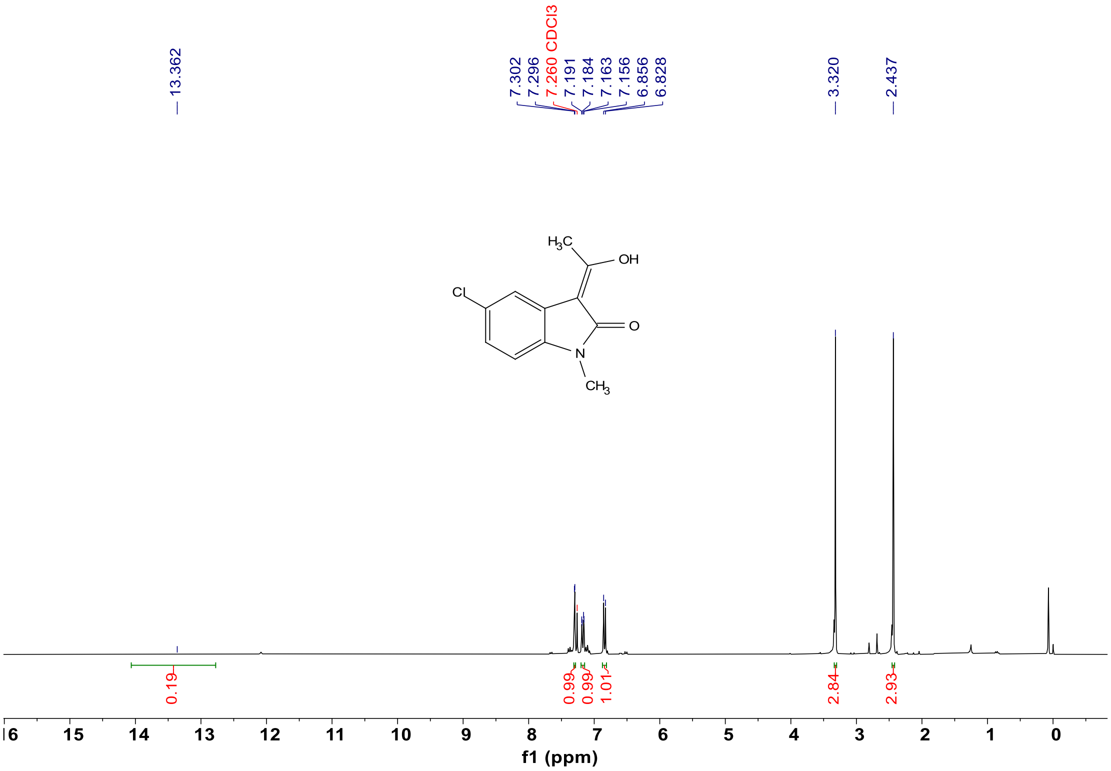

^{1}H NMR spectrum of compound **5k** (300 MHz, $CDCl_3$)

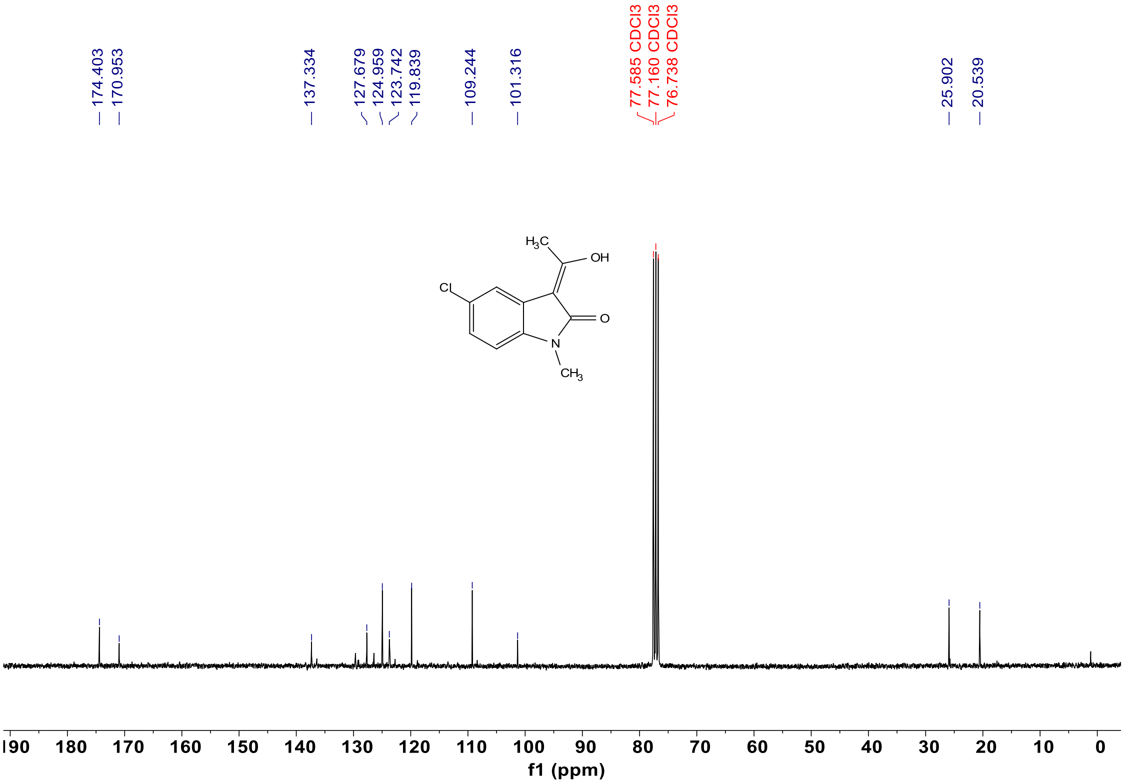

^{13}C NMR spectrum of compound **5k** (75 MHz, $CDCl_3$)

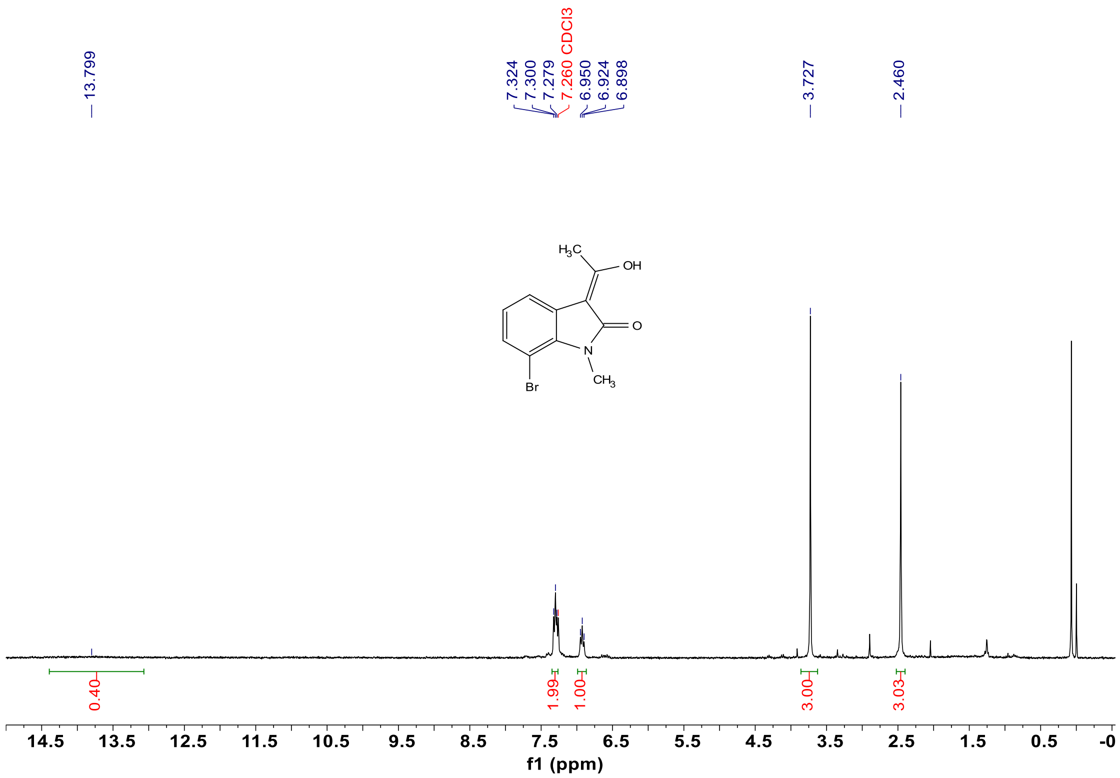

^{1}H NMR spectrum of compound **5l** (300 MHz, $CDCl_3$)

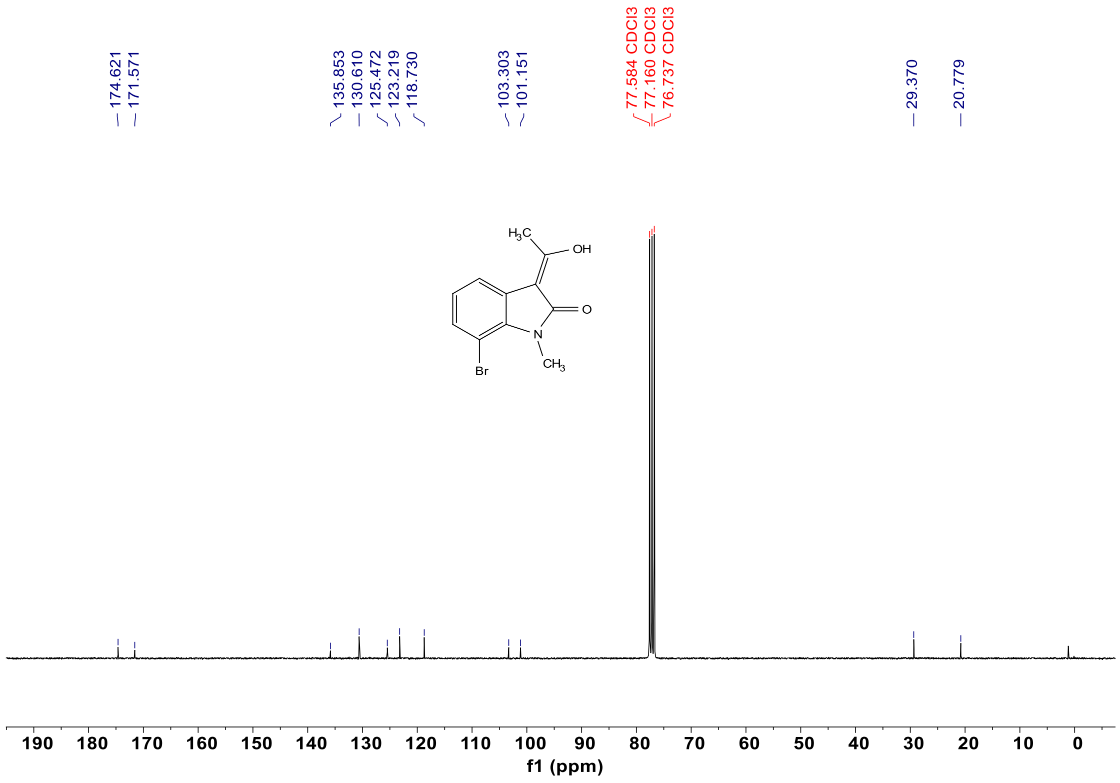

^{13}C NMR spectrum of compound **5l** (75 MHz, $CDCl_3$)

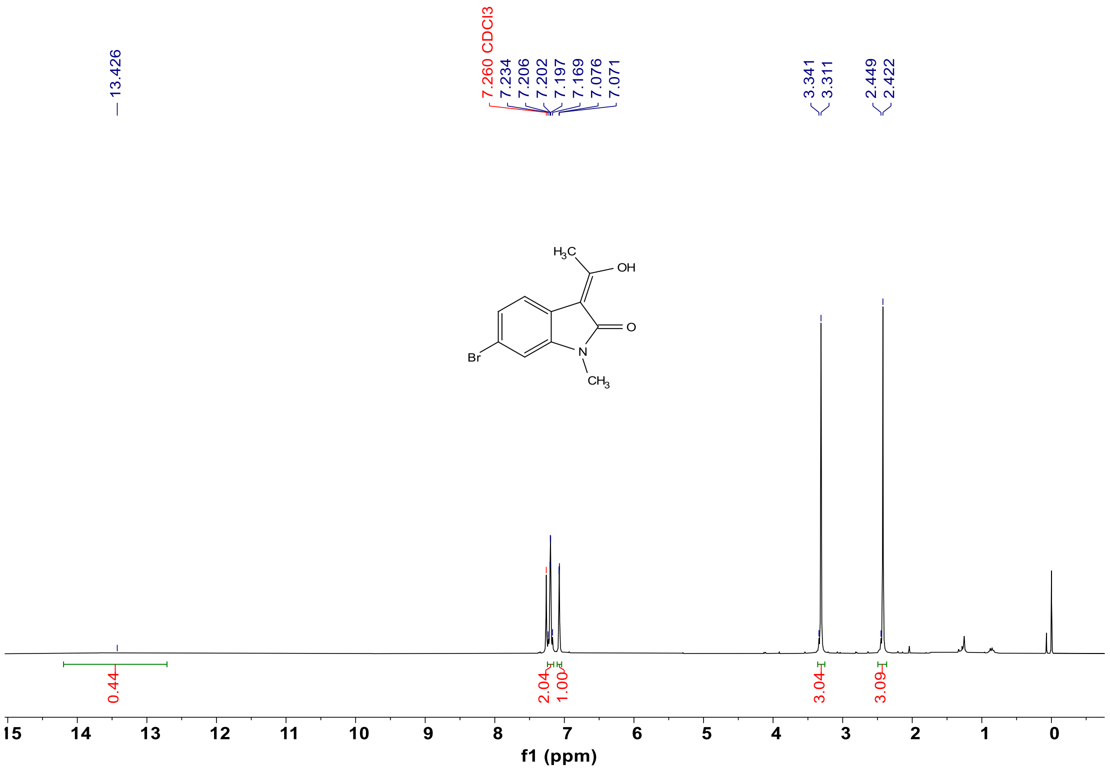

^{1}H NMR spectrum of compound **5m** (300 MHz, $CDCl_3$)

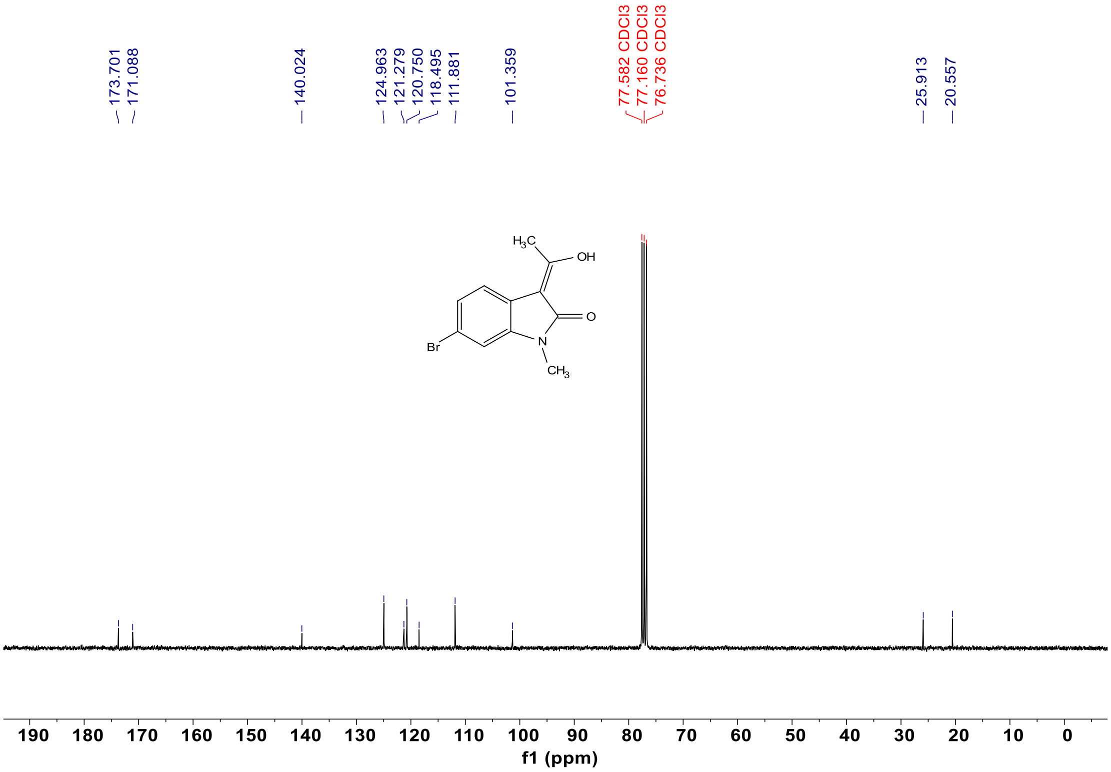

^{13}C NMR spectrum of compound **5m** (75 MHz, $CDCl_3$)

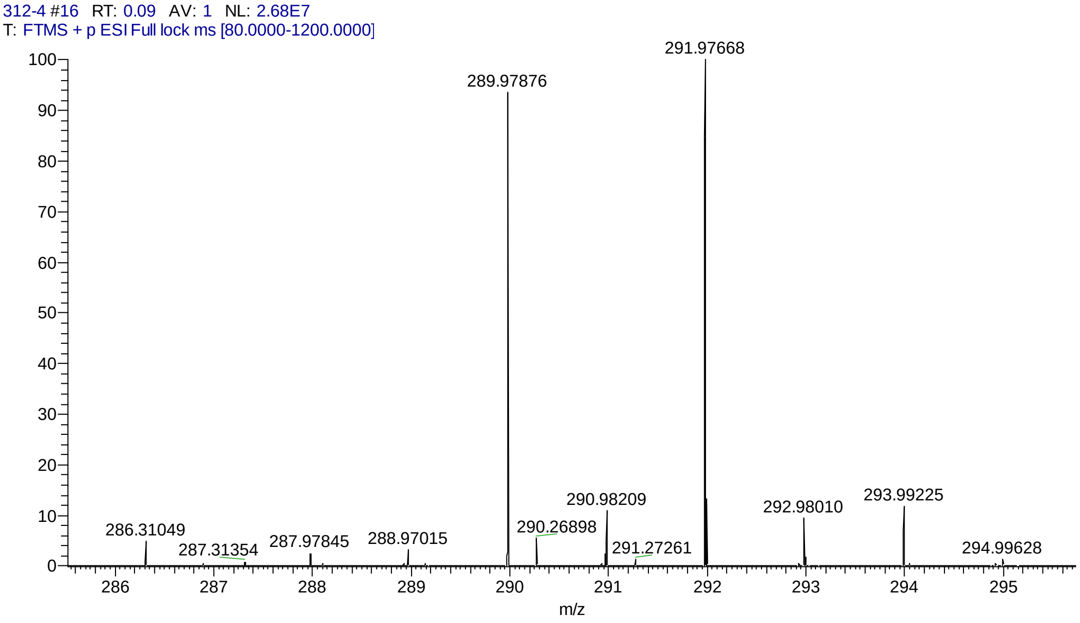

HRMS(ESI) of compound **5m**

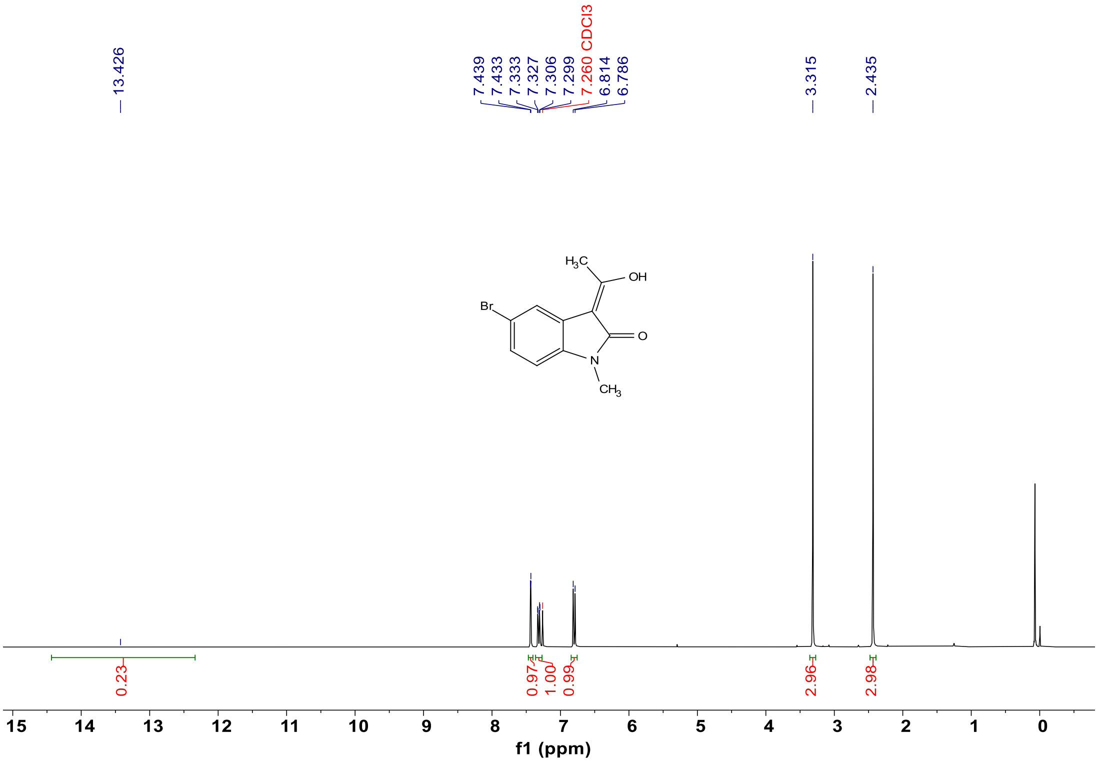

^{1}H NMR spectrum of compound **5n** (300 MHz, $CDCl_3$)

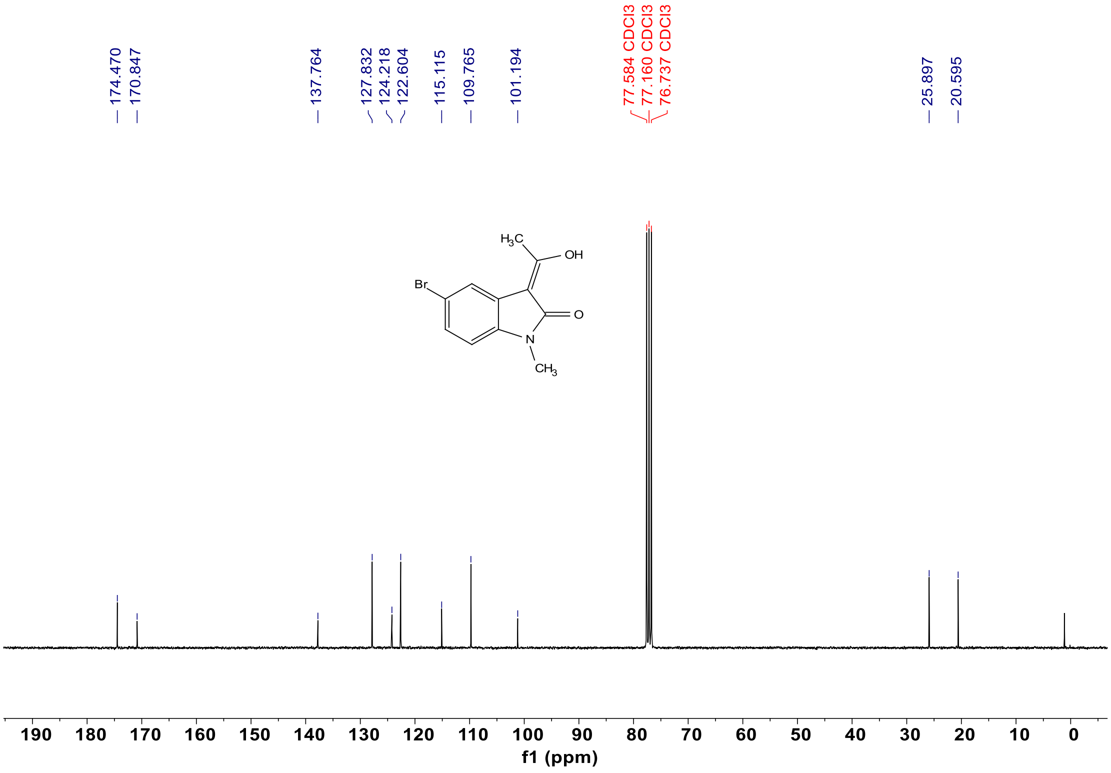

^{13}C NMR spectrum of compound **5n** (75 MHz, $CDCl_3$)

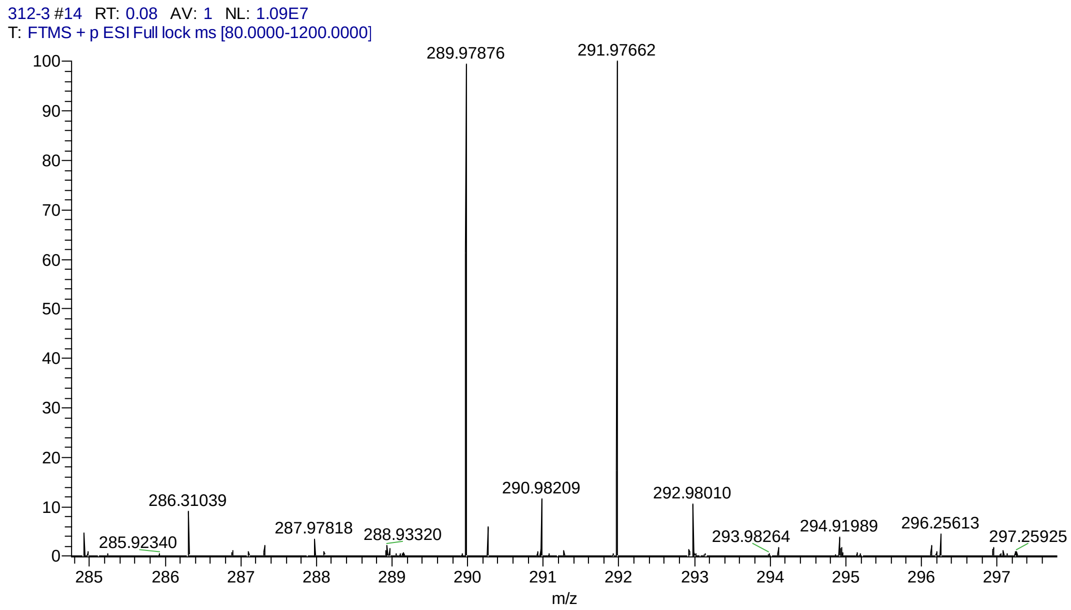

HRMS(ESI) of compound **5n**

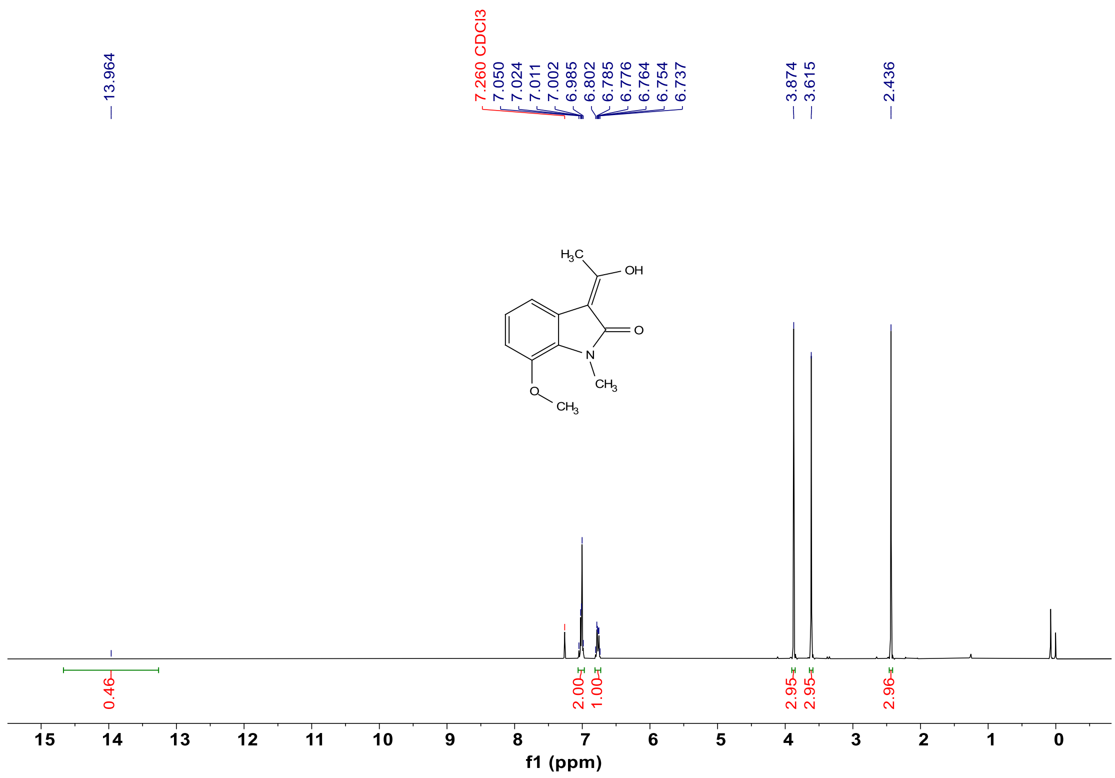

^{1}H NMR spectrum of compound **5o** (300 MHz, $CDCl_3$)

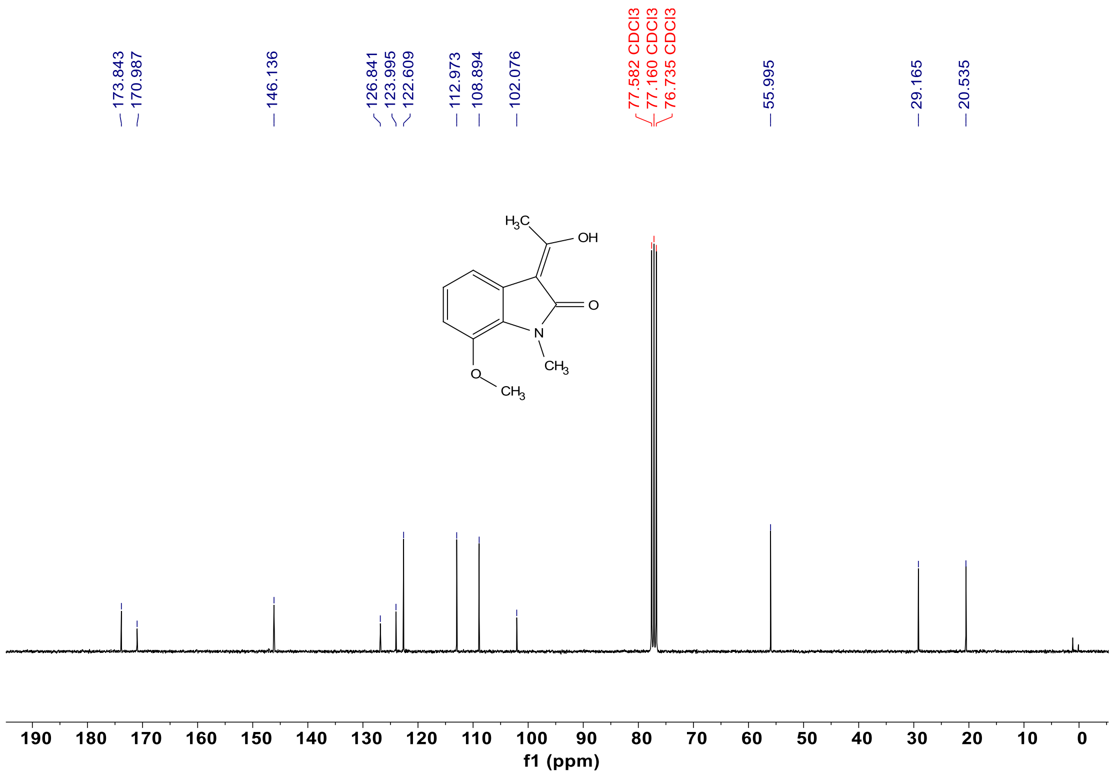

^{13}C NMR spectrum of compound **5o** (75 MHz, $CDCl_3$)

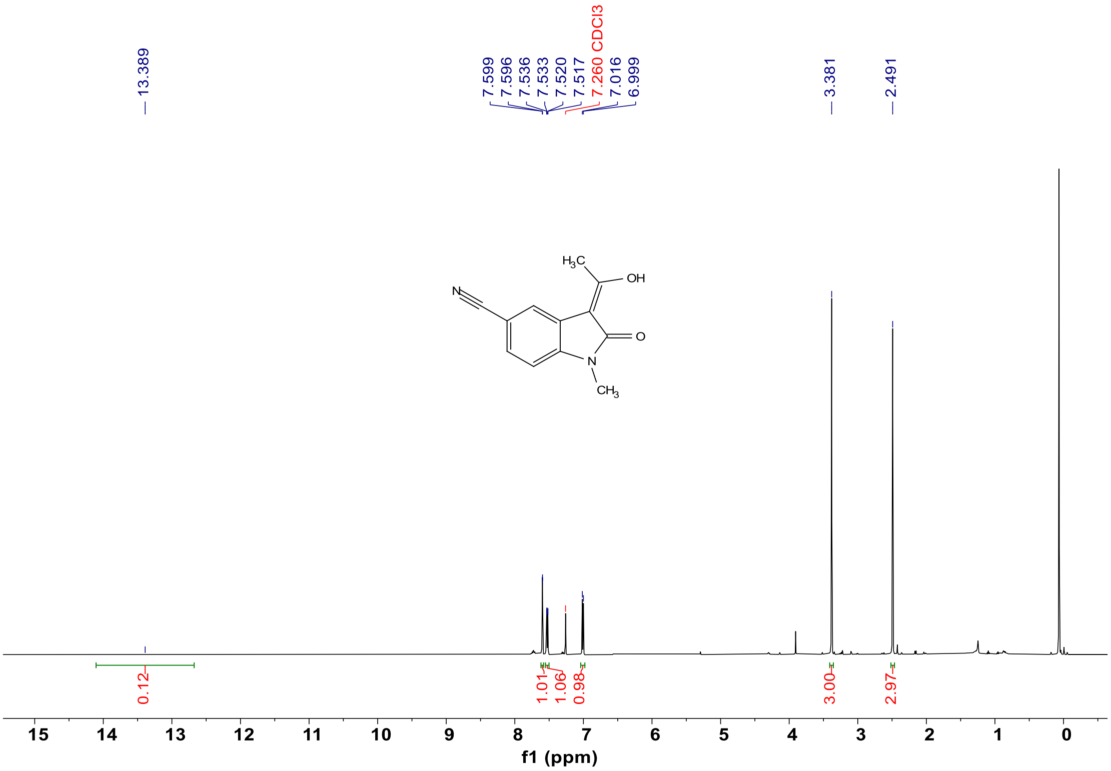

^{1}H NMR spectrum of compound **5p** (500 MHz, $CDCl_3$)

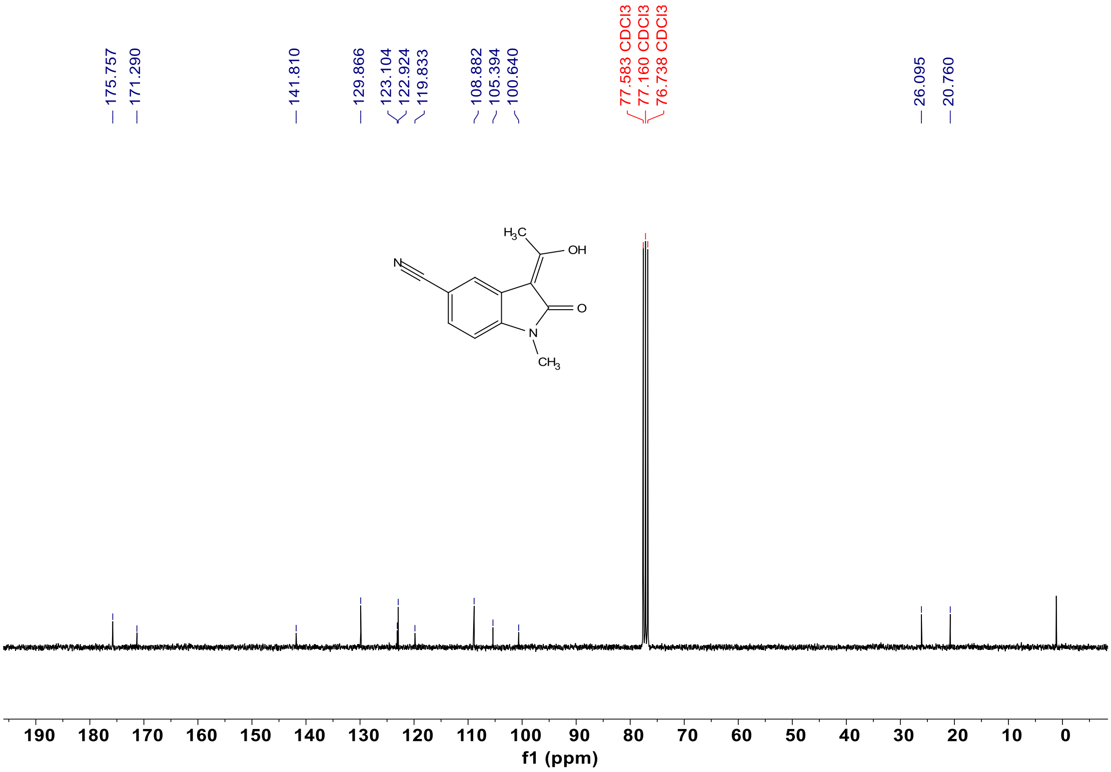

^{13}C NMR spectrum of compound **5p** (75 MHz, $CDCl_3$)

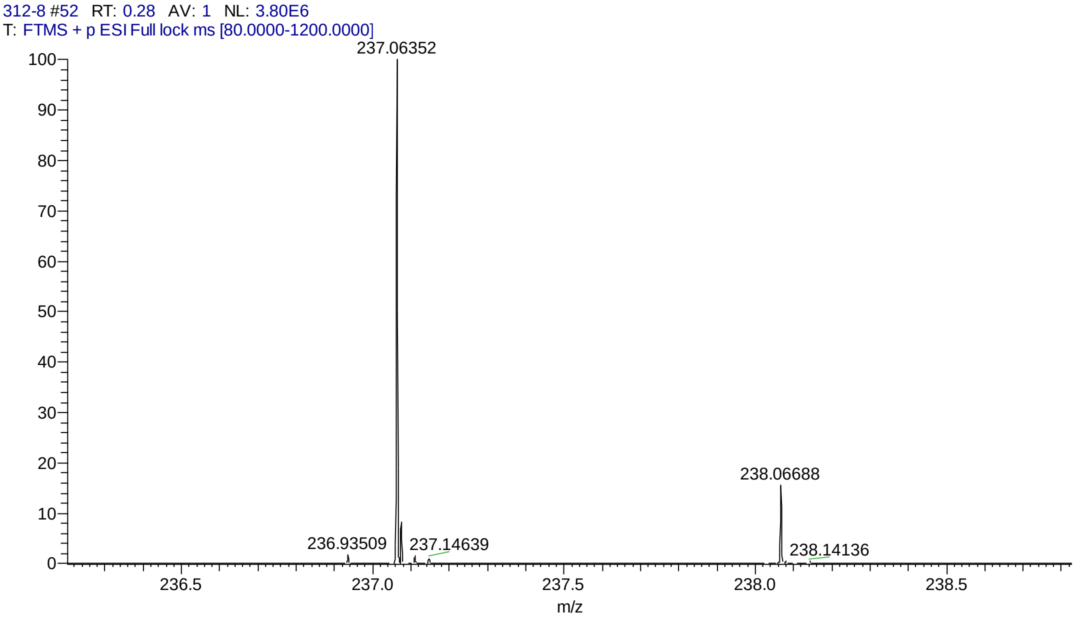

HRMS(ESI) of compound **5p**

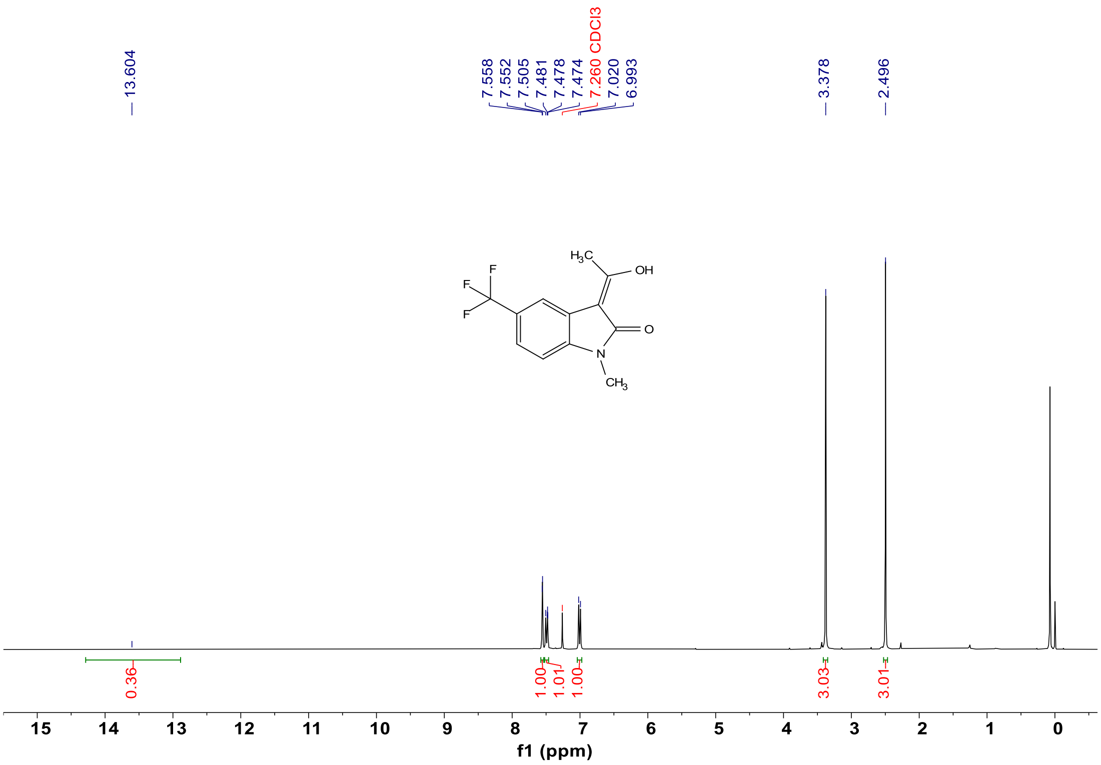

^{1}H NMR spectrum of compound **5q** (300 MHz, $CDCl_3$)

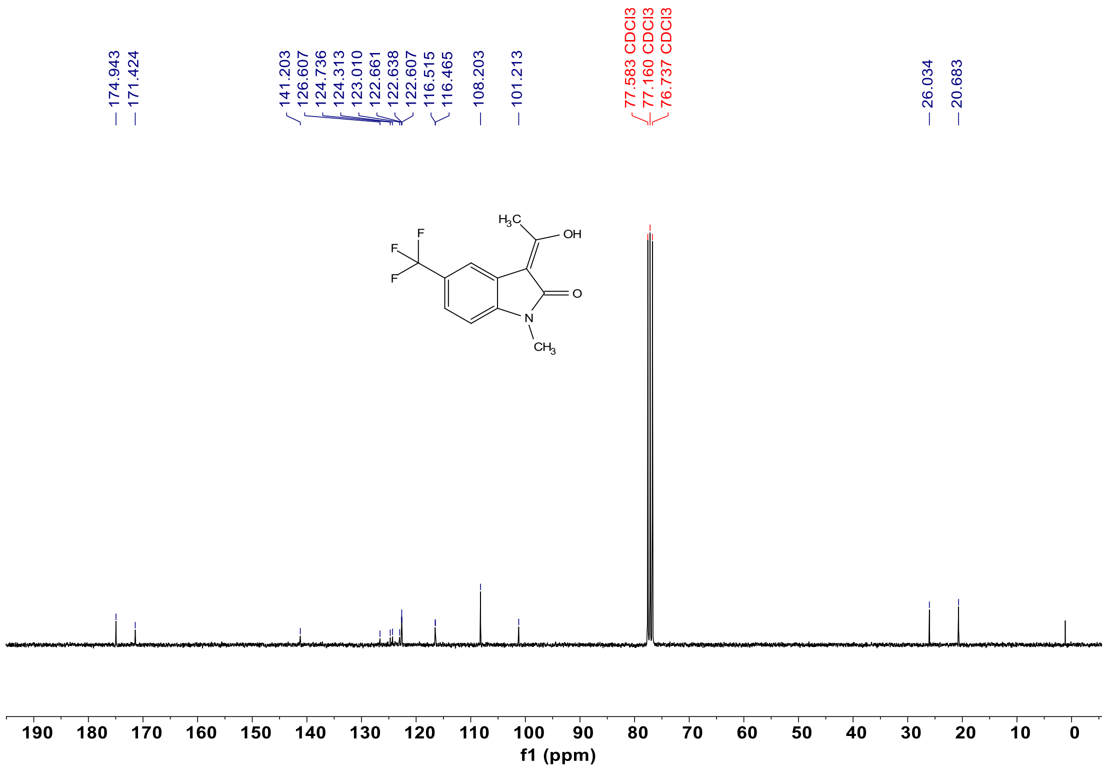

^{13}C NMR spectrum of compound **5q** (75 MHz, $CDCl_3$)

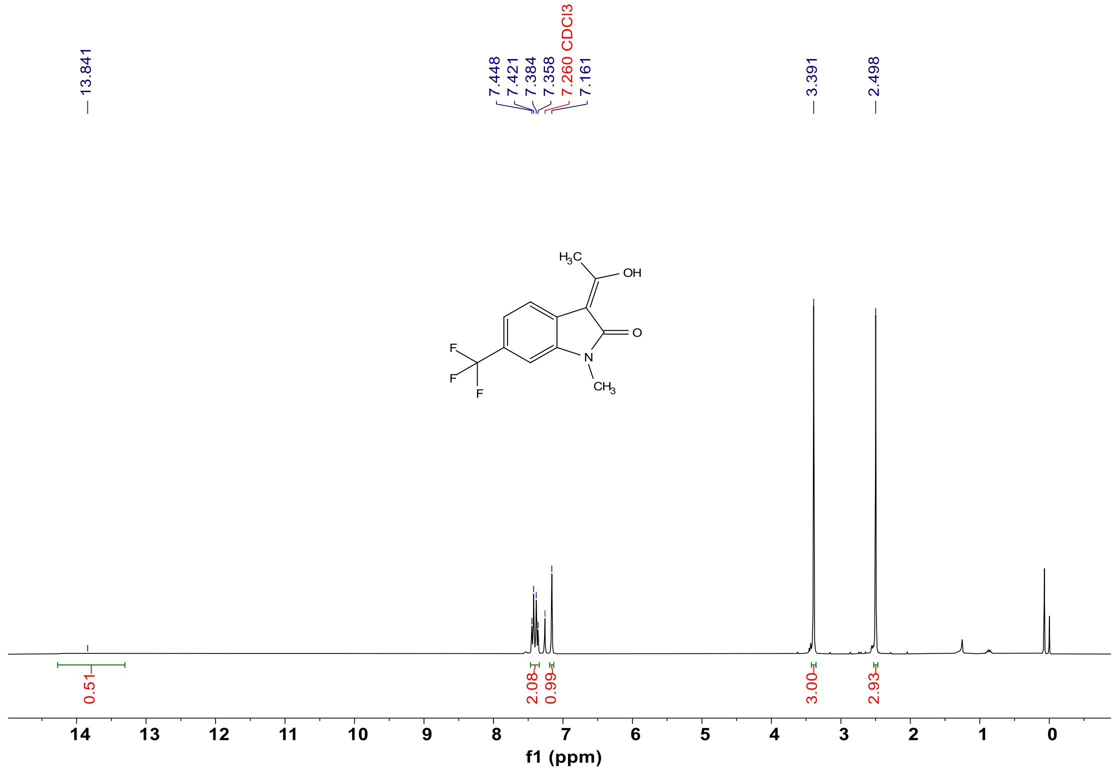

^{1}H NMR spectrum of compound **5r** (300 MHz, $CDCl_3$)

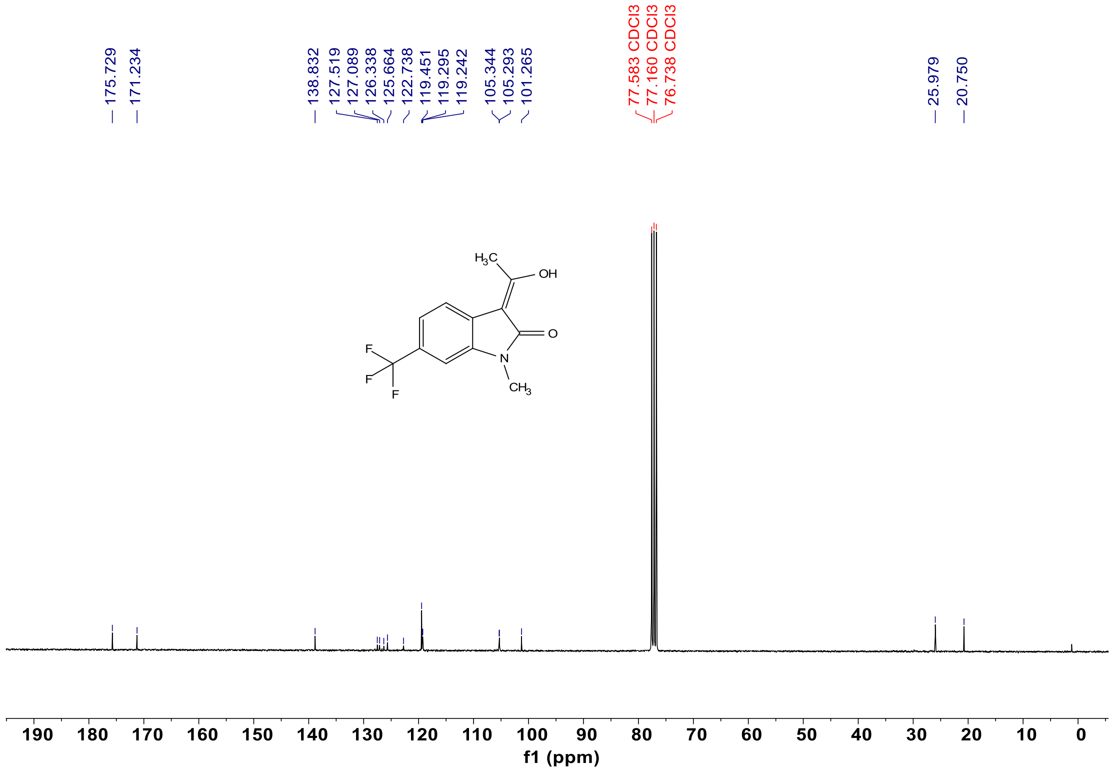

^{13}C NMR spectrum of compound **5r** (75 MHz, $CDCl_3$)

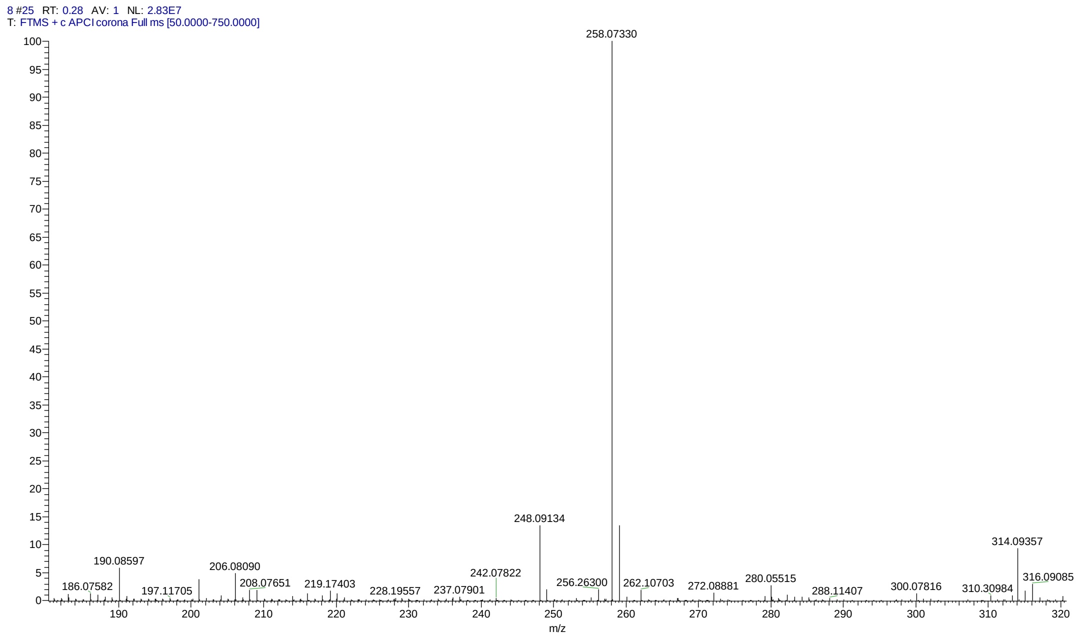

HRMS(APCI) of compound **5r**

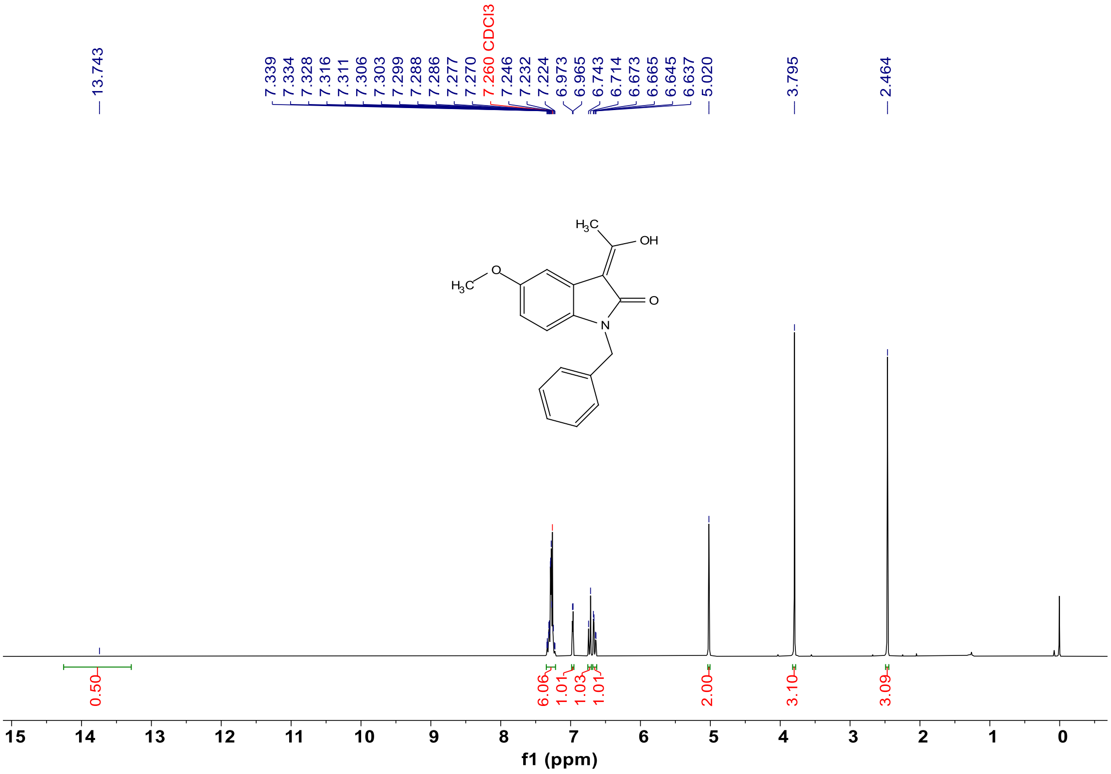

^{1}H NMR spectrum of compound **5s** (300 MHz, $CDCl_3$)

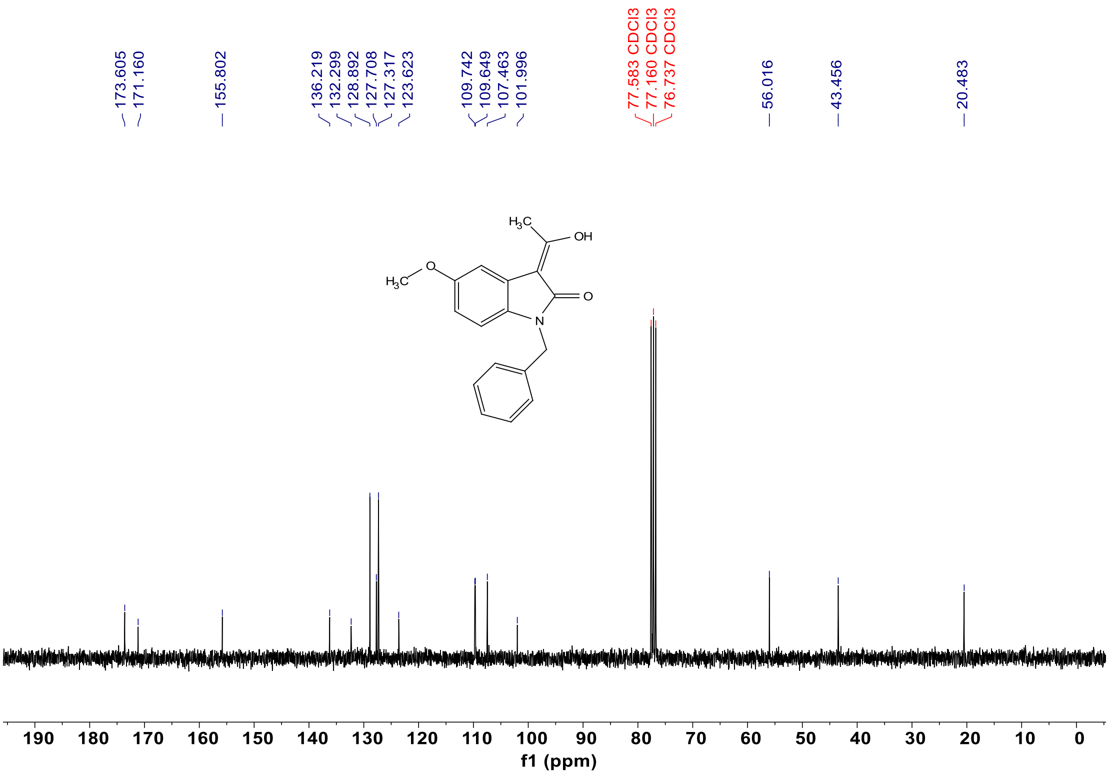

^{13}C NMR spectrum of compound **5s** (75 MHz, $CDCl_3$)

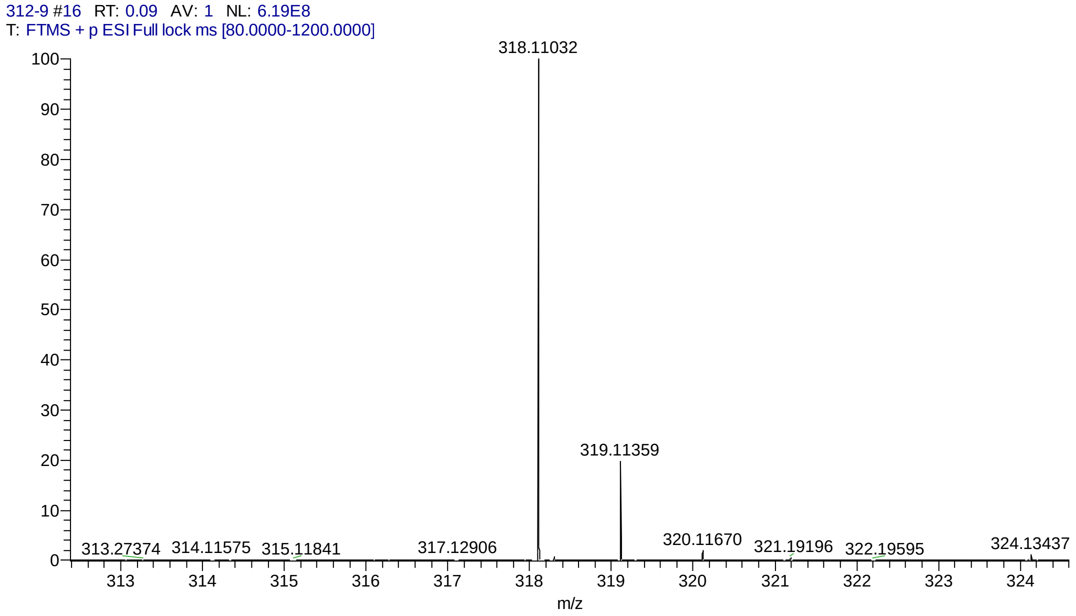

HRMS(ESI) of compound **5s**

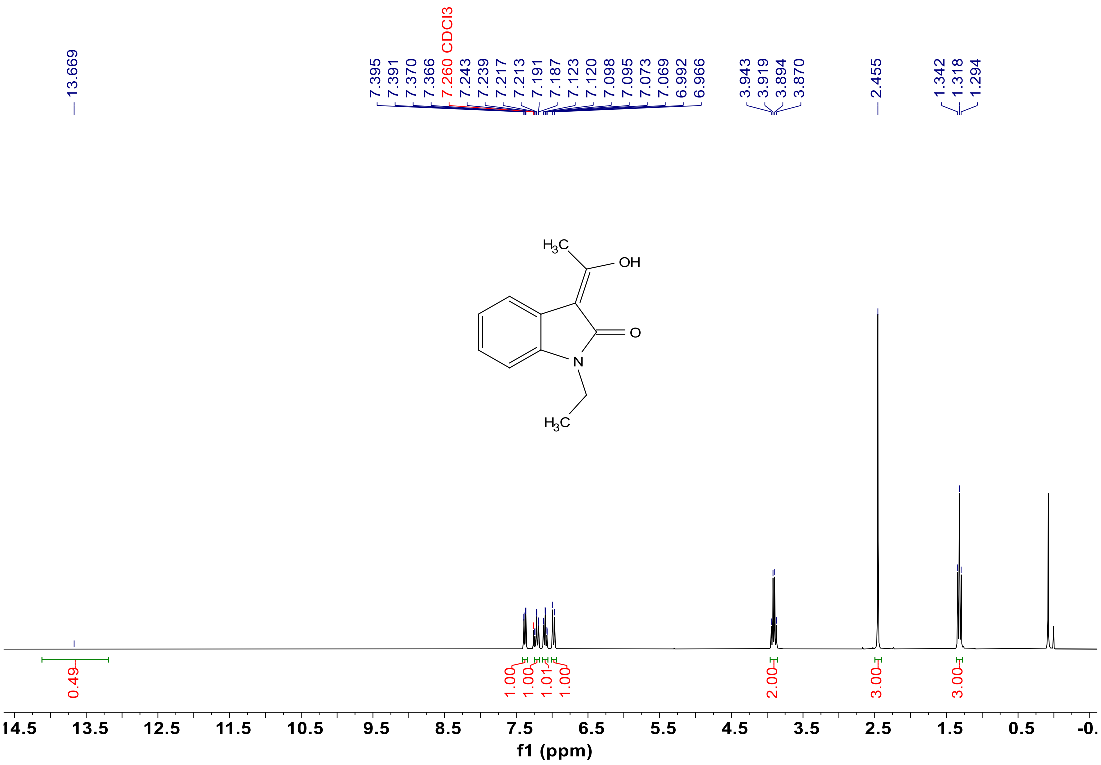

^{1}H NMR spectrum of compound **5t** (300 MHz, $CDCl_3$)

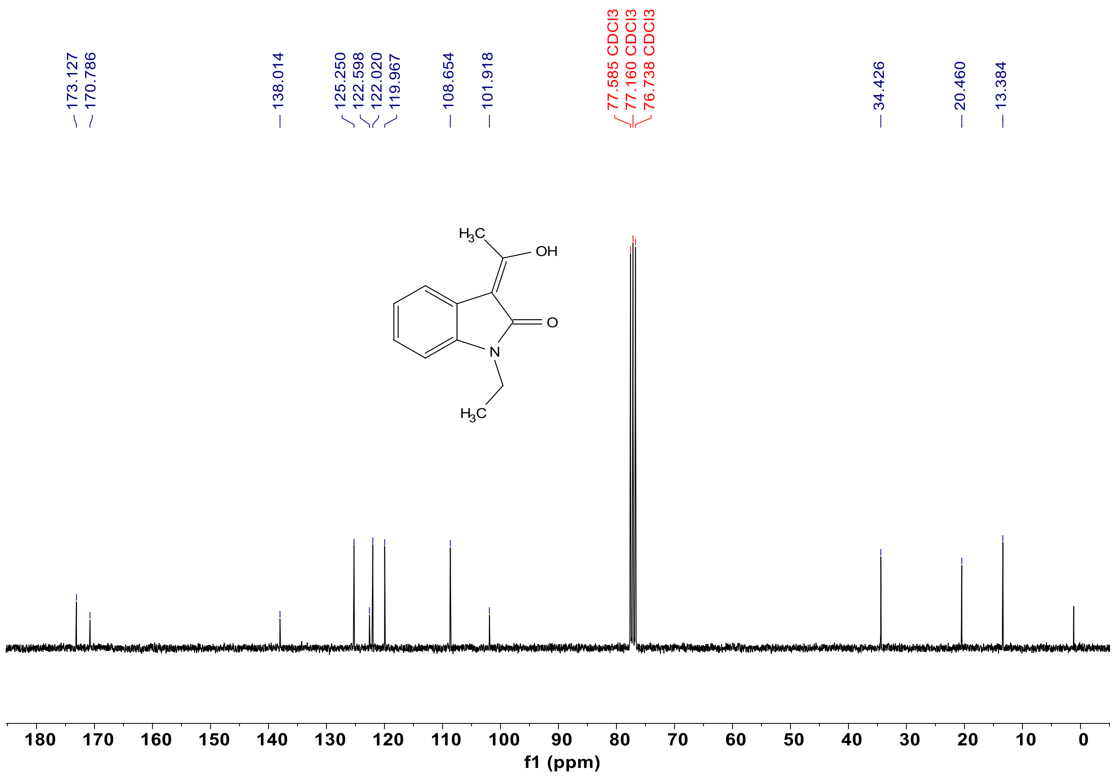

^{13}C NMR spectrum of compound **5t** (75 MHz, $CDCl_3$)

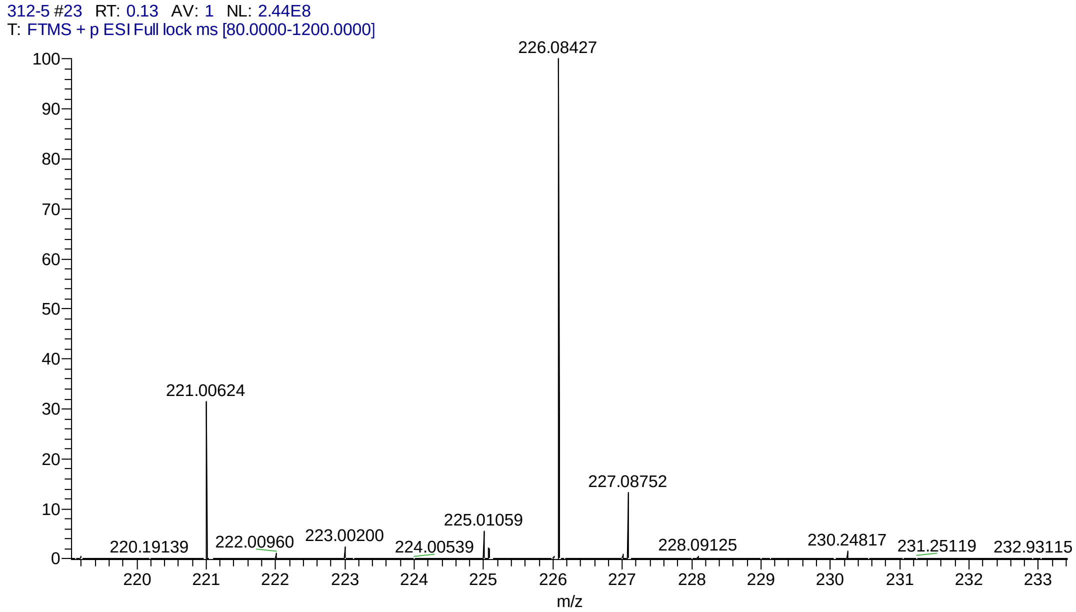

HRMS(ESI) of compound **5t**

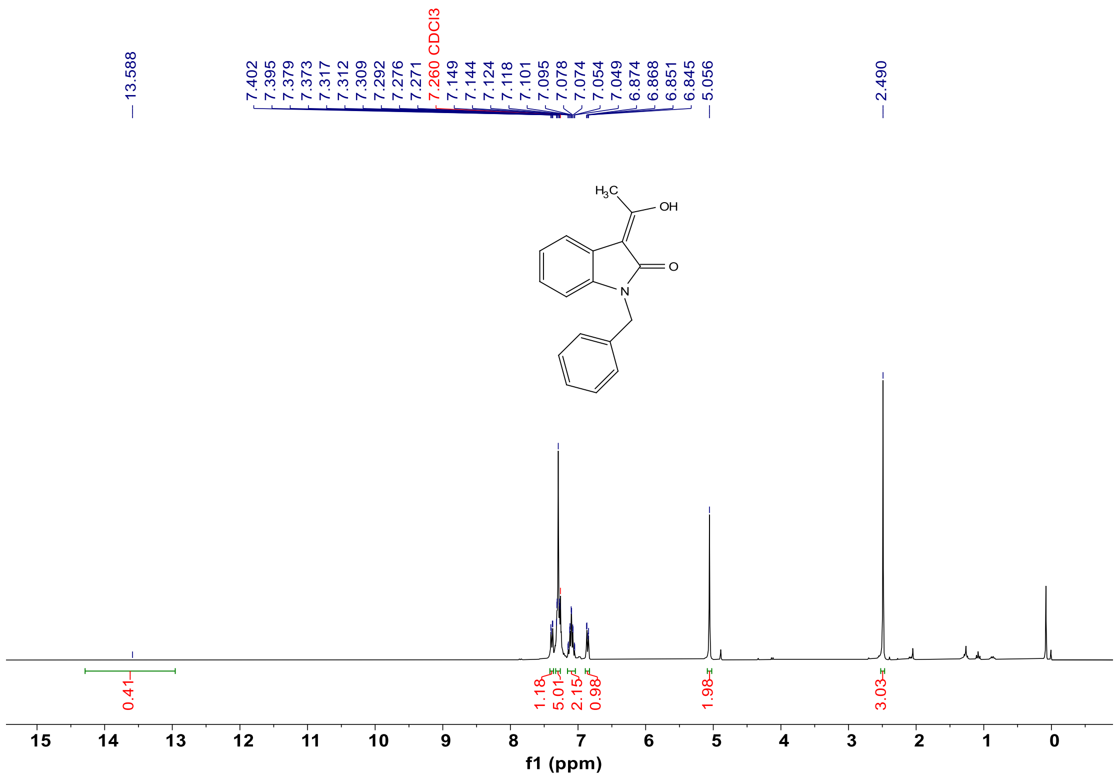

^{1}H NMR spectrum of compound **5u** (300 MHz, $CDCl_3$)

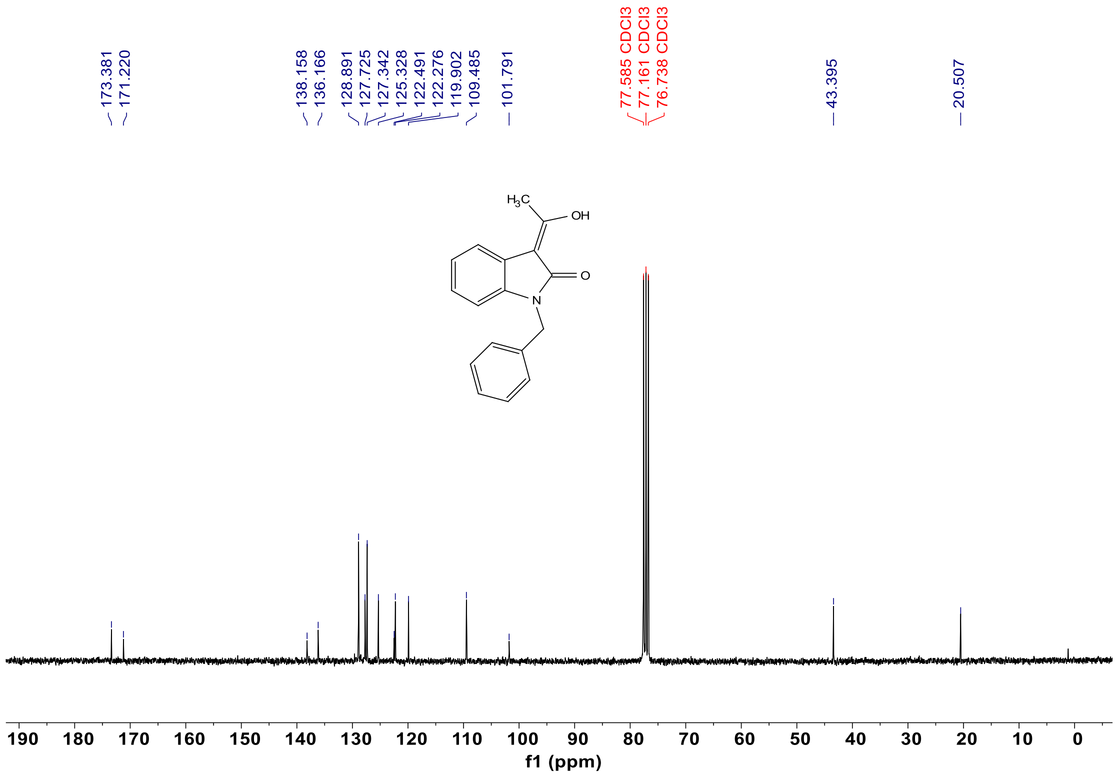

^{13}C NMR spectrum of compound **5u** (75 MHz, $CDCl_3$)

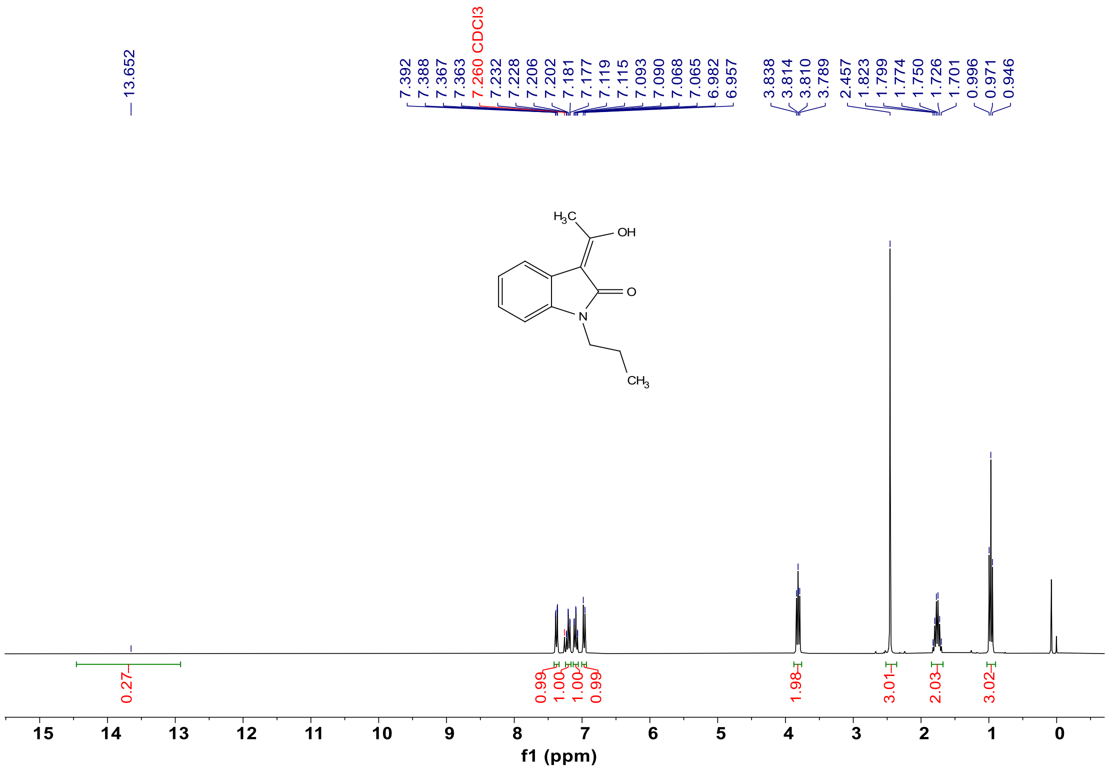

^{1}H NMR spectrum of compound **5v** (300 MHz, $CDCl_3$)

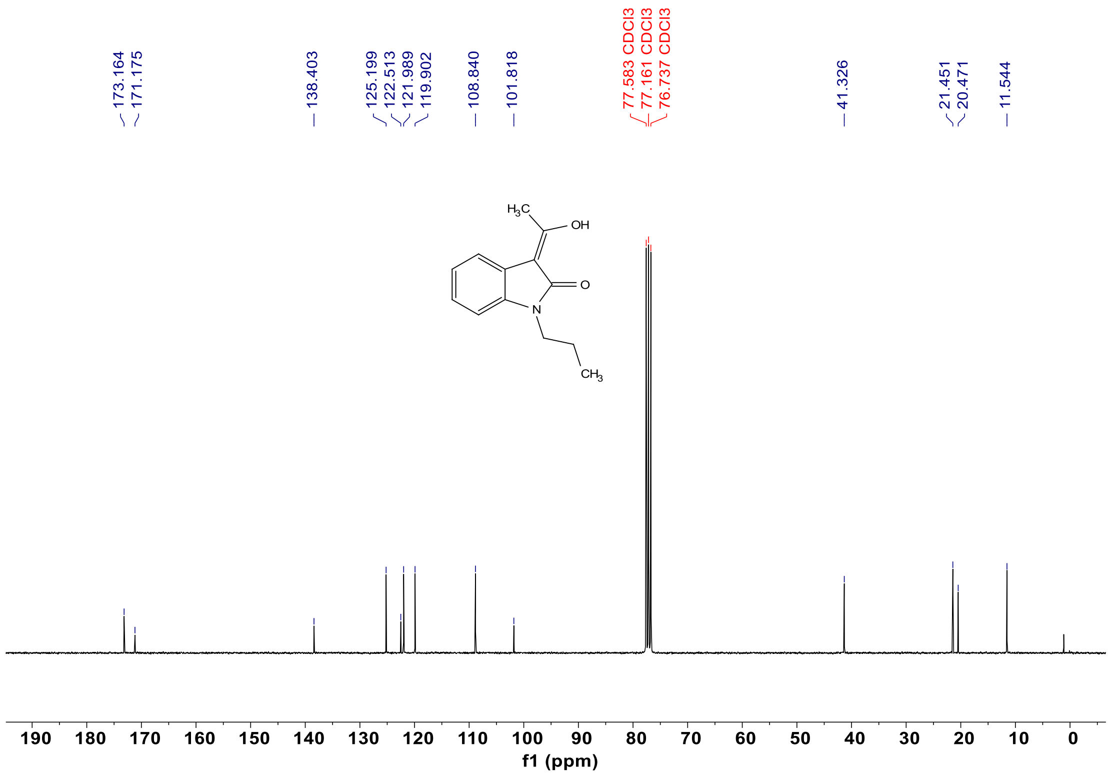

^{13}C NMR spectrum of compound **5v** (75 MHz, $CDCl_3$)

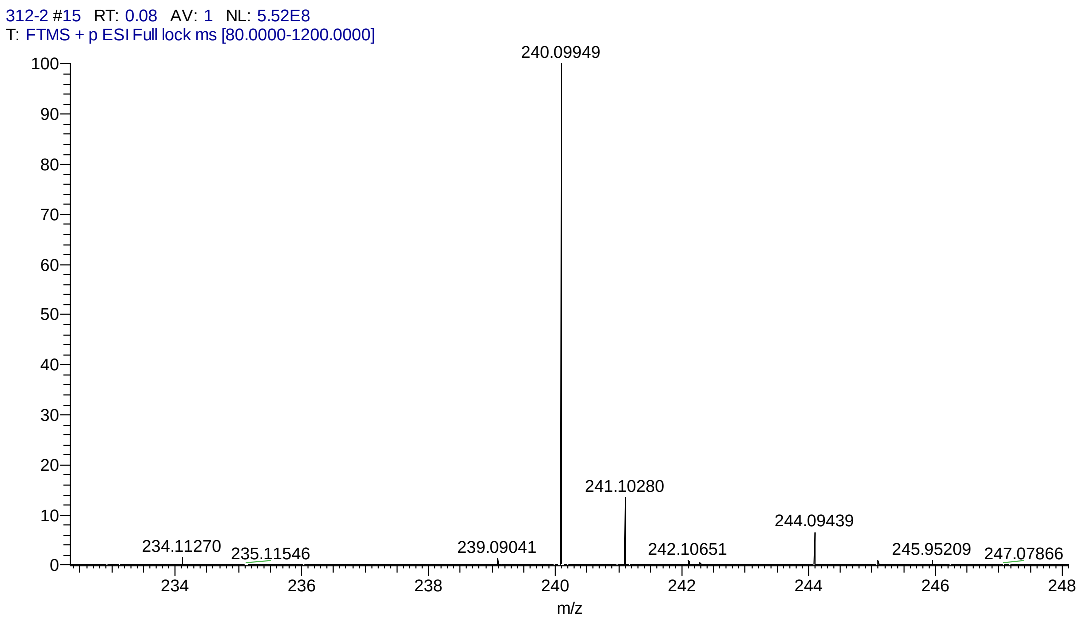

HRMS(ESI) of compound **5v**

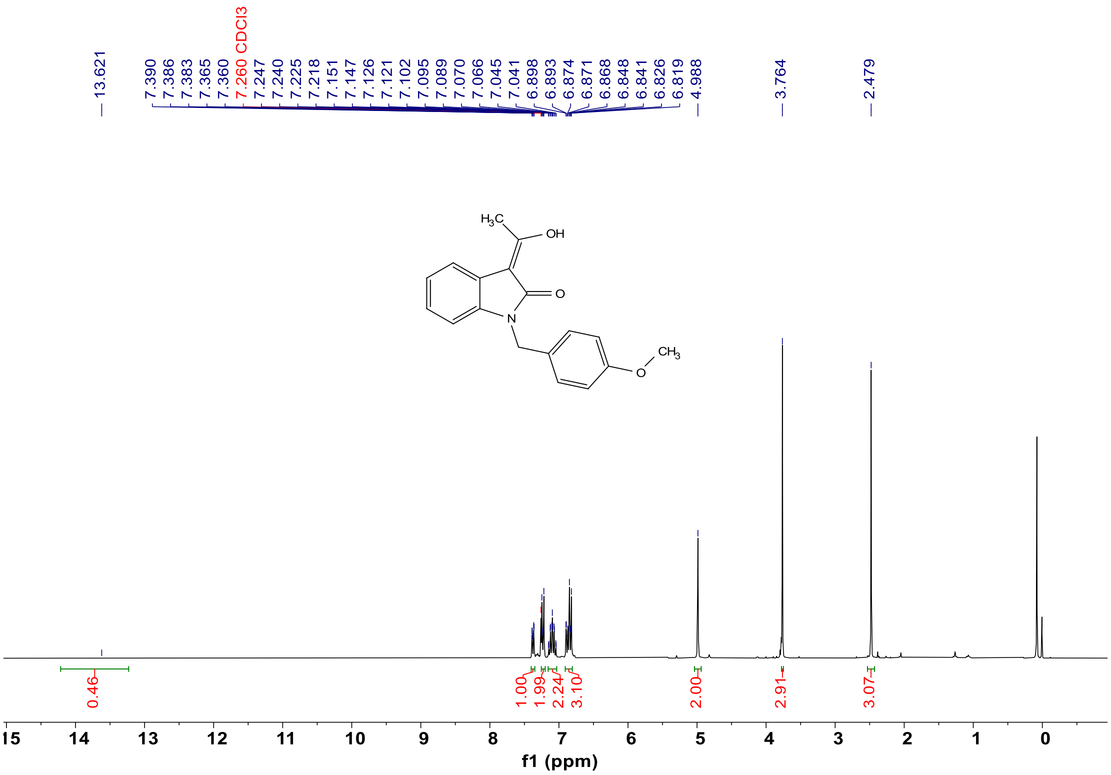

^{1}H NMR spectrum of compound **5w** (300 MHz, $CDCl_3$)

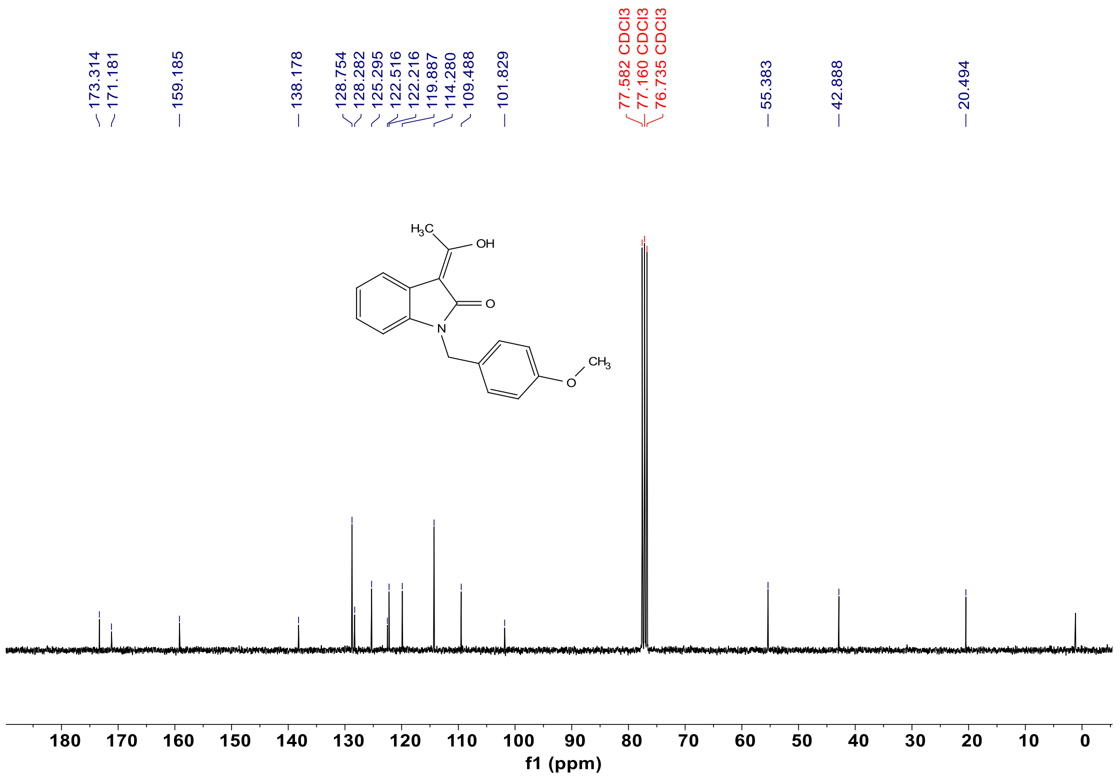

^{13}C NMR spectrum of compound **5w** (75 MHz, $CDCl_3$)

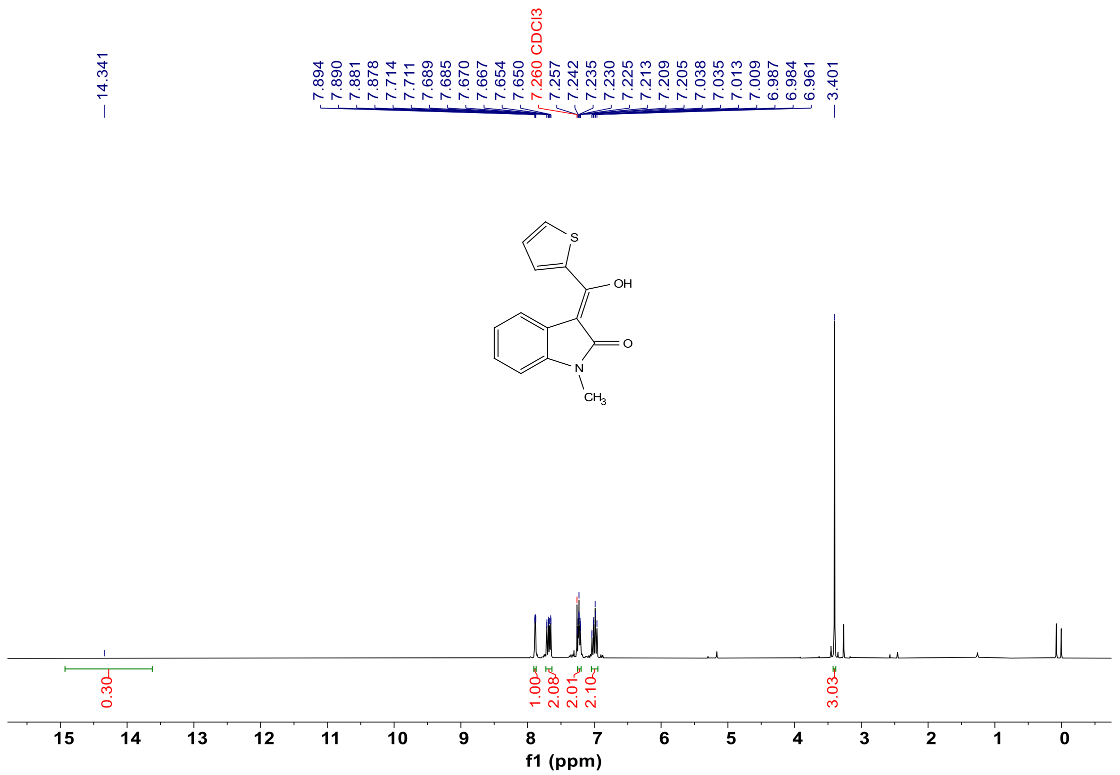

^{1}H NMR spectrum of compound **10a** (300 MHz, $CDCl_3$)

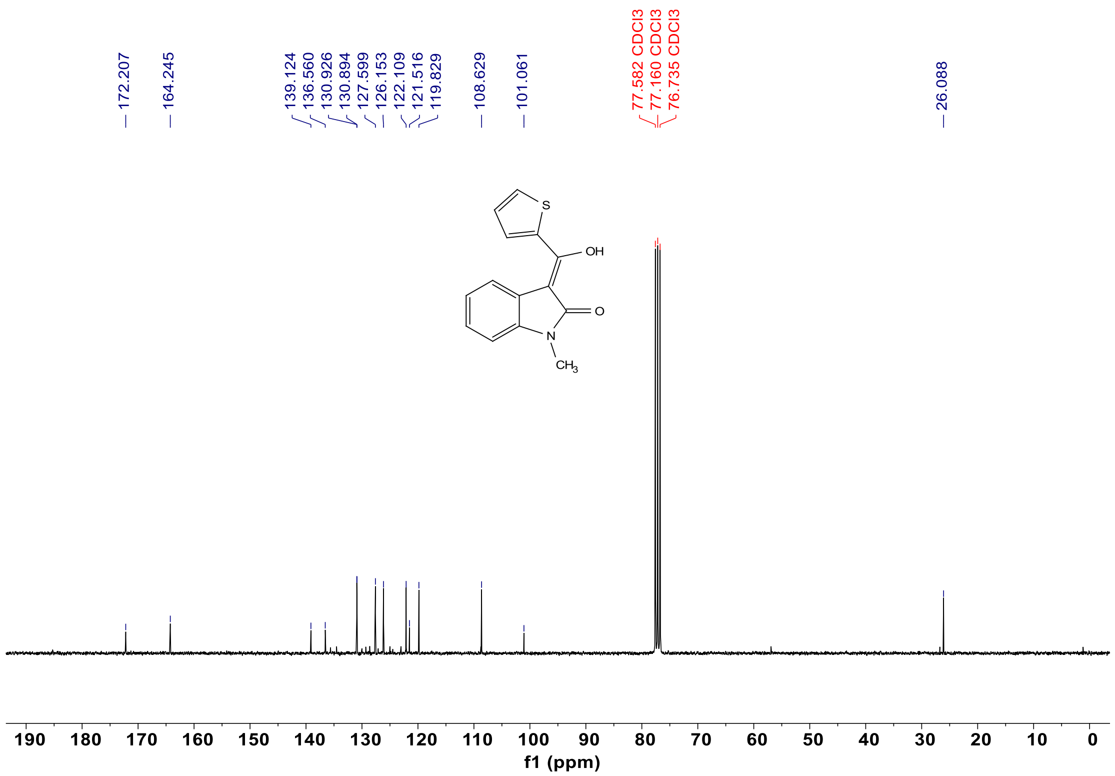

^{13}C NMR spectrum of compound **10a** (75 MHz, $CDCl_3$)

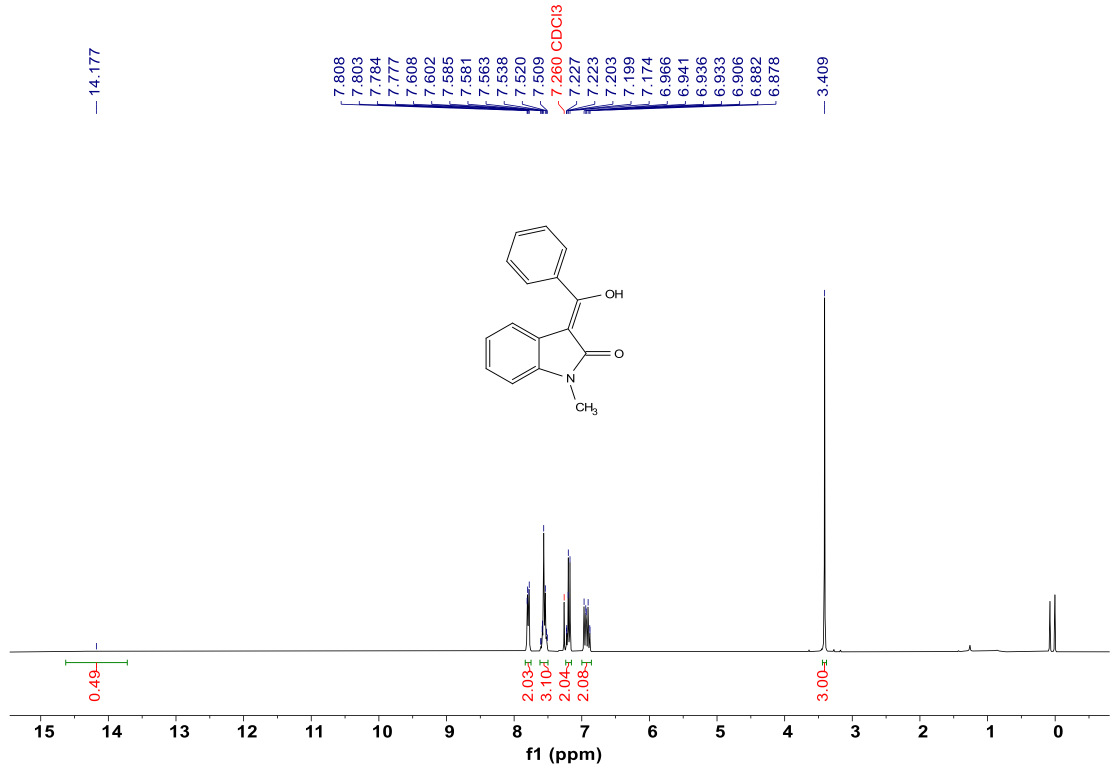

^{1}H NMR spectrum of compound **10b** (300 MHz, $CDCl_3$)

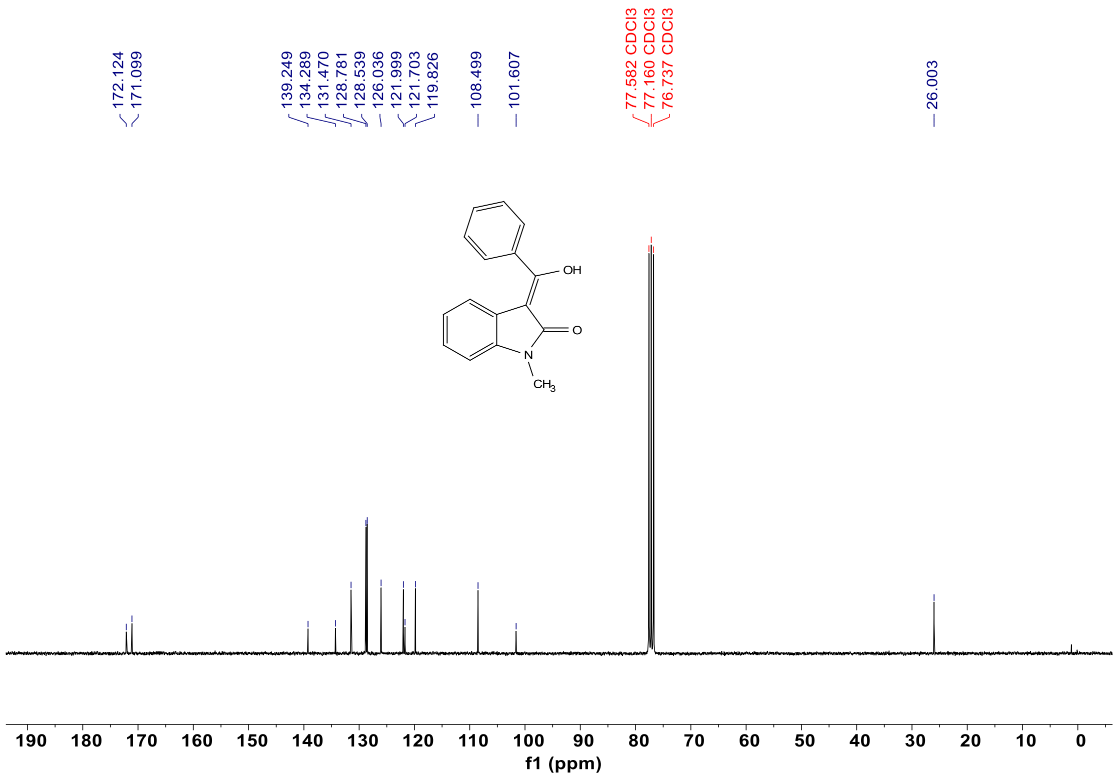

^{13}C NMR spectrum of compound **10b** (75 MHz, $CDCl_3$)

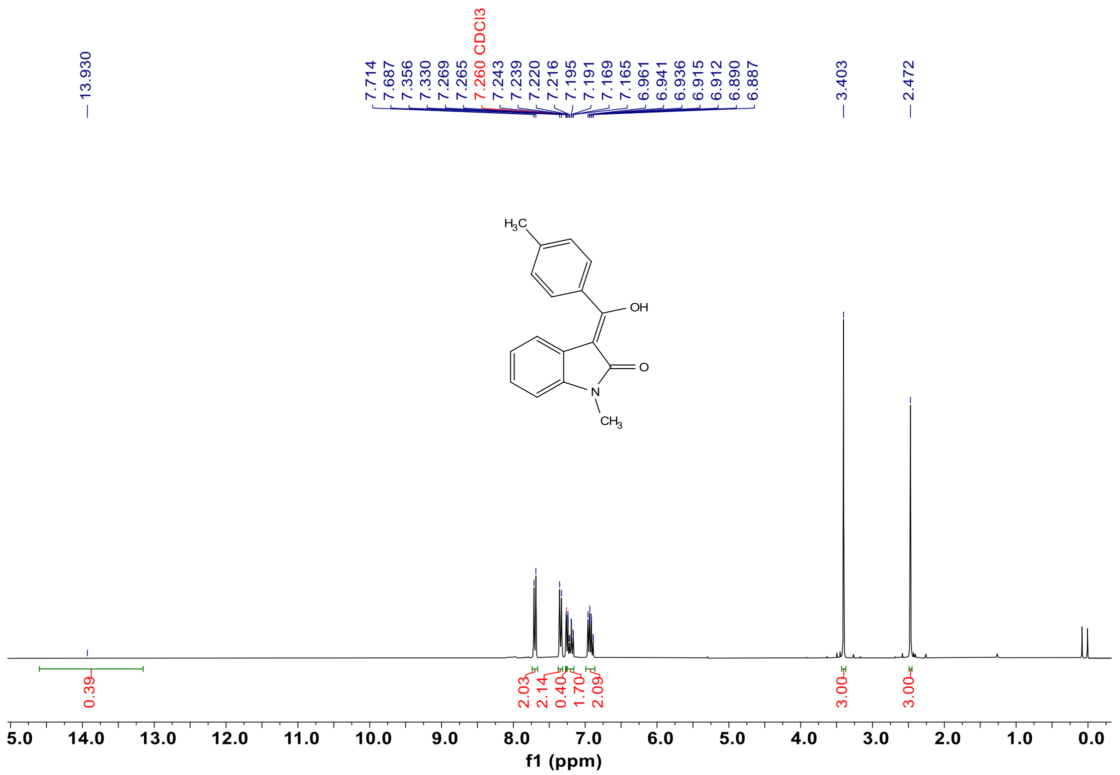

^{1}H NMR spectrum of compound **10c** (300 MHz, $CDCl_3$)

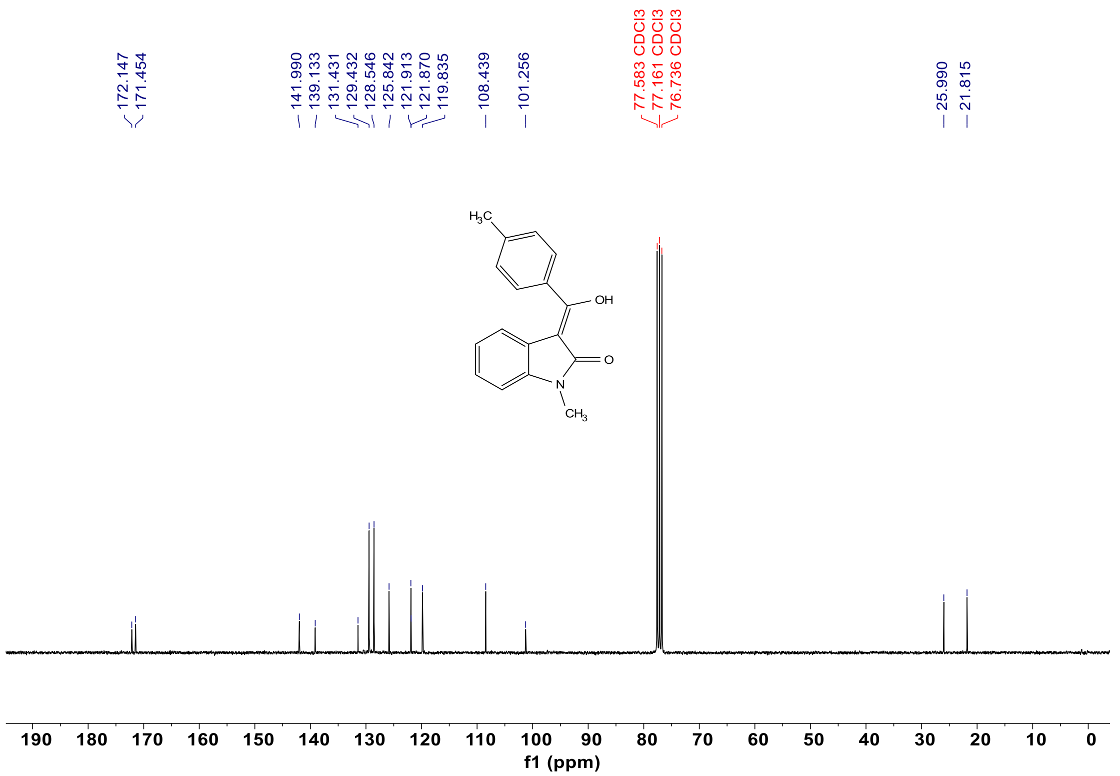

^{13}C NMR spectrum of compound **10c** (75 MHz, $CDCl_3$)

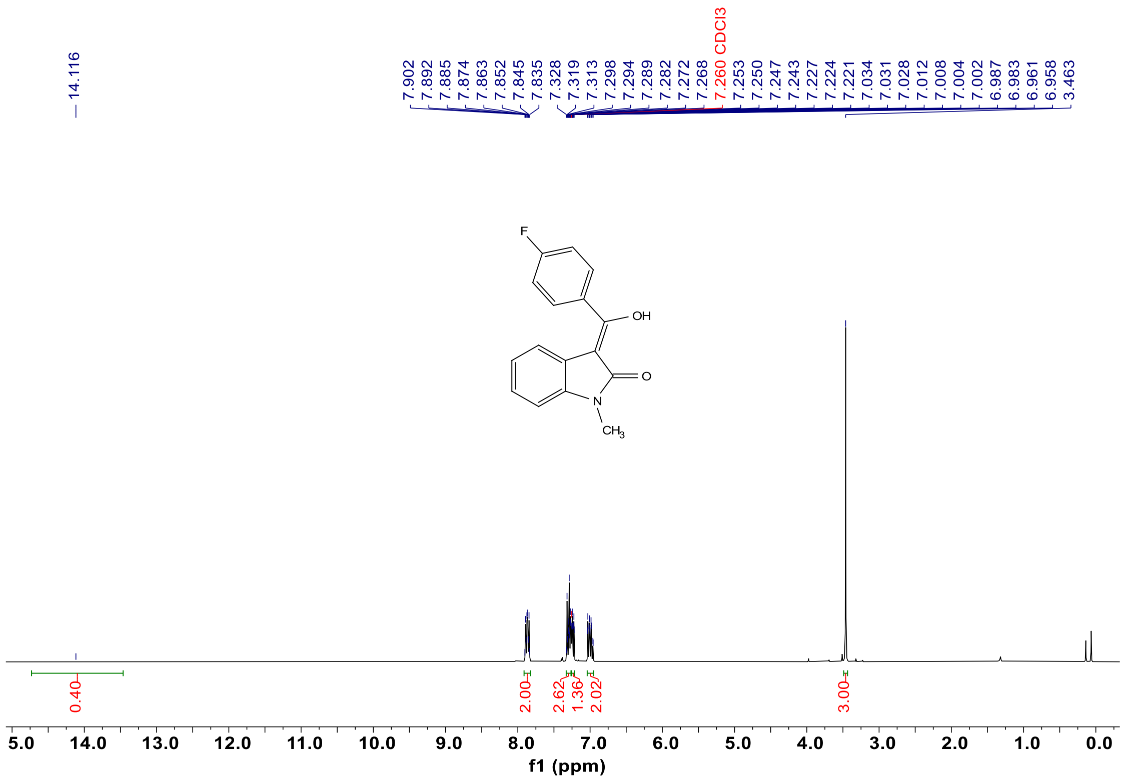

^{1}H NMR spectrum of compound **10d** (300 MHz, $CDCl_3$)

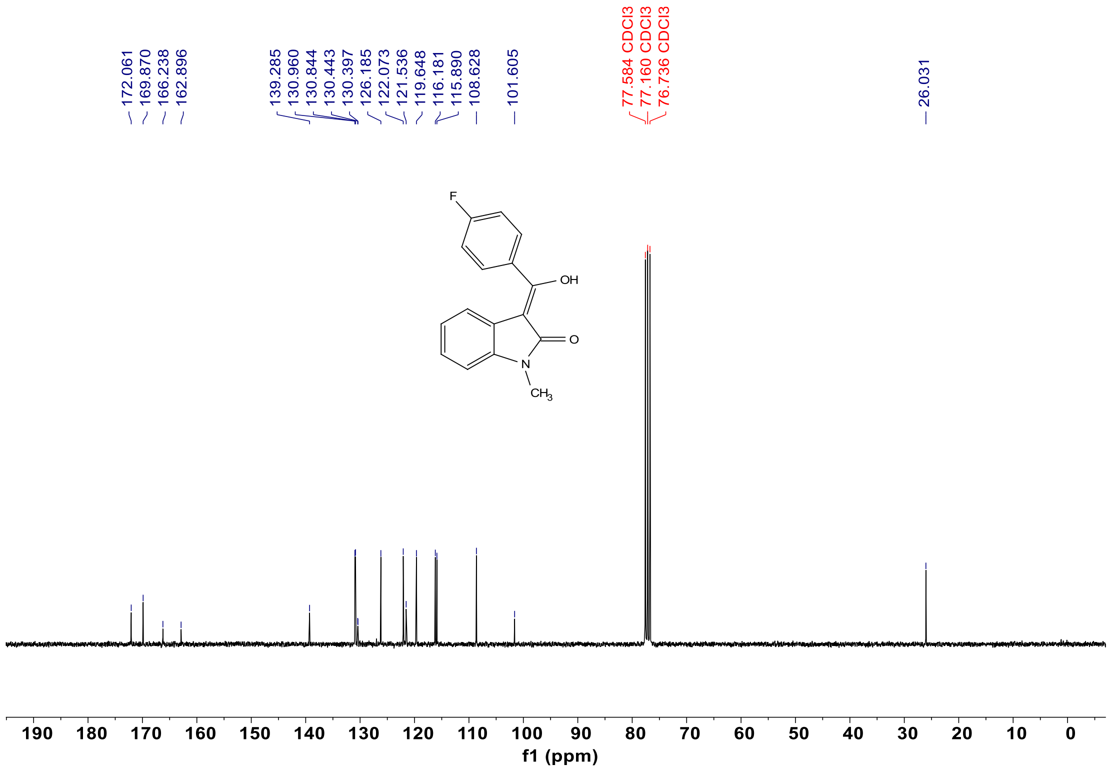

^{13}C NMR spectrum of compound **10d** (75 MHz, $CDCl_3$)

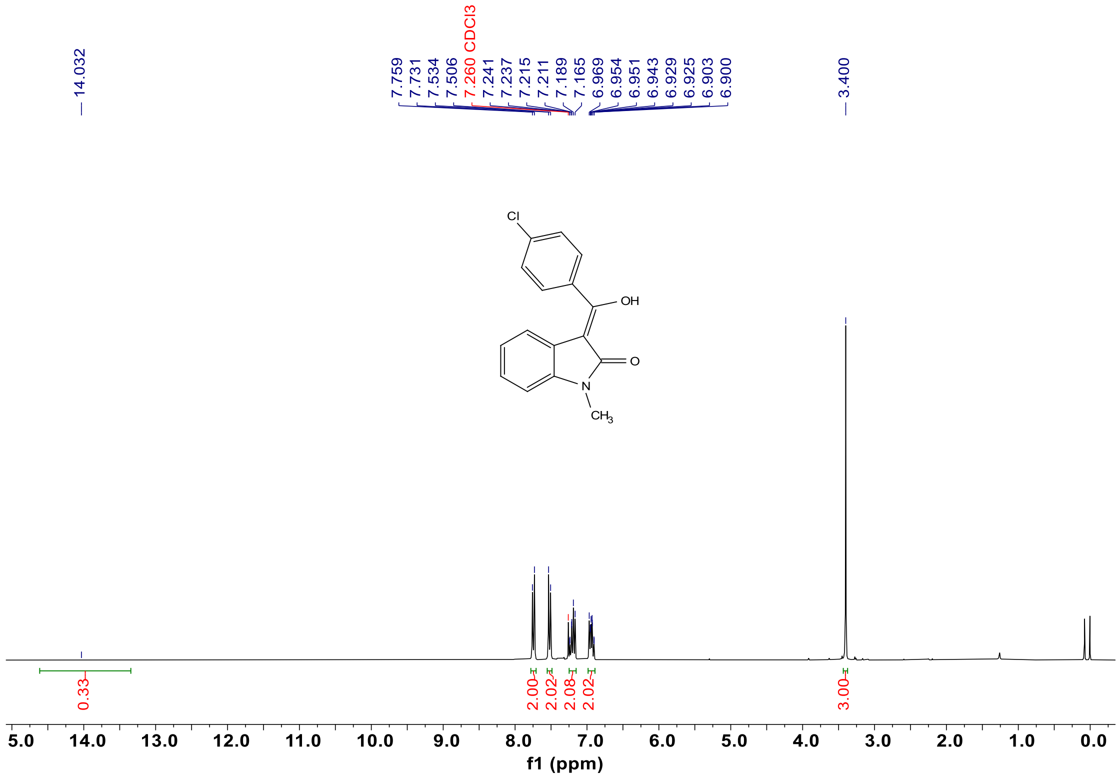

^{1}H NMR spectrum of compound **10e** (75 MHz, $CDCl_3$)

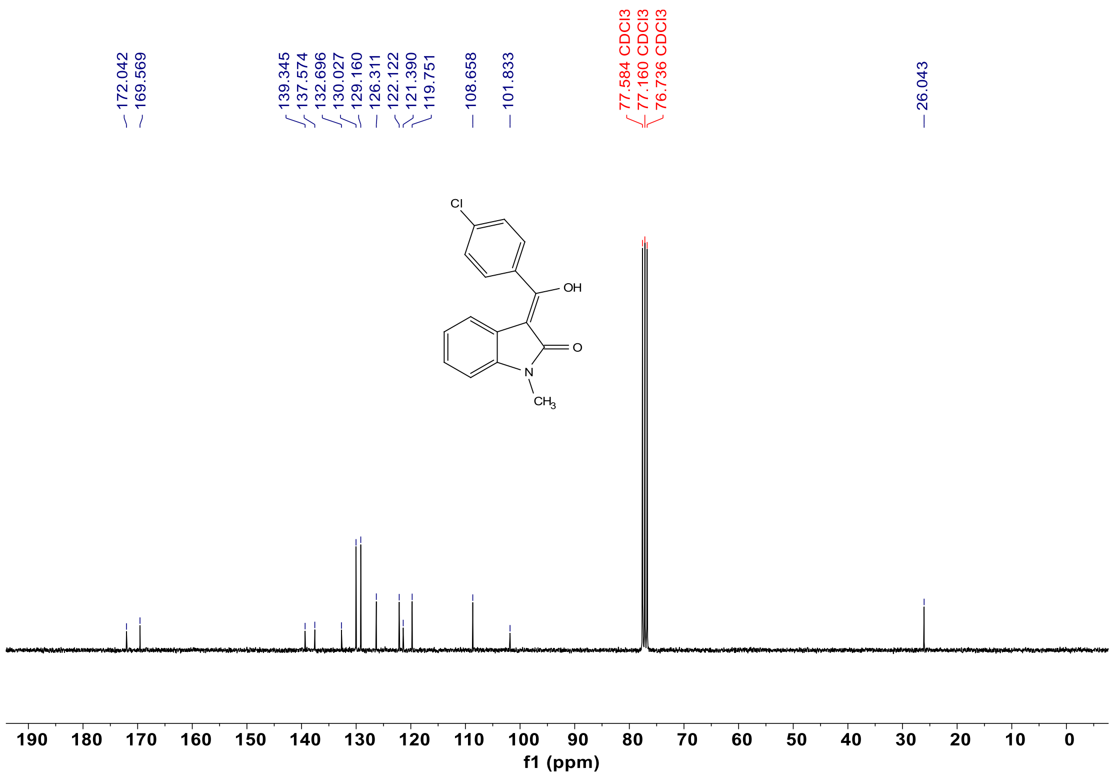

^{13}C NMR spectrum of compound **10e** (75 MHz, $CDCl_3$)

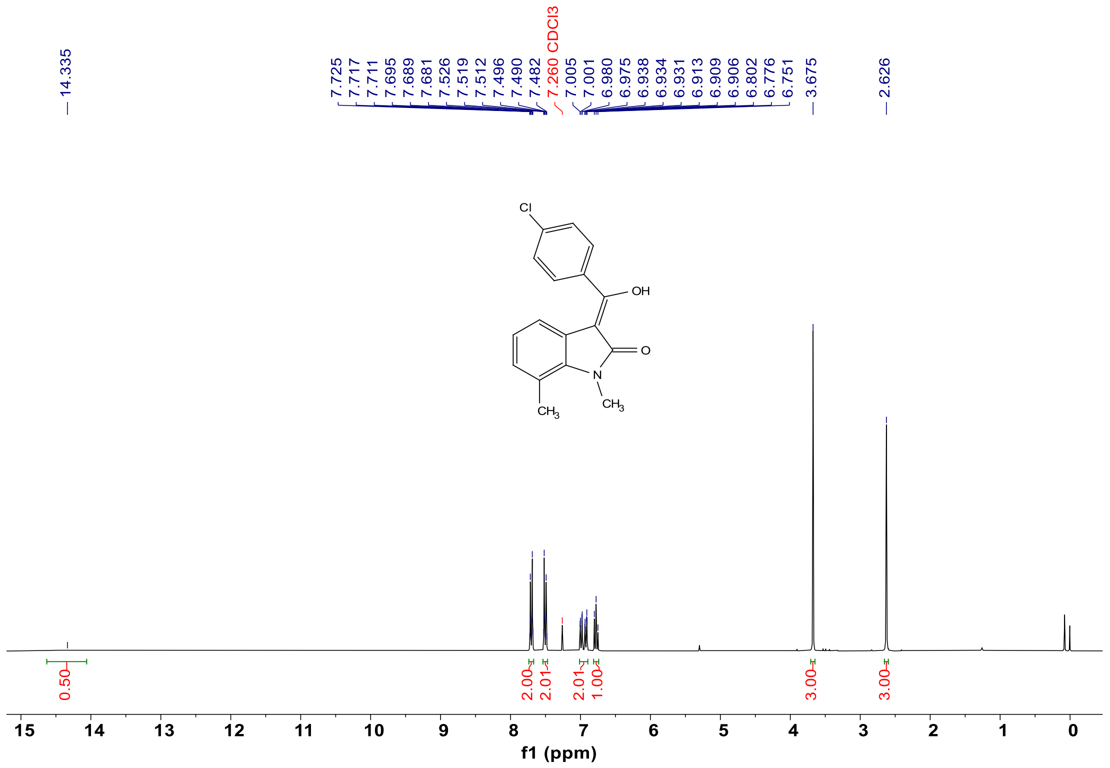

^{1}H NMR spectrum of compound **10f** (300 MHz, $CDCl_3$)

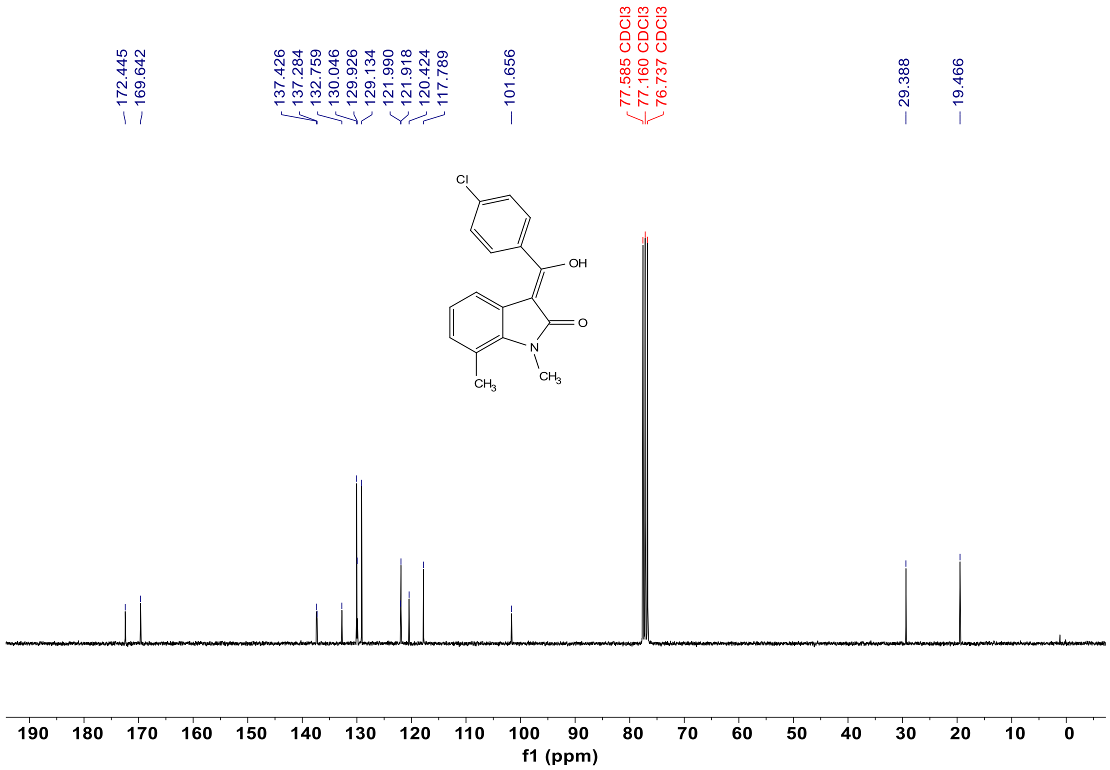

^{13}C NMR spectrum of compound **10f** (75 MHz, $CDCl_3$)

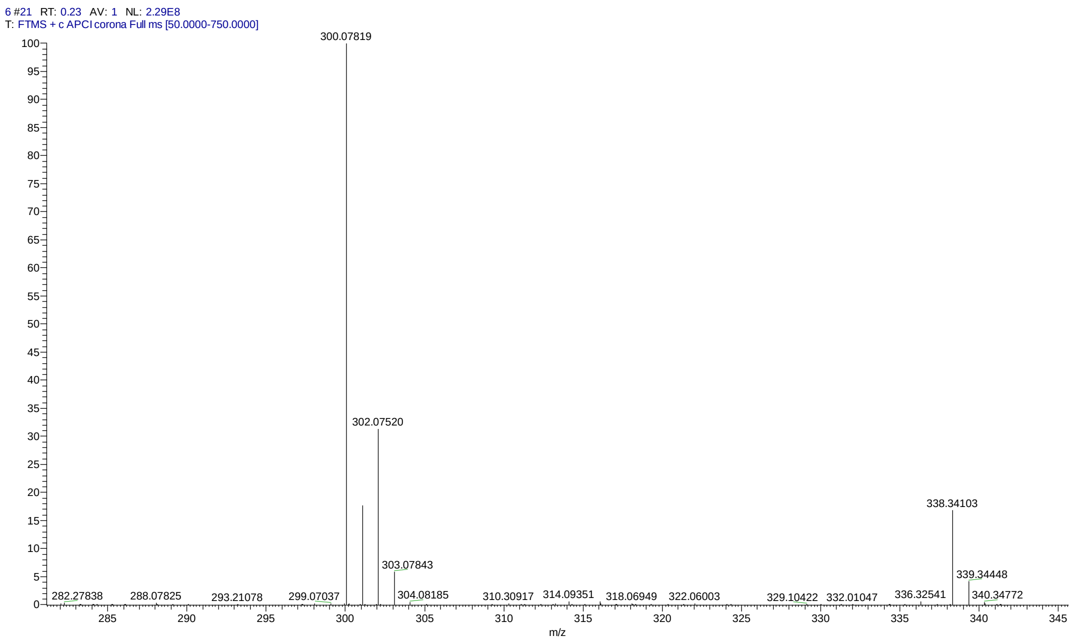

HRMS(APCI) of compound **10f**

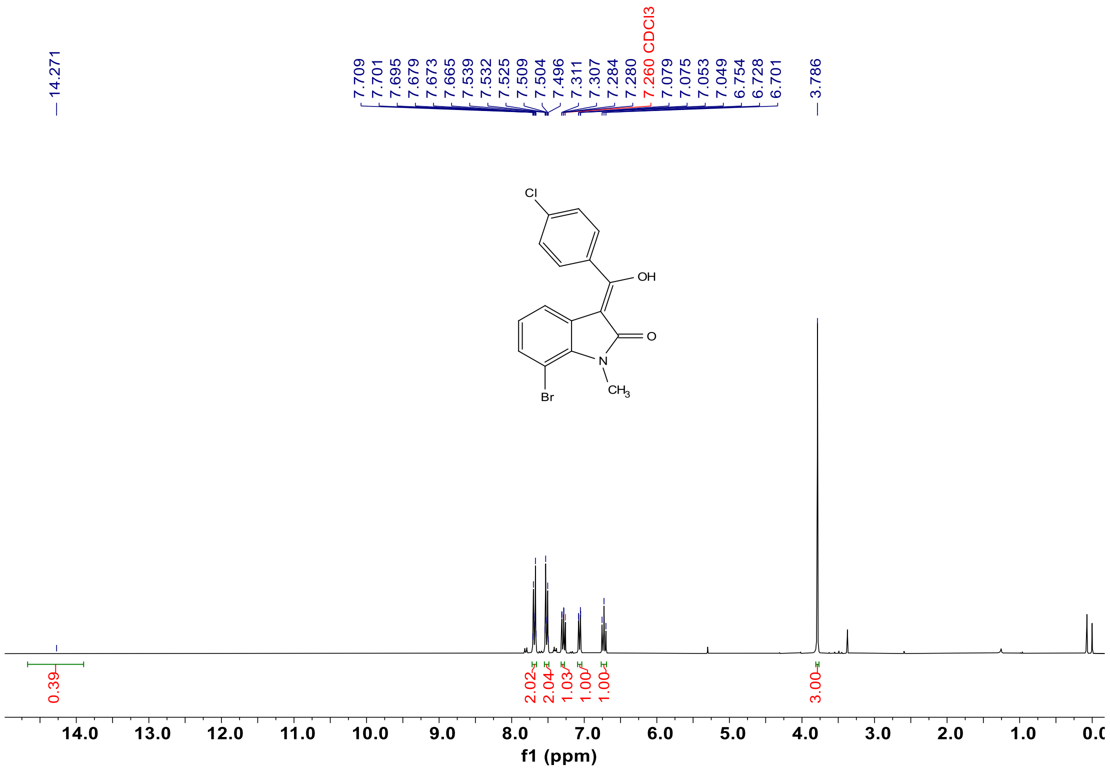

^{1}H NMR spectrum of compound **10g** (300 MHz, $CDCl_3$)

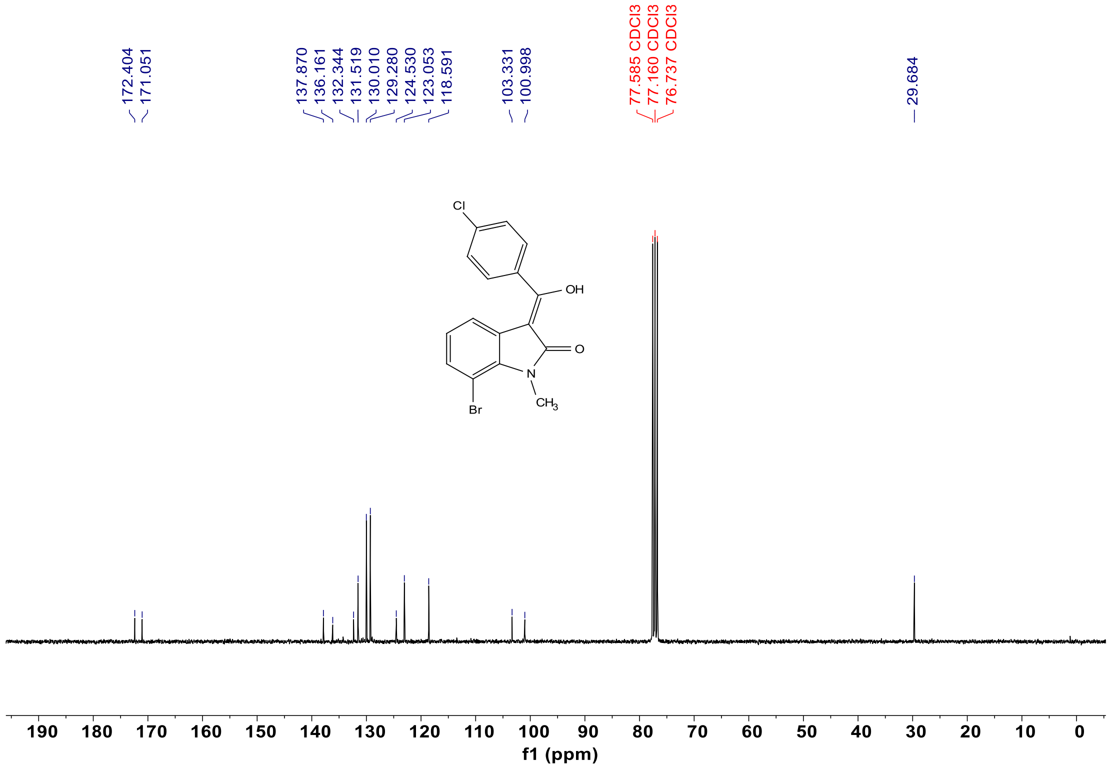

^{13}C NMR spectrum of compound **10g** (75 MHz, $CDCl_3$)

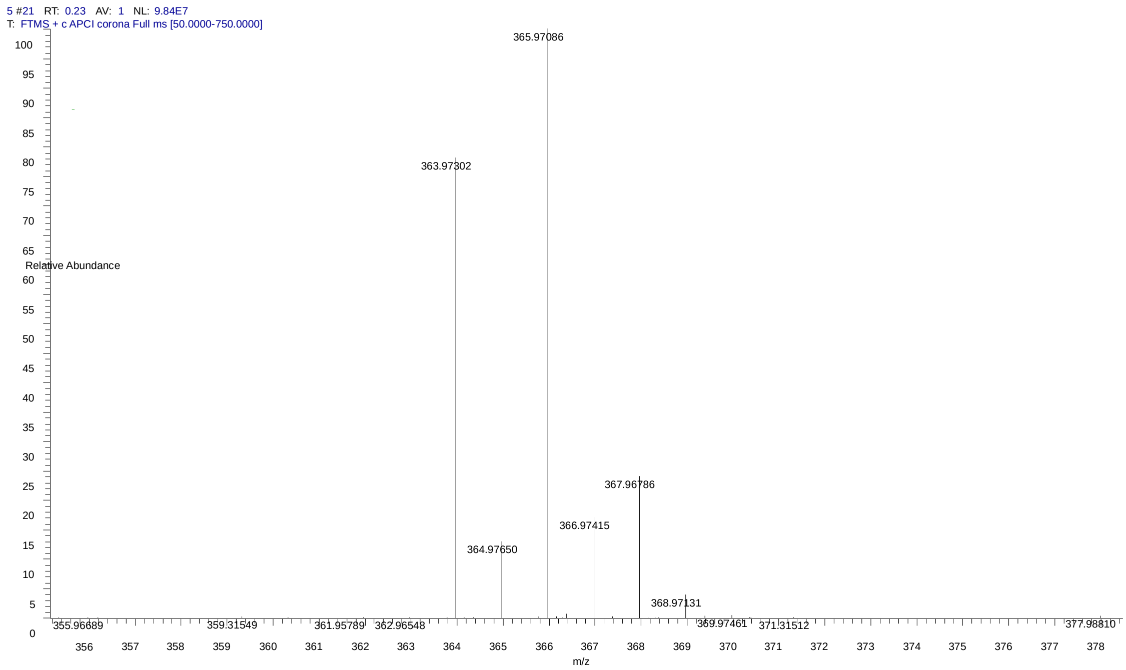

HRMS(APCI) of compound **10g**

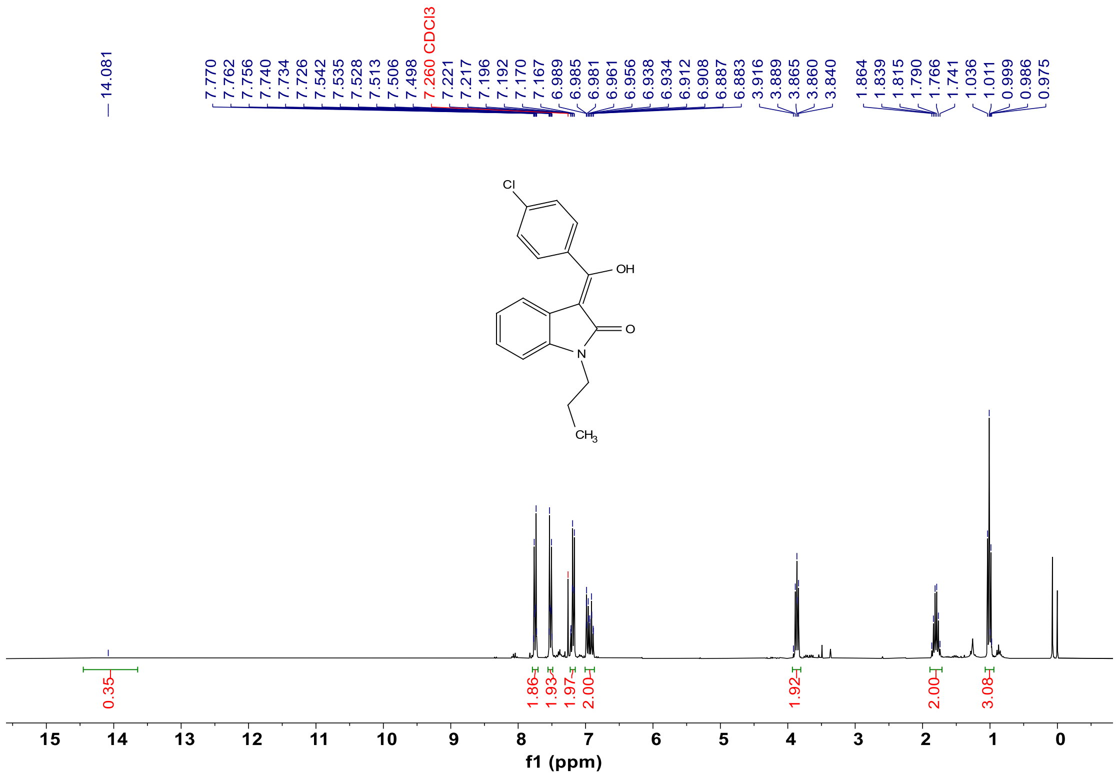

^{1}H NMR spectrum of compound **10h** (300 MHz, $CDCl_3$)

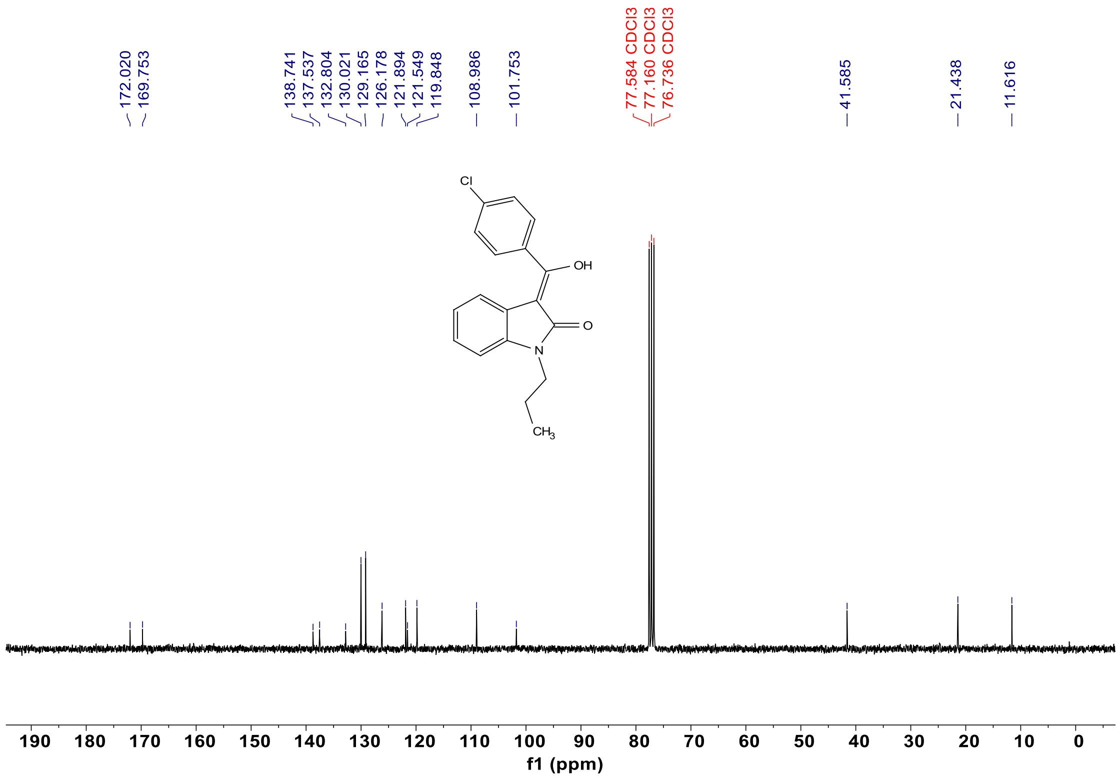

^{13}C NMR spectrum of compound **10h** (75 MHz, $CDCl_3$)

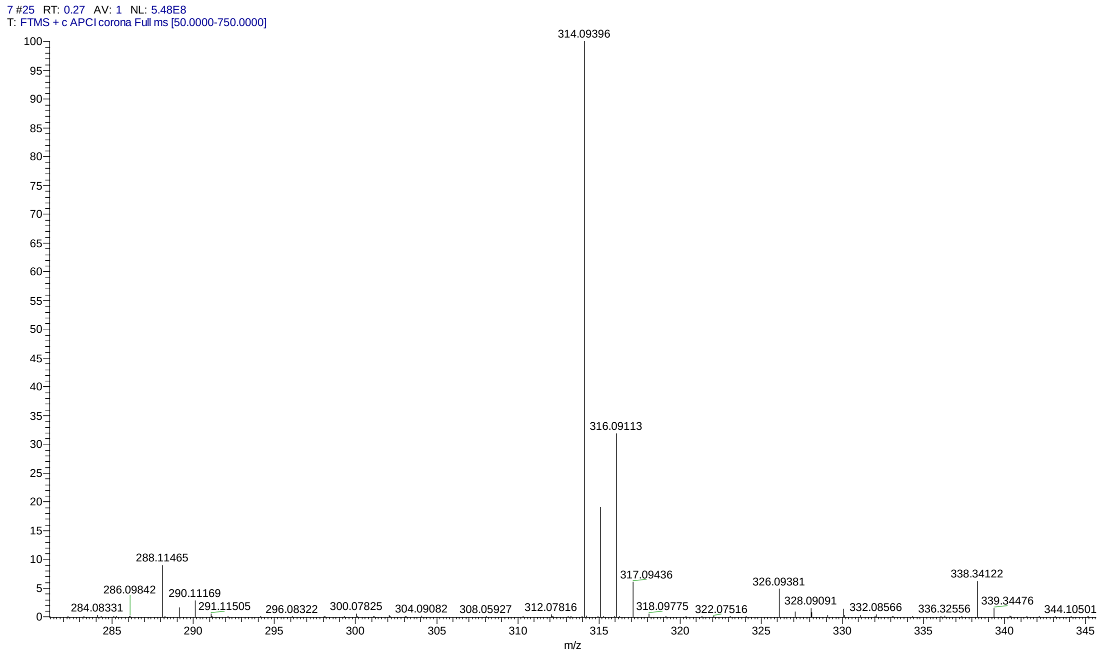

HRMS(APCI) of compound **10h**

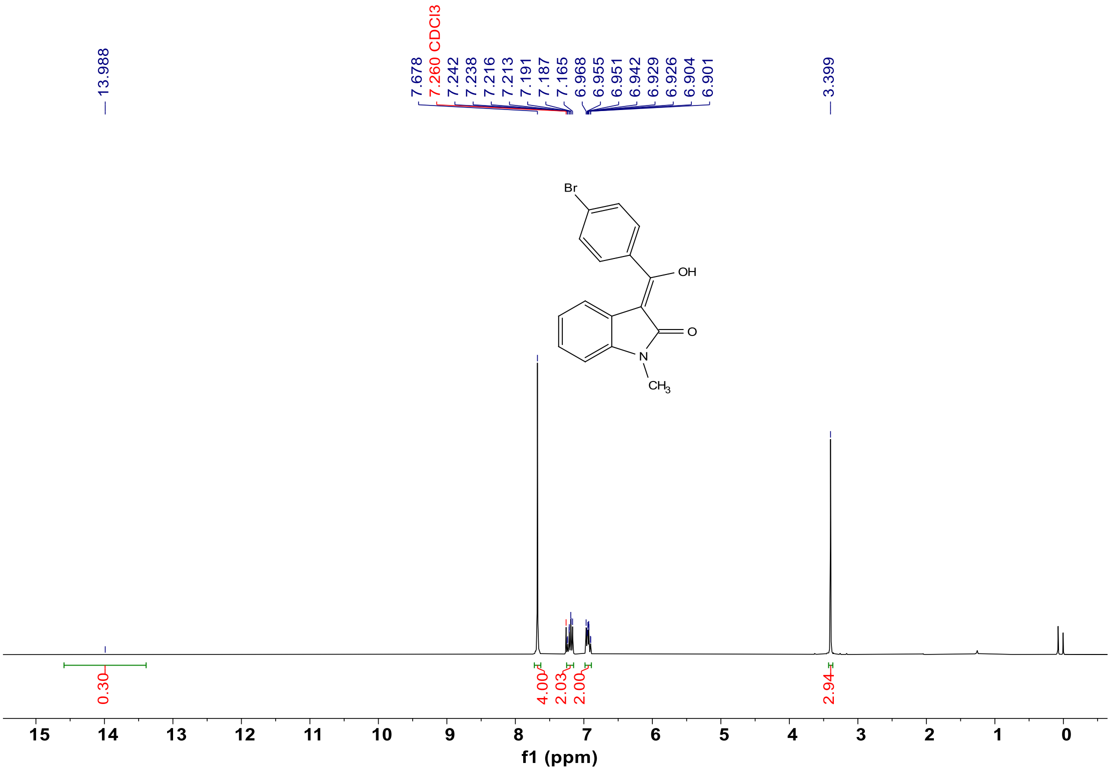

^{1}H NMR spectrum of compound **10i** (300 MHz, $CDCl_3$)

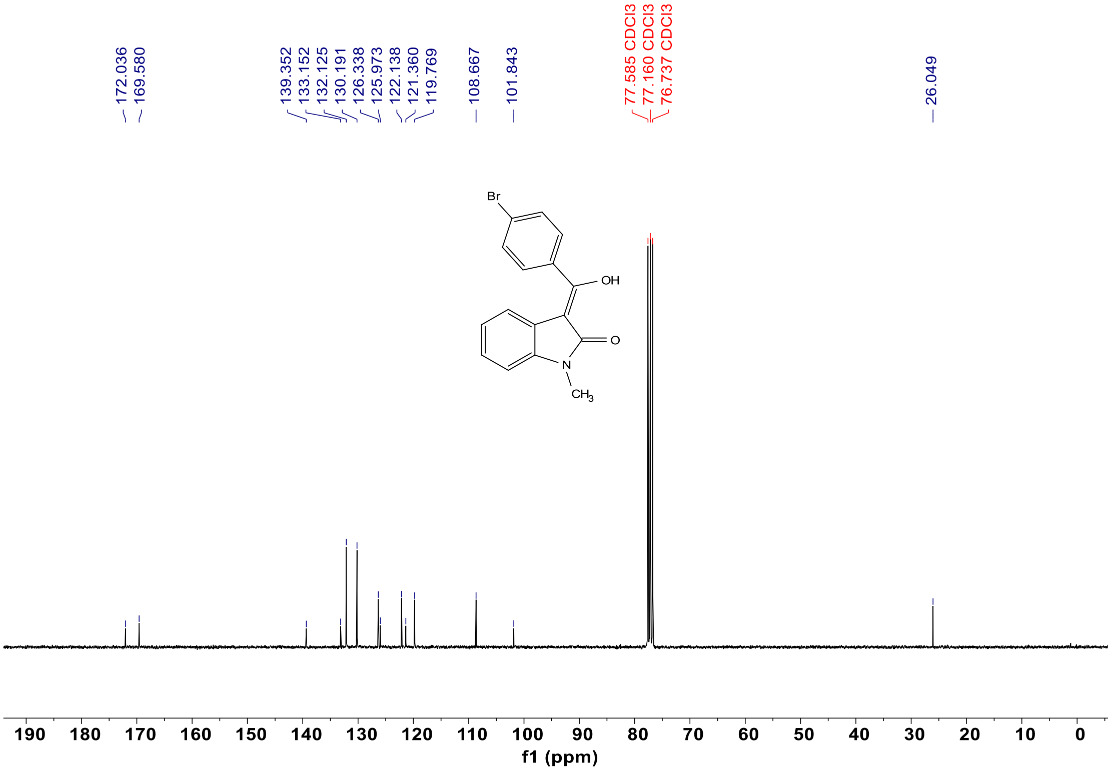

^{13}C NMR spectrum of compound **10i** (75 MHz, $CDCl_3$)

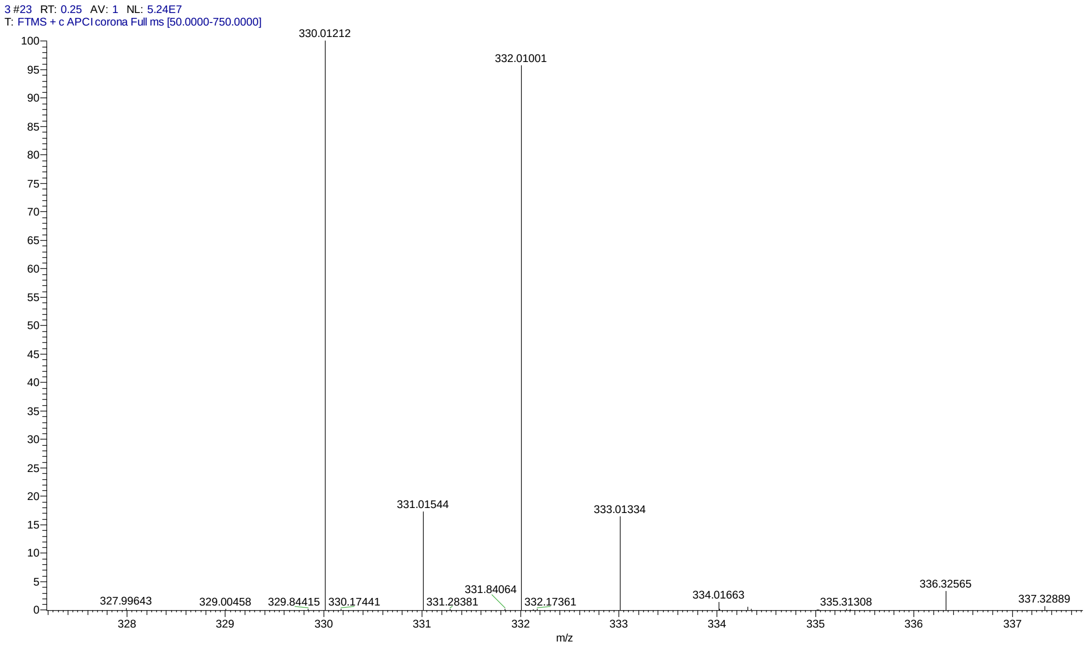

HRMS(APCI) of compound **10i**
